# Supplementary material for: Depressive symptoms and cardiovascular disease: a population-based study of older adults in rural Burkina Faso
Source: BMJ Open. 2020 Dec 22;10(12):e038199. doi: 10.1136/bmjopen-2020-038199 (PMC7757460; doi:10.1136/bmjopen-2020-038199)
Supplement: Supplementary data [file bmjopen-2020-038199supp001.pdf]

SUPPLEMENTARY MATERIAL

Supplementary Figure 1: This figure shows the distribution of the mean raw PHQ-9 values by single interviewers. The differences between interviewers' PHQ-9 values were interpreted as an interviewer effect during analyses. We decided to standardize the results on the interviewer level and add them up afterwards.

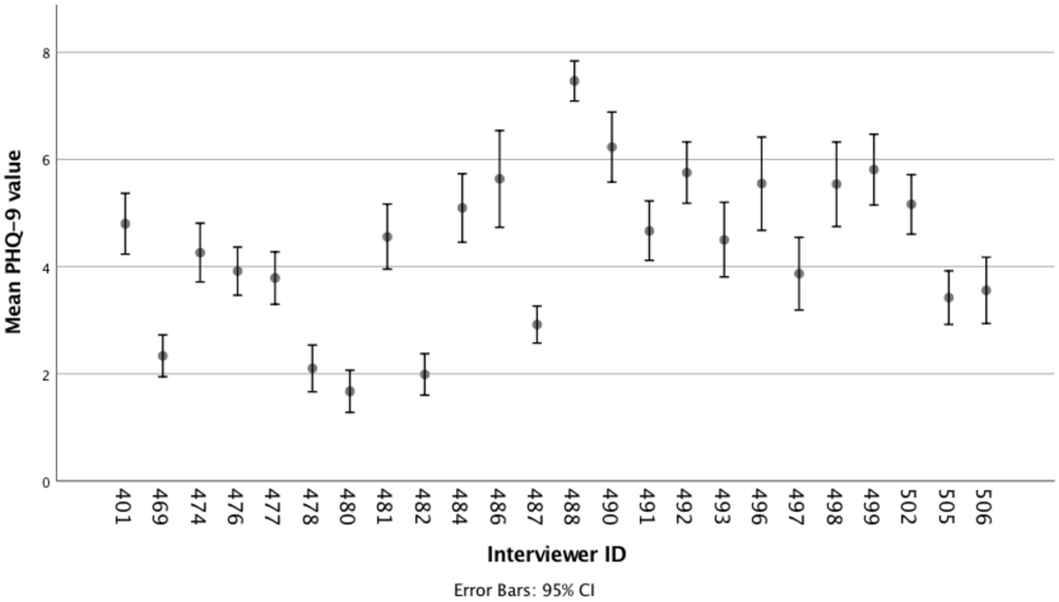

Supplementary Figure 2: The figure shows the standardized PHQ-9 values of one of the interviewers (401) and the normal distribution curve. We z-scored the PHQ-9 results for this interviewer with the standardization function in SPSS. Each value was calculated by dividing the difference between the value and the mean by the standard deviation. This was done for all interviewers separately. Afterwards, all values were included in the outcome variable of the z-scored PHQ-9 score.

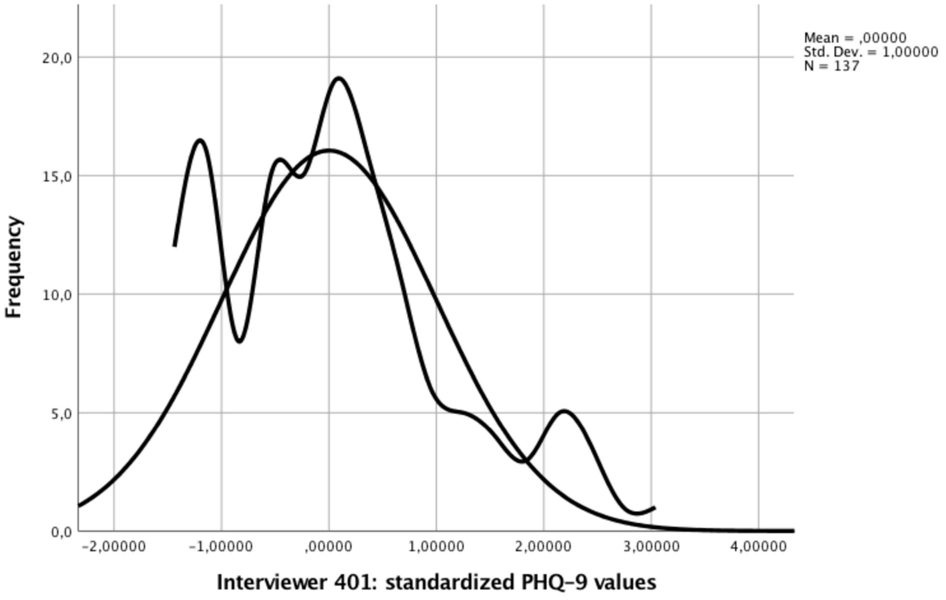

Supplementary Table 1: Distribution of the z-scored PHQ-9 values

		PHQ-9 value	CI lower	CI upper	2 Std. Dev.
m	40-44	-0,414	-0,493	-0,336	0,078
w	40-44	-0,160	-0,252	-0,067	0,092
m	45-49	-0,403	-0,496	-0,311	0,092
w	45-49	-0,087	-0,196	0,022	0,109
m	50-54	-0,259	-0,359	-0,158	0,100
w	50-54	0,025	-0,084	0,135	0,110
m	55-59	-0,135	-0,285	0,015	0,150
w	55-59	0,162	0,030	0,293	0,131
m	60-64	-0,016	-0,175	0,142	0,159
w	60-64	0,428	0,284	0,572	0,144
m	65-69	0,297	0,111	0,484	0,186
w	65-69	0,552	0,393	0,710	0,159
m	70-74	0,369	0,085	0,653	0,284
w	70-74	0,571	0,337	0,806	0,235
m	75+	0,881	0,553	1,210	0,328
w	75+	0,795	0,541	1,050	0,254

**Supplementary Table 2:** Prevalence of the metabolic syndrome				
		Proportion	CI lower	CI upper
m	40-44	0,082	0,054	0,118
w	40-44	0,120	0,083	0,166
m	45-49	0,150	0,110	0,197
w	45-49	0,183	0,133	0,242
m	50-54	0,103	0,064	0,155
w	50-54	0,210	0,158	0,271
m	55-59	0,101	0,059	0,159
w	55-59	0,244	0,183	0,315
m	60-64	0,140	0,079	0,224
w	60-64	0,190	0,130	0,263
m	65-69	0,082	0,034	0,162
w	65-69	0,274	0,195	0,364
m	70-74	0,097	0,036	0,199
w	70-74	0,273	0,177	0,386
m	75+	0,057	0,012	0,157
w	75+	0,282	0,186	0,395

**Supplementary Table 3:** Prevalence of a positive screening result for pre-diabetes				
		Proportion	CI lower	CI upper
m	40-44	0,413	0,351	0,476
w	40-44	0,378	0,315	0,444
m	45-49	0,416	0,351	0,483
w	45-49	0,468	0,395	0,542
m	50-54	0,503	0,423	0,583
w	50-54	0,503	0,430	0,576
m	55-59	0,392	0,308	0,482
w	55-59	0,532	0,451	0,611
m	60-64	0,442	0,328	0,559
w	60-64	0,425	0,338	0,516
m	65-69	0,444	0,319	0,575
w	65-69	0,473	0,377	0,570
m	70-74	0,481	0,340	0,624
w	70-74	0,515	0,389	0,640
m	75+	0,591	0,432	0,737
w	75+	0,515	0,390	0,638

**Supplementary Table 4:** Prevalence of a positive screening result for diabetes mellitus				
		Proportion	CI lower	CI upper
m	40-44	0,028	0,011	0,058
w	40-44	0,049	0,025	0,085
m	45-49	0,065	0,036	0,107
w	45-49	0,072	0,039	0,120
m	50-54	0,058	0,027	0,108
w	50-54	0,061	0,031	0,107
m	55-59	0,080	0,039	0,142
w	55-59	0,069	0,034	0,124
m	60-64	0,097	0,040	0,190
w	60-64	0,105	0,057	0,173
m	65-69	0,035	0,004	0,121
w	65-69	0,107	0,055	0,183
m	70-74	0,021	0,001	0,113
w	70-74	0,125	0,056	0,232
m	75+	0,119	0,040	0,256
w	75+	0,111	0,046	0,216

**Supplementary Table 5:** Prevalence of a positive screening result for hypertension gr. II				
		Proportion	CI lower	CI upper
m	40-44	0,182	0,144	0,225
w	40-44	0,169	0,128	0,217
m	45-49	0,227	0,182	0,277
w	45-49	0,222	0,172	0,279
m	50-54	0,235	0,181	0,297
w	50-54	0,274	0,220	0,334
m	55-59	0,306	0,241	0,378
w	55-59	0,294	0,232	0,362
m	60-64	0,344	0,261	0,434
w	60-64	0,362	0,291	0,438
m	65-69	0,355	0,265	0,454
w	65-69	0,407	0,325	0,493
m	70-74	0,333	0,227	0,454
w	70-74	0,380	0,281	0,488
m	75+	0,379	0,262	0,507
w	75+	0,442	0,345	0,543

Supplementary Table 6: Linear regression model 1 (Metabolic syndrome)

Dependent variable: Z-scored PHQ-9

		Model 1 (n=2524)			
		beta	CI low	CI high	p
Age	40-44	-	-	-	-
	45-49	.007	-.037	.050	.773
	50-54	.058	.014	.098	.010
	55-59	.098	.053	.136	<.001
	60-64	.144	.101	.184	<.001
	65-69	.182	.139	.221	<.001
	70-74	.147	.104	.183	<.001
	75+	.251	.215	.298	<.001
Sex (ref: male)		.115	.074	.151	<.001
Living in the countryside		-.052	-.088	-.011	.013
Currently married		-.020	-.065	.022	.340
Education	None				
	Some	.018	-.020	.054	.360
	Primary & more	-.049	-.088	-.009	.016
Ethnicity	Dafin				
	Bwama	.017	-.022	.053	.430
	Mossi	.007	-.028	.040	.734
	Other	.022	-.024	.082	.280
Wealth quintile	1				
	2	-.030	-.074	.016	.208
	3	-.051	-.096	-.003	.038
	4	-.058	-.103	-.009	.019
	5	-.083	-.135	-.033	.001
**Metabolic syndrome**		-.004	-.040	.033	.847

Supplementary Table 7: Linear regression model 2
Abdominal Obesity

Dependent variable: Z-scored PHQ-9

		Model 2 (n=2970)			
		beta	CI low	CI high	p
Age	40-44	-	-	-	-
	45-49	.017	-.024	.058	.406
	50-54	.059	.019	.099	.004
	55-59	.101	.062	.140	<.001
	60-64	.143	.104	.181	<.001
	65-69	.189	.150	.226	<.001
	70-74	.164	.126	.200	<.001
	75+	.239	.200	.275	<.001
Sex (ref: male)		.118	.081	.153	<.001
Living in the countryside		-.047	-.078	-.008	.016
Currently married		-.028	-.072	.010	.143
Education	None				
	Some	.019	-.016	.053	.288
	Primary & more	-.033	-.071	.003	.072
Ethnicity	Dafin				
	Bwama	.021	-.017	.055	.302
	Mossi	-.001	-.033	.031	.944
	Other	.026	-.015	.080	.180
Wealth quintile	1				
	2	-.020	-.062	.023	.372
	3	-.056	-.100	-.012	.013
	4	-.066	-.110	-.021	.004
	5	-.087	-.135	-.040	<.001
**Abdominal Obesity**		.007	-.028	.042	.680

Supplementary Table 8: Linear regression model 3 (Hypertriglyceridemia)

Dependent variable: Z-scored PHQ-9

		Model 3 (n=2890)			
		beta	CI low	CI high	p
Age	40-44	-	-	-	-
	45-49	.013	-.028	.055	.531
	50-54	.064	.022	.104	.002
	55-59	.098	.057	.137	<.001
	60-64	.144	.103	.182	<.001
	65-69	.186	.144	.221	<.001
	70-74	.159	.120	.194	<.001
	75+	.243	.203	.280	<.001
Sex (ref: male)		.115	.078	.150	<.001
Living in the countryside		-.052	-.083	-.012	.009
Currently married		-.029	-.073	.010	.141
Education	None				
	Some	.017	-.018	.052	.330
	Primary & more	-.041	-.079	-.004	.029
Ethnicity	Dafin				
	Bwama	.015	-.023	.050	.456
	Mossi	.001	-.031	.034	.938
	Other	.025	-.017	.080	.200
Wealth quintile	1				
	2	-.024	-.067	.020	.285
	3	-.054	-.099	-.010	.017
	4	-.069	-.113	-.023	.003
	5	-.083	-.132	-.036	.001
**Hypertriglyceridemia**		-.006	-.040	.029	.742

Supplementary Table 9: Linear regression model 4
(Reduced HDL-cholesterol)

Dependent variable: Z-scored PHQ-9

		Model 4 (n=2330)			
		beta	CI low	CI high	p
Age	40-44	-	-	-	-
	45-49	.019	-.028	.065	.425
	50-54	.061	.015	.106	.009
	55-59	.110	.063	.151	<.001
	60-64	.154	.107	.193	<.001
	65-69	.185	.141	.227	<.001
	70-74	.170	.124	.206	<.001
	75+	.234	.196	.283	<.001
Sex (ref: male)		.134	.091	.174	<.001
Living in the countryside		-.028	-.078	.016	.193
Currently married		-.031	-.079	.013	.158
Education	None				
	Some	.004	-.034	.042	.843
	Primary & more	-.035	-.084	.006	.092
Ethnicity	Dafin				
	Bwama	.007	-.032	.045	.745
	Mossi	-.016	-.051	.022	.437
	Other	.010	-.048	.080	.620
Wealth quintile	1				
	2	-.035	-.080	.012	.147
	3	-.059	-.106	-.010	.017
	4	-.075	-.121	-.025	.003
	5	-.072	-.133	-.026	.004
**Reduced HDL-cholesterol**		-.027	-.067	.012	.177

Supplementary Table 10: Linear regression model 5 (Hypertension grade I)

Dependent variable: Z-scored PHQ-9

		Model 5 (n=2970)			
		beta	CI low	CI high	p
Age	40-44	-	-	-	-
	45-49	.016	-.025	.057	.441
	50-54	.057	.017	.097	.006
	55-59	.098	.058	.137	<.001
	60-64	.139	.099	.177	<.001
	65-69	.185	.145	.222	<.001
	70-74	.161	.123	.197	<.001
	75+	.235	.196	.271	<.001
Sex (ref: male)		.119	.082	.154	<.001
Living in the countryside		-.046	-.077	-.007	.020
Currently married		-.028	-.072	.010	.141
Education	None				
	Some	.020	-.015	.054	.257
	Primary & more	-.034	-.071	.002	.067
Ethnicity	Dafin				
	Bwama	.020	-.018	.054	.326
	Mossi	.000	-.032	.032	.982
	Other	.027	-.014	.081	.164
Wealth quintile	1				
	2	-.019	-.062	.024	.379
	3	-.055	-.099	-.011	.014
	4	-.066	-.110	-.022	.004
	5	-.087	-.134	-.040	<.001
**Hypertension grade I**		.028	-.007	.062	.113

Supplementary Table 11: Linear regression model 6
(Fasting Plasma Glucose > 100 mg/dl)

Dependent variable: Z-scored PHQ-9

		Model 6 (n=2038)			
		beta	CI low	CI high	p
Age	40-44	-	-	-	-
	45-49	.002	-.046	.049	.939
	50-54	.049	.000	.093	.048
	55-59	.102	.053	.144	<.001
	60-64	.143	.096	.188	<.001
	65-69	.192	.144	.236	<.001
	70-74	.125	.078	.164	<.001
	75+	.246	.206	.297	<.001
Sex (ref: male)		.114	.069	.153	<.001
Living in the countryside		-.077	-.114	-.028	.001
Currently married		-.016	-.065	.031	.488
Education	None				
	Some	.024	-.018	.068	.257
	Primary & more	-.066	-.107	-.021	.004
Ethnicity	Dafin				
	Bwama	-.003	-.045	.040	.904
	Mossi	.010	-.028	.045	.663
	Other	.030	-.019	.094	.190
Wealth quintile	1				
	2	-.028	-.078	.024	.295
	3	-.058	-.110	-.004	.035
	4	-.077	-.126	-.021	.006
	5	-.101	-.154	-.042	.001
**Fasting Plasma Glucose > 100 mg/dl**		-.022	-.061	.019	.298

Supplementary Table 12: Linear regression model 7
(Hypertension grade II)

Dependent variable: Z-scored PHQ-9

		Model 7 (n=2970)			
		beta	CI low	CI high	p
Age	40-44	-	-	-	-
	45-49	.017	-.024	.058	.422
	50-54	.059	.018	.099	.004
	55-59	.100	.060	.139	<.001
	60-64	.141	.102	.179	<.001
	65-69	.188	.148	.224	<.001
	70-74	.163	.125	.199	<.001
	75+	.237	.197	.273	<.001
Sex (ref: male)		.118	.082	.154	<.001
Living in the countryside		-.047	-.078	-.008	.016
Currently married		-.028	-.072	.010	.143
Education	None				
	Some	.019	-.015	.054	.274
	Primary & more	-.033	-.070	.003	.072
Ethnicity	Dafin				
	Bwama	.020	-.017	.054	.309
	Mossi	-.001	-.033	.031	.946
	Other	.026	-.015	.081	.176
Wealth quintile	1				
	2	-.019	-.062	.024	.386
	3	-.055	-.099	-.011	.014
	4	-.066	-.109	-.021	.004
	5	-.086	-.133	-.039	<.001
**Hypertension grade II**		.014	-.020	.048	.427

Supplementary Table 13: Linear regression model 8 (Pre-diabetes)

Dependent variable: Z-scored PHQ-9

		Model 8 (n=2143)			
		beta	CI low	CI high	p
Age	40-44	-	-	-	-
	45-49	.005	-.042	.052	.838
	50-54	.050	.002	.093	.040
	55-59	.099	.050	.139	<.001
	60-64	.142	.096	.187	<.001
	65-69	.204	.155	.244	<.001
	70-74	.128	.082	.166	<.001
	75+	.240	.198	.287	<.001
Sex (ref: male)		.110	.066	.148	<.001
Living in the countryside		-.070	-.106	-.022	.003
Currently married		-.018	-.065	.028	.432
Education	None				
	Some	.026	-.015	.067	.216
	Primary & more	-.071	-.110	-.026	.001
Ethnicity	Dafin				
	Bwama	.000	-.041	.041	.999
	Mossi	.007	-.030	.042	.756
	Other	.038	-.008	.102	.097
Wealth quintile	1				
	2	-.024	-.073	.027	.368
	3	-.056	-.106	-.003	.040
	4	-.067	-.115	-.013	.014
	5	-.097	-.148	-.039	.001
**Pre-diabetes**		.005	-.034	.043	.819

Supplementary Table 14: Linear regression model 9 (Diabetes)

Dependent variable: Z-scored PHQ-9

		Model 9 (n=2043)			
		beta	CI low	CI high	p
Age	40-44	-	-	-	-
	45-49	.001	-.047	.048	.983
	50-54	.048	.000	.092	.051
	55-59	.104	.055	.146	<.001
	60-64	.143	.096	.188	<.001
	65-69	.191	.143	.235	<.001
	70-74	.123	.077	.163	<.001
	75+	.246	.204	.295	<.001
Sex (ref: male)		.112	.066	.150	<.001
Living in the countryside		-.081	-.118	-.031	.001
Currently married		-.017	-.065	.030	.474
Education	None				
	Some	.031	-.011	.075	.147
	Primary & more	-.066	-.105	-.020	.004
Ethnicity	Dafin				
	Bwama	-.010	-.051	.033	.672
	Mossi	.008	-.030	.043	.735
	Other	.031	-.018	.095	.179
Wealth quintile	1				
	2	-.033	-.082	.019	.227
	3	-.069	-.120	-.014	.013
	4	-.083	-.132	-.027	.003
	5	-.105	-.158	-.045	<.001
**Diabetes**		-.001	-.040	.039	.972

Supplementary Table 15: Linear regression model 10
(Self-reported hypertension)

Dependent variable: Z-scored PHQ-9

		Model 10 (n=2972)			
		beta	CI low	CI high	p
Age	40-44	-	-	-	-
	45-49	.016	-.025	.057	.434
	50-54	.056	.015	.095	.007
	55-59	.093	.053	.131	<.001
	60-64	.143	.103	.180	<.001
	65-69	.179	.140	.217	<.001
	70-74	.152	.115	.190	<.001
	75+	.235	.196	.271	<.001
Sex (ref: male)		.119	.083	.155	<.001
Living in the countryside		-.044	-.074	-.005	.027
Currently married		-.036	-.080	.003	.066
Education	None				
	Some	.017	-.017	.051	.329
	Primary & more	-.027	-.062	.010	.152
Ethnicity	Dafin				
	Bwama	.017	-.021	.051	.404
	Mossi	.000	-.032	.032	.999
	Other	.026	-.014	.081	.172
Wealth quintile	1				
	2	-.015	-.058	.029	.512
	3	-.050	-.093	-.005	.028
	4	-.060	-.104	-.015	.009
	5	-.087	-.133	-.039	<.001
**Self-reported hypertension**		.032	-.003	.066	.071

Supplementary Table 16: Linear regression model 11
(Self-reported diabetes)

Dependent variable: Z-scored PHQ-9

		Model 11 (n=2991)			
		beta	CI low	CI high	p
Age	40-44	-	-	-	-
	45-49	.013	-.028	.053	.541
	50-54	.058	.018	.097	.005
	55-59	.098	.057	.135	<.001
	60-64	.144	.104	.181	<.001
	65-69	.182	.143	.219	<.001
	70-74	.154	.117	.190	<.001
	75+	.233	.194	.269	<.001
Sex (ref: male)		.123	.086	.158	<.001
Living in the countryside		-.044	-.074	-.005	.024
Currently married		-.036	-.080	.002	.062
Education	None				
	Some	.020	-.014	.054	.250
	Primary & more	-.024	-.060	.012	.195
Ethnicity	Dafin				
	Bwama	.016	-.022	.050	.438
	Mossi	-.001	-.032	.031	.965
	Other	.023	-.018	.076	.230
Wealth quintile	1				
	2	-.015	-.058	.028	.500
	3	-.054	-.097	-.010	.016
	4	-.065	-.108	-.020	.004
	5	-.085	-.130	-.037	<.001
**Self-reported diabetes**		.004	-.029	.038	.809

Supplementary Table 17: Linear regression model 12
(Self-reported heart disease)

Dependent variable: Z-scored PHQ-9

		Model 12 (n=3001)			
		beta	CI low	CI high	p
Age	40-44	-	-	-	-
	45-49	.012	-.029	.052	.567
	50-54	.056	.016	.095	.006
	55-59	.094	.054	.132	<.001
	60-64	.143	.105	.181	<.001
	65-69	.181	.143	.219	<.001
	70-74	.161	.124	.197	<.001
	75+	.236	.197	.272	<.001
Sex (ref: male)		.112	.076	.148	<.001
Living in the countryside		-.047	-.077	-.008	.016
Currently married		-.029	-.072	.009	.128
Education	None				
	Some	.018	-.017	.052	.314
	Primary & more	-.026	-.062	.010	.159
Ethnicity	Dafin				
	Bwama	.016	-.021	.051	.410
	Mossi	-.002	-.033	.030	.931
	Other	.021	-.021	.074	.272
Wealth quintile	1				
	2	-.021	-.063	.022	.338
	3	-.058	-.102	-.015	.009
	4	-.071	-.115	-.027	.002
	5	-.092	-.138	-.045	<.001
**Self-reported heart disease**		.081	.047	.114	<.001

Supplementary Table 18: Linear regression model 13
(Self-reported stroke)

Dependent variable: Z-scored PHQ-9

		Model 13 (n=3026)			
		beta	CI low	CI high	p
Age	40-44	-	-	-	-
	45-49	.015	-.025	.056	.455
	50-54	.058	.018	.097	.004
	55-59	.094	.055	.133	<.001
	60-64	.143	.104	.181	<.001
	65-69	.185	.147	.222	<.001
	70-74	.162	.125	.198	<.001
	75+	.236	.197	.272	<.001
Sex (ref: male)		.122	.086	.157	<.001
Living in the countryside		-.049	-.079	-.010	.012
Currently married		-.031	-.074	.008	.110
Education	None				
	Some	.019	-.015	.053	.282
	Primary & more	-.027	-.062	.009	.147
Ethnicity	Dafin				
	Bwama	.019	-.018	.053	.344
	Mossi	-.001	-.032	.031	.960
	Other	.023	-.018	.076	.224
Wealth quintile	1				
	2	-.018	-.061	.024	.401
	3	-.055	-.098	-.011	.014
	4	-.065	-.109	-.021	.004
	5	-.087	-.134	-.040	<.001
**Self-reported stroke**		.038	.005	.071	.024

Supplementary Table 19: Linear regression model 14
(Sudden drooping)

Dependent variable: Z-scored PHQ-9

		Model 14 (n=3026)			
		beta	CI low	CI high	p
Age	40-44	-	-	-	-
	45-49	.013	-.027	.054	.519
	50-54	.054	.014	.093	.008
	55-59	.094	.055	.132	<.001
	60-64	.137	.099	.175	<.001
	65-69	.183	.144	.220	<.001
	70-74	.157	.120	.193	<.001
	75+	.235	.196	.271	<.001
Sex (ref: male)		.120	.084	.155	<.001
Living in the countryside		-.049	-.078	-.010	.012
Currently married		-.031	-.075	.006	.100
Education	None				
	Some	.019	-.015	.053	.284
	Primary & more	-.027	-.062	.009	.147
Ethnicity	Dafin				
	Bwama	.015	-.021	.049	.438
	Mossi	-.001	-.033	.030	.947
	Other	.022	-.019	.075	.241
Wealth quintile	1				
	2	-.017	-.059	.026	.446
	3	-.056	-.099	-.013	.012
	4	-.066	-.109	-.022	.003
	5	-.087	-.133	-.041	<.001
**Sudden drooping**		.090	.057	.123	<.001

Supplementary Table 20: Linear regression model 15 (Sudden numbness)

Dependent variable: Z-scored PHQ-9

		Model 15 (n=3026)			
		beta	CI low	CI high	p
Age	40-44	-	-	-	-
	45-49	.017	-.024	.057	.419
	50-54	.059	.019	.098	.004
	55-59	.095	.056	.133	<.001
	60-64	.135	.096	.172	<.001
	65-69	.178	.140	.215	<.001
	70-74	.155	.118	.190	<.001
	75+	.227	.189	.263	<.001
Sex (ref: male)		.117	.081	.151	<.001
Living in the countryside		-.051	-.080	-.012	.008
Currently married		-.034	-.078	.003	.071
Education	None				
	Some	.016	-.018	.049	.366
	Primary & more	-.026	-.062	.009	.147
Ethnicity	Dafin				
	Bwama	.017	-.019	.051	.380
	Mossi	.001	-.031	.032	.965
	Other	.020	-.022	.072	.291
Wealth quintile	1				
	2	-.019	-.061	.023	.378
	3	-.061	-.104	-.017	.006
	4	-.072	-.115	-.028	.001
	5	-.097	-.143	-.051	<.001
**Sudden numbness**		.136	.102	.168	<.001

Supplementary Table 21: Linear regression model 16
(Sudden slurring)

Dependent variable: Z-scored PHQ-9

		Model 16 (n=3026)			
		beta	CI low	CI high	p
Age	40-44	-	-	-	-
	45-49	.016	-.025	.055	.448
	50-54	.056	.017	.096	.005
	55-59	.093	.054	.131	<.001
	60-64	.134	.096	.172	<.001
	65-69	.175	.137	.212	<.001
	70-74	.155	.118	.190	<.001
	75+	.227	.189	.263	<.001
Sex (ref: male)		.117	.081	.151	<.001
Living in the countryside		-.052	-.081	-.013	.007
Currently married		-.037	-.081	.000	.050
Education	None				
	Some	.019	-.015	.053	.264
	Primary & more	-.029	-.064	.007	.113
Ethnicity	Dafin				
	Bwama	.017	-.019	.051	.379
	Mossi	.001	-.030	.032	.954
	Other	.017	-.026	.068	.374
Wealth quintile	1				
	2	-.022	-.064	.020	.312
	3	-.060	-.103	-.017	.006
	4	-.072	-.116	-.029	.001
	5	-.091	-.136	-.044	<.001
**Sudden slurring**		.144	.110	.176	<.001

Supplementary Table 22: Linear regression model 17
(Self-reported metabolic syndrome)

Dependent variable: Z-scored PHQ-9

		Model 17 (n=2948)			
		beta	CI low	CI high	p
Age	40-44	-	-	-	-
	45-49	.014	-.027	.055	.497
	50-54	.055	.014	.094	.008
	55-59	.092	.052	.130	<.001
	60-64	.141	.101	.178	<.001
	65-69	.174	.135	.212	<.001
	70-74	.151	.114	.188	<.001
	75+	.230	.190	.266	<.001
Sex (ref: male)		.117	.080	.152	<.001
Living in the countryside		-.039	-.070	.000	.049
Currently married		-.039	-.083	-.001	.045
Education	None				
	Some	.018	-.017	.052	.314
	Primary & more	-.029	-.064	.008	.124
Ethnicity	Dafin				
	Bwama	.015	-.022	.049	.460
	Mossi	-.001	-.033	.031	.956
	Other	.024	-.017	.079	.201
Wealth quintile	1				
	2	-.016	-.059	.027	.459
	3	-.051	-.095	-.007	.024
	4	-.066	-.110	-.021	.004
	5	-.091	-.137	-.042	<.001
**Self-reported metabolic syndrome**		.070	.035	.104	<.001

Supplementary Table 23: Descriptive statistics of the self-reported diagnosis of hypertension and diabetes within the whole sample

	Total	Female	Male
**Self-reported diagnosis of hypertension**	*n* = 3007<br>507 (17.1%)	*n* = 1513<br>294 (19.8%)	*n* = 1494<br>213 (14.4%)
**Self-reported diagnosis of diabetes**	*n* = 2993<br>70 (2.3%)	*n* = 1505<br>32 (2.1%)	*n* = 1488<br>38 (2.6%)

Supplementary Table 24: Descriptive statistics of the self-reported diagnosis of hypertension and diabetes within the affected* part of the sample

	Total	Female	Male
**Self-reported diagnosis of hypertension**	*n* = 1674<br>346 (21.0%)	*n* = 841<br>203 (24.6%)	*n* = 832<br>143 (17.3%)
**Self-reported diagnosis of diabetes**	*n* = 143<br>20 (14.0%)	*n* = 85<br>11 (13.0%)	*n* = 58<br>9 (15.5%)

*In case of Hypertension: Systolic BP >130 mmHg or Diastolic BP >80 mmHg
Diabetes: Fasting Glucose >126 mg/dl, random glucose >200 mg/dl or HbA1c >6.5%

Supplementary Table 25: Measuring the effect of a diagnosis of hypertension/diabetes given by a health care worker among hypertensive/diabetic part of the population.
Given in the table are the standardized coefficients of multiple linear regression of the z-scored PHQ-9 score on self-reported diagnosis of hypertension within the hypertensive population[a] and diagnosis of diabetes within the diabetic population[b].

	Standardized beta coefficient	p-value
**Ever been diagnosed with hypertension**	0.033	0.061
**Ever been diagnosed with diabetes**	-0.025	0.762

All variables used in separate models controlling for age, sex, gender, place of residence, education, ethnicity and wealth quintile.
[a] Systolic BP >130 mmHg or Diastolic BP >80 mmHg
[b] Fasting Glucose >126 mg/dl, random glucose >200 mg/dl or HbA1c >6.5%
