# Supplementary material for: Depressive symptoms and cardiovascular disease: a population-based study of older adults in rural Burkina Faso
Source: BMJ Open. 2020 Dec 22;10(12):e038199. doi: 10.1136/bmjopen-2020-038199 (PMC7757460; doi:10.1136/bmjopen-2020-038199)
Supplement: Author's manuscript [file bmjopen-2020-038199.draft_revisions.pdf]

BMJ Open

BMJ Open is committed to open peer review. As part of this commitment we make the peer review history of every article we publish publicly available.

When an article is published we post the peer reviewers' comments and the authors' responses online. We also post the versions of the paper that were used during peer review. These are the versions that the peer review comments apply to.

The versions of the paper that follow are the versions that were submitted during the peer review process. They are not the versions of record or the final published versions. They should not be cited or distributed as the published version of this manuscript.

BMJ Open is an open access journal and the full, final, typeset and author-corrected version of record of the manuscript is available on our site with no access controls, subscription charges or pay-per-view fees (http://bmjopen.bmj.com).

If you have any questions on BMJ Open's open peer review process please email info.bmjopen@bmj.com

BMJ Open

Depressive symptoms and cardiovascular disease: a population-based study of older adults in rural Burkina Faso

Journal:	*BMJ Open*
Manuscript ID	bmjopen-2020-038199
Article Type:	Original research
Date Submitted by the Author:	03-Mar-2020
Complete List of Authors:	Brinkmann, Ben; Heidelberg University, Heidelberg Institute of Global Health<br>Payne, Collin F.; • School of Demography, The Australian National University, Canberra, Australia, School of Demography<br>Kohler, Iliana ; University of Pennsylvania, Population Studies Center (PSC) and Department of Sociology<br>Harling, Guy; University College London, Institute for Global Health; Harvard Center for Population and Development Studies<br>Davies, Justine ; University of Birmingham, Institute of Applied Health Research<br>Witham, Miles; AGE Research Group, NIHR Newcastle Biomedical Research Centre, Newcastle University; Newcastle Upon Tyne Hospitals NHS Foundation Trust<br>Siedner, Mark; Harvard Medical School, Department of Medicine, Massachusetts General Hospital<br>Sie, Ali; Centre de Recherche en Sante de Nouna<br>Bountogo, Mamadou; Centre de Recherche en Sante de Nouna<br>Ouermi, Lucienne; Centre de Recherche en Sante de Nouna<br>Coulibaly, Boubacar; Centre de Recherche en Sante de Nouna<br>Bärnighausen, Till ; Heidelberg University, Heidelberg Institute of Global Health; Africa Health Research Institute
Keywords:	Depression & mood disorders < PSYCHIATRY, Hypertension < CARDIOLOGY, STROKE MEDICINE, PUBLIC HEALTH, EPIDEMIOLOGY, MENTAL HEALTH

SCHOLARONE™
Manuscripts

BMJ

I, the Submitting Author has the right to grant and does grant on behalf of all authors of the Work (as defined in the below author licence), an exclusive licence and/or a non-exclusive licence for contributions from authors who are: i) UK Crown employees; ii) where BMJ has agreed a CC-BY licence shall apply, and/or iii) in accordance with the terms applicable for US Federal Government officers or employees acting as part of their official duties; on a worldwide, perpetual, irrevocable, royalty-free basis to BMJ Publishing Group Ltd ("BMJ") its licensees and where the relevant Journal is co-owned by BMJ to the co-owners of the Journal, to publish the Work in this journal and any other BMJ products and to exploit all rights, as set out in our licence.

The Submitting Author accepts and understands that any supply made under these terms is made by BMJ to the Submitting Author unless you are acting as an employee on behalf of your employer or a postgraduate student of an affiliated institution which is paying any applicable article publishing charge ("APC") for Open Access articles. Where the Submitting Author wishes to make the Work available on an Open Access basis (and intends to pay the relevant APC), the terms of reuse of such Open Access shall be governed by a Creative Commons licence – details of these licences and which Creative Commons licence will apply to this Work are set out in our licence referred to above.

Other than as permitted in any relevant BMJ Author's Self Archiving Policies, I confirm this Work has not been accepted for publication elsewhere, is not being considered for publication elsewhere and does not duplicate material already published. I confirm all authors consent to publication of this Work and authorise the granting of this licence.

For peer review only

Title:

Depressive symptoms and cardiovascular disease: a population-based study of older adults in rural Burkina Faso

Corresponding Author:

Ben Brinkmann

Im Neuenheimer Feld 672

69120 Heidelberg

Germany

ben.brinkmann@stud.uni.heidelberg.de

+49 1573 6194470

Authors:

Ben Brinkmann[1], mail: ben.brinkmann@stud.uni-heidelberg.de

Collin Payne, PhD[2], mail: collin.payne@anu.edu.au

Iliana V. Kohler, PhD[3], mail: iliana@pop.upenn.edu

Guy Harling, ScD[4,5,6,7], mail: g.harling@ucl.ac.uk

Justine I. Davies[8], mail: j.davies.6@bham.ac.uk

Miles D. Witham[9], mail: Miles.Witham@newcastle.ac.uk

Mark J. Siedner[10], mail: MSIEDNER@mgh.harvard.edu

Ali Sié, MD, PhD[11], mail: sieali.crsn@fasonet.bf

Mamadou Bountogo, MD, MSc[11], mail: drbountogo@yahoo.fr

Lucienne Ouermi, MD[11], mail: ouermil@yahoo.fr

Boubacar Coulibaly, MD[11], mail: boubacar@fasonet.bf

Till Bärnighausen, MD, ScD[1,4], mail: till.baernighausen@uni-heidelberg.de

Affiliations:

[1] Heidelberg Institute of Global Health, Heidelberg University, Germany.

[2] School of Demography, Australian National University, Canberra, Australian Capital Territory, Australia.

For peer review only

[3] Population Studies Center (PSC) and Department of Sociology, University of Pennsylvania, Philadelphia, PA, USA.

[4] Harvard Center for Population and Development Studies, Cambridge, MA, USA.

[5] Institute for Global Health, University College London, London, UK.

[6] Africa Health Research Institute, KwaZulu-Natal, South Africa

[7] MRC/WITS Rural Public Health and Health Transitions Research Unit (Agincourt), Faculty of Health Sciences, University of the Witwatersrand, Johannesburg, South Africa

[8] Institute of Applied Health Research, University of Birmingham, Birmingham, UK

[9] AGE Research Group, NIHR Newcastle Biomedical Research Centre, Newcastle University and Newcastle upon Tyne Hospitals NHS Trust, Newcastle, UK

[10] Department of Medicine, Massachusetts General Hospital, Harvard Medical School, Boston, MA, USA

[11] Centre de Recherche en Santé de Nouna, Nouna, Burkina Faso.

Word count:

4,223

For peer review only

ABSTRACT

Objectives: To contribute to the current understanding of depressive disorders in sub-Saharan African (SSA) countries by examining the association of depressive symptoms with cardiovascular and cardiometabolic conditions in a population-based study of middle-aged and older adults in rural Burkina Faso.

Setting: This study was conducted in the Nouna Health and Demographic Surveillance System (HDSS) in north-western Burkina Faso, in a mixed rural and small-town environment. The data were obtained between May and June 2018.

Participants: Consenting adults over 40 years of age (n=3,026).

Primary and secondary outcome measures: Depressive symptoms were assessed using the Patient Health Questionnaire depression module (PHQ-9). Chronic cardiometabolic conditions were assessed via a lipid panel and HbA1c measures from serum, alongside anthropometry and blood pressure measurements and a self-reported questionnaire. Multivariable linear regression was used to test the relationship between depressive symptoms and cardiovascular/cardiometabolic conditions, after controlling for sociodemographic factors.

Results: Depressive symptoms were not associated with the metabolic syndrome (standardized beta coefficient = 0.00 [95% confidence interval (CI): -0.04 to 0.03]), hypertension (beta = 0.01 [95% CI: -0.02 to 0.05]), diabetes mellitus (beta = 0.00 [95% CI: -0.04 to 0.04]), past diagnosis of elevated blood pressure or blood sugar. Prior stroke diagnosis (beta = 0.04 [95% CI: 0.01 to 0.07]) or heart disease (beta = 0.08 [95% CI: 0.05 to 0.11]) was positively associated with the standardized PHQ-9 score, as were self-reported stroke symptoms.

Conclusion: Objectively measured cardiometabolic conditions had no significant association with depressive symptoms in an older, poor, rural SSA population, in contrast to observations in high income

For peer review only

countries (HIC). However, consequences of cardiovascular disease such as stroke and heart attack were associated with depressive symptoms in older adults in Burkina Faso.

STRENGTHS TO THIS STUDY

- This study was large and population-based with a randomly selected sample, in contrast to the selective nature of prior studies about depression in Burkina Faso.
- The use of biomarker testing for cardiovascular health and the PHQ-9 depression screening instrument that has been validated in primary care settings in SSA, provides internationally comparable data.

LIMITATIONS TO THIS STUDY

- The oral translation of the study questionnaire into local languages makes it vulnerable to differences in expression during interviews.
- The cross-sectional design of the questionnaire limits our ability to determine temporal ordering of cardiovascular and mental health events.
- We were not able to clinically confirm diagnoses of hypertension, diabetes and depression in study participants.

For peer review only

KEY QUESTIONS

What is already known?

Depressive symptoms are positively associated with cardiovascular disease risk factors in HIC. Previous literature from SSA shows that these associations may not generalize to lower income countries like Burkina Faso.

What are the new findings?

Cardiovascular disease risk factors and conditions like the metabolic syndrome, hypertension, diabetes mellitus and self-reported elevated blood pressure and sugar were not asssociated with depressive symptoms in a rural, poor Burkina Faso environment. Prior diagnosis of stroke or heart disease as well as self-reported stroke symptoms were positively associated with depressive symptoms.

What do the new findings imply?

Depressive symptoms in older adults with cardiovascular disease might be prevented when detected in early stages, as well as cardiovascular conditions in adults with depressive symptoms. As accessible diagnostic and therapeutic options exist for both cardiovascular disease and depressive symptoms, this gap should be addressed by policymakers as soon as possible.

For peer review only

INTRODUCTION

The decline in communicable diseases and the ageing of populations worldwide, including low-income countries, means that cardiovascular disease (CVD) is now the leading cause of mortality worldwide, accounting for an estimated 31% of all deaths [1]. The leading cause of morbidity in the Global Burden of Disease study 2015, however, was depressive disorders [2].

In high-income contexts, CVD risk factors as summarized by the metabolic syndrome – obesity, dyslipidemia, hypertension and diabetes mellitus – are bidirectionally associated with depressive disorders [3-6]. People with depression are more likely to smoke tobacco, eat an unhealthy diet, be physically inactive and use alcohol to excess, which makes them more vulnerable to develop the metabolic syndrome [7]. Elevated activity of the hypothalamus-pituitary adrenal (HPA) axis caused by chronic stress in people with depression increases the risk for obesity, dyslipidemia and diabetes [8,9]. Alterations in the autonomic nervous system found in people with depression [10] contribute to hypertension and increased insulin resistance. Other related factors adding to the hypothesis of a shared pathophysiology are over-activated inflammatory pathways, oxidative stress, endothelial dysfunction and hormone disequilibrium [5,11-13].

CVDs such as ischemic heart disease and cerebrovascular stroke are associated with subsequent depression. While depression increases the risk of heart attack and stroke [14,15], both are also associated with higher levels of later depression [16-18]. Post-stroke depression might be a result of the brain damage from the stroke, but can also be a psychological reaction to the stroke illness and to its disabling consequences. Risk factors for depressive symptoms after stroke include stroke severity, disability after stroke, cognitive impairment, lack of social support and genetic predisposition [19,20]. Furthermore, depression worsens the outcomes of stroke [20] and myocardial infarction [21], increasing mortality and worsening long-term functional results.

In recent years, a small but growing body of evidence from sub-Saharan Africa (SSA) suggests that associations between CVD, diabetes and depression seen in HIC may not generalize to lower-income settings [22,23]. In addition, it has been found that the age pattern for depressive

For peer review only

disorders differs substantially from high-income countries. Depression prevalence in high-income countries peaks around age 60 and declines in the following decades, resulting in an inverted U-shape [24]. In contrast, in low-income contexts the prevalence of depressive symptoms appears to increase consistently with age [23,25].

In this paper, we seek to contribute to the current understanding of depressive disorders in sub-Saharan African countries by using newly collected data from Burkina Faso. Specifically, we aim to examine the potential associations of depressive symptoms with the metabolic syndrome, hypertension, diabetes mellitus and self-reported chronic conditions and stroke symptoms as well as the age pattern of depressive symptoms. Depression and mental disorders more generally are understudied in Burkina Faso and the evidence about the relationships with CVDs is inconclusive. Two recent studies examined the prevalence of depressive symptoms within pre-selected populations, Yameogo et al. [26] focused on hypertensive outpatients and Napon et al. [27] investigated depressive symptoms among people who had a stroke. Duthé et al. [28] worked with a sample of urban adults in the capital city of Ouagadougou, finding an association between broadly defined self-reported chronic conditions and depressive symptoms. Very recently, Ouédraogo et al. [29] conducted a population-based study determining the prevalence of depressive symptoms in Burkina Faso for the first time.

In contrast to these previous studies, our work uses a large population-based sample in a mixed rural and small-town environment among a population of adults over 40 years of age. This allows us to examine the possible associations of depressive symptoms and CVD in older adults with less selection bias. It is of great importance to gather more information about the relationships between depressive symptoms and other morbidity in this SSA context, as it can provide novel ideas and solutions to better serve the healthcare needs of these populations.

METHODS

For peer review only

Sample

This population-based study was conducted in the Nouna Health and Demographic Surveillance System (HDSS) in north-western Burkina Faso, which is run by the Centre de Recherche en Santé de Nouna (CRSN) [30]. This HDSS is located in a rural region consisting of 58 villages centered around the town of Nouna. In 2015 there were about 107,000 individuals within the area, living in approximately 15,000 households. Of these individuals, approximately 18,000 were over the age of 40; we selected 4,000 of these individuals in a stratified two-stage cluster random sampling approach. Our first-stage sampling unit was at the level of villages and the seven sectors of Nouna town. Where there were fewer than 50 individuals aged over 40 (six villages in 2015 census), we included all of them in our sample. We then took the remaining sample in equal proportions from all remaining villages and sectors, selecting at random within village/sectors.

Data collection

Between May and July 2018 all sampled individuals were invited to participate at their homes. Consenting participants completed a questionnaire on socioeconomic and demographic characteristics, their physical, cognitive and mental health, and access to and usage of health care. Field workers were trained to administer the questionnaire, using French as the main language. Translations from French to the local dialect Djula were done within the training module and clearly communicated to all field workers verbally. Written Djula literacy is very limited in this setting, necessitating verbal rather than written translation.

Physical measurements such as weight, height and blood pressure were measured by study staff. A blood sample was collected to measure glycated hemoglobin (HbA1c), triglycerides, high density lipoprotein (HDL), and total cholesterol. To measure blood glucose levels, a point-of-care finger-prick test was carried out. All blood collection as well as finger-prick tests were conducted by trained and certified phlebotomists.

For peer review only

Outcome Variable

To measure depressive symptoms, the Patient Health Questionnaire depression module (PHQ-9) was used. The PHQ-9 is an instrument designed for primary care, either to make a probable diagnosis of major depressive disorder (MDD) or to continuously measure depressive symptoms with a score between 0 and 27 [31]. The scale consists of 9 items, with participants asked if they experienced a range of depressive symptoms in the past two weeks, including: (1) little interest or pleasure in doing things; (2) feeling down, depressed, or hopeless; (3) trouble falling or staying asleep, or sleeping too much; (4) feeling tired or having little energy; (5) poor appetite or overeating; (6) feeling bad about yourself; (7) trouble concentrating on things; (8) moving or speaking so slowly/rapidly that other people could have noticed; and (9) thoughts that you would be better off dead of or hurting yourself in some way. The response categories are: (0) not at all, (1) several days, (2) more than half of the days, (3) nearly every day.

Because PHQ-9 cut-points for diagnosing MDD in the sub-Saharan African context remain unclear [32], we use the PHQ-9 as a continuous score to reflect the amount of depressive symptoms an individual experienced in the past two weeks prior to the questioning. We emphasize that the score does not equal a diagnosis of depression. This linear specification avoids a binary categorization and allows subthreshold depression to be evaluated in our analyses. Subthreshold depression is characterized by clinically relevant symptomatology, and while less severe than MDD, its high prevalence can generate greater economic burden – in higher-income settings it is responsible for more doctor-patient consultations than MDD [33,34]. To account for potential interviewer effects, we calculated Z-scores for the PHQ-9 results within interviewer before analysis (Supplementary Figures 1 and 2).

Explanatory Variables

For peer review only

Based on the Harmonized Joint Scientific Statement (Harmonized) criteria [35], we defined someone as having the metabolic syndrome if they met at least three of five conditions: (1) body mass index (BMI) ≥ 30 kg/m^2; (2) mean systolic BP ≥ 130 mmHg or a mean diastolic BP ≥ 85 mmHg; (3) triglycerides ≥ 150 mg/dl; (4) HDL-cholesterol < 40 mg/dl in men, < 50 mg/dl in women; and (5) fasting blood glucose ≥ 100 mg/dl.

We also considered several individual chronic conditions as explanatory variables: having a positive screening result for hypertension grade II (i.e., BP levels of a mean systolic BP ≥ 140 mmHg or a mean diastolic BP ≥ 90 mmHg); having a positive screening result for prediabetes, defined as a fasting blood glucose value between 100 and 126 mg/dl and/or an HbA1c value between 5.7 and 6.5 %, based on the classification criteria of the American Diabetes Associations [36]; having a positive screening result for diabetes mellitus, defined as a fasting blood glucose value ≥ 126 mg/dl or an HbA1c value ≥ 6.5 %. Since roughly one-third of the glucose values had been non-fasting, thus random glucose values, we included observations with a random glucose value between 140 and 200 mg/dl in the definition of prediabetes, and observations with measurements of more than 200 mg/dl in the definition of diabetes.

Last, we used self-reported measures of chronic health conditions. These included whether the respondent reported ever being diagnosed with: elevated BP; elevated blood sugar; a heart attack; a stroke; or had ever experienced any of three stroke symptoms: sudden drooping of one side of the face; sudden numbness, weakness or dead feeling on one half of the body; sudden difficulty speaking or slurring of speech. We also created a ‘self-reported metabolic syndrome’ variable, defined as reporting any of the first four self-reported diagnoses.

The wealth index was constructed using polychoric principal components analysis (PCA) methods as described by Kolenikov and Angeles [37]. This method is similar to the Filmer and Pritchett [38] PCA method commonly used by Demographic and Household Surveys [39], but incorporates continuous variables and a lack of ownership into wealth index quantification. Variables in the final calculation included, among others: main source of water, toilet type,

For peer review only

number of bedrooms, number of cows, horses, and other animals, hectares of land, electricity, cell phone, and bank account.

Statistical Analysis

Baseline descriptive statistics were obtained and means calculated. Confidence intervals for binomial variables were calculated using the Clopper-Pearson intervals. Multivariable linear regression conducted to see how physical morbidity measures were associated with depressive symptoms. The standardized beta coefficients that are used to express the degree as to which morbidity variables are associated with the PHQ-9 score represent the percent value in which the probability of a yes/no event changes for every one standard deviation change in the PHQ-9 score. All models were adjusted for demographic and socioeconomic characteristics of the respondents, including age (in 5-year groups), sex, place of residence (rural/semi-rural), marital status (not-married/married), education (none/some, but none completed/primary completed or more), ethnicity (Dafin, Mossi, Bwama, other) and a wealth index. We standardized all coefficients so that they represented the standard deviations of change in the PHQ-9 score associated with a one-unit change in morbidity variable. All analyses were conducted using SPSS v25 (IBM, New York, USA)

Ethics

This study received ethics approval from the national health ethics committee in Ouagadougou (#2018-5-053), the CRSN institutional ethics committee in Nouna (#2018-04) and the ethics committee of the medical faculty of the Ruprecht-Karls-Universität Heidelberg (#S-120/2018).

Patients and Public Involvement

For peer review only

Patients and the public were not involved in the design, or conduct, or reporting, or dissemination plans of our research.

RESULTS

Summary statistics for the sample used in the present analysis is presented in Table 1. Of the 3,998 sampled individuals, 3,026 (75.7%) participants were found, consented and completed all questions from the PHQ-9 depression module and were included in the analysis. 1523 (50.3%) of the respondents were women, 1503 men. Women were older on average (mean: 55.5 years) than men (mean: 53.1 years). They were also more likely to be without formal schooling (90.7%) than men (78.1%), less likely to be married (55.3%, men: 84.7%) and had a higher mean BMI (22.5, men: 21.8). Positive screening results for hypertension, diabetes and hypertriglyceridemia all were equally distributed between both sexes. Elevated blood pressure was highly prevalent in the sample: 27.3% screened positive for grade II hypertension. The prevalence of elevated blood sugar in this population was relatively low (5.0% to 6.8%). 70.8% of the women had an HDL-cholesterol-level below the cut-point to fulfil the criteria for the metabolic syndrome, while only 45.7% of the men had an HDL-cholesterol below the cutoff.

For peer review only

Table 1: Sample characteristics

	**Total**	**Female**	**Male**
	***N* = 3026**	***n* = 1523**	***n* = 1503**
**Age group (y)**			
**40-44**	685 (22.6%)	300 (19.7%)	385 (25.6%)
**45-49**	579 (19.1%)	250 (16.4%)	329 (21.9%)
**50-54**	478 (15.8%)	252 (16.5%)	226 (15.0%)
**55-59**	393 (13.0%)	202 (13.3%)	191 (12.7%)
**60-64**	304 (10.0%)	178 (11.7%)	126 (8.4%)
**65-69**	249 (8.2%)	142 (9.3%)	107 (7.1%)
**70-74**	166 (5.5%)	94 (6.2%)	72 (4.8%)
**≥75**	172 (5.7%)	105 (6.9%)	67 (4.5%)
**Place of residence**			
**Nouna**	838 (27.7%)	452 (29.7%)	386 (25.7%)
**Village**	2188 (72.3%)	1071 (70.3%)	1117 (74.3%)
**Ethnicity**			
**Dafin**	1176 (38.9%)	575 (37.8%)	601 (40.0%)

**Bwama**	927 (30.6%)	490 (32.2%)	437 (29.1%)
**Mossi**	403 (13.3%)	199 (13.1%)	204 (13.6%)
**Other[a]**	520 (17.1%)	259 (16.9%)	261 (17.3%)
**Education**			
**No formal schooling**	2555 (84.4%)	1381 (90.7%)	1174 (78.1%)
**Some schooling, but none completed**	253 (8.4%)	79 (5.2%)	174 (11.6%)
**Primary schooling completed & more**	218 (7.2%)	63 (4.1%)	155 (10.3%)
**Currently married**	2115 (69.9%)	842 (55.3%)	1273 (84.7%)
**Body mass index[b]**			
**<18.5 kg/m²**	498 (16.8%)	268 (17.8%)	230 (15.6%)
**18.5-24.9 kg/m²**	1884 (63.4%)	875 (58.3%)	1009 (68.8%)
**25.0-29.9 kg/m²**	441 (14.8%)	249 (16.6%)	192 (13.1%)
**≥30.0 kg/m²**	148 (5.0%)	110 (7.3%)	38 (2.6%)
**Hypertension[c]**			
**Grade I**	1674 (56.3%)	841 (56.0%)	833 (56.7%)
**Grade II**	812 (27.3%)	433 (28.8%)	379 (25.8%)
**Diabetes mellitus**			
**Pre-diabetic[d]**	974 (45.4%)	530 (46.6%)	444 (44.1%)
**Diabetic[e]**	139 (6.8%)	84 (7.7%)	55 (5.7%)
**Triglycerides (≥150 mg/dl)[f]**	335 (11.6%)	177 (12.0%)	158 (11.2%)
**HDL-cholesterol[g] (<40 mg/dl in females, <50 mg/dl in males)[h]**	1366 (58.6%)	849 (70.8%)	517 (45.7%)
**Metabolic Syndrome[i]**	394 (15.6%)	260 (20.4%)	134 (10.7%)
**Self-reported conditions**			
**Elevated blood pressure**	507 (17.1%)	294 (19.8%)	213 (14.4%)
**Elevated blood sugar**	70 (2.3%)	32 (2.1%)	38 (2.6%)
**Heart disease**	166 (5.5%)	116 (7.7%)	50 (3.3%)
**Stroke**	40 (1.3%)	16 (1.1%)	24 (1.6%)
**Self-reported stroke symptoms**			
**Sudden drooping**	58 (1.9%)	33 (2.2%)	25 (1.7%)
**Sudden numbness**	240 (7.9%)	133 (8.7%)	107 (7.1%)
**Sudden slurring**	159 (5.3%)	88 (5.8%)	71 (4.7%)
**Self-reported metabolic syndrome[j]**	688 (23.3%)	396 (26.8%)	292 (19.9%)

[a]Peulh, Samo, Other. [b]55 missing values due to missing height/weight measurements. [c]55 missing values due to missing blood pressure measuremnts. [d]882 missing values due to missing HbA1c measurements and non-fasting results that were excluded. [e]982 missing values due to missing HbA1c measurements and non-fasting results that were excluded. [f]135 missing values due to missing Triglyceride measurements. [g]high-density-lipoprotein-cholesterol [h]696 missing values due to missing HDL-cholesterol measurements. [i]502 missing values due to missing height, weight, HDL-cholesterol, and Triglyceride measurements as well as non-fasting glucose measurements. [j]78 missing values.

Age patterns of depressive symptoms

As Figure 1 shows, the absolute mean values of the z-scored PHQ-9 scores rose continually with age in both men and women. Considering only men, the mean PHQ-9 value for the youngest group of respondents, the 40-44 years old, was -0.41 [95% confidence interval: -0.49 to -0.34]. This value rose constantly up to 0.88 [95% CI: 0.55 to 1.2] within the oldest age group of over 75-years-old. PHQ-9 scores were generally higher among women corresponding to higher levels of

depressive symptoms that were also positively correlated with age. The mean value for women in the youngest age group was -0,16 [95% CI: -0.25 to -0.07], rising to 0,80 [95% CI: 0.54 to 1.05] for women in the oldest group.

Age patterns of the metabolic syndrome, diabetes mellitus, and hypertension

Figure 2 shows that the prevalence of the metabolic syndrome increased in a statistically significant way with age for women – it increased from a mean of 12.0% [95% CI: 8.3% to 16.6%) within 40-44 year-olds to 28.2% [95% CI: 18.6% to 39.5%) within the 75+ age group. Surprisingly, the prevalence rate for men declined slightly with age, going from 8.2% [95% CI: 5.4% to 11.8%) to 5.7% [95% CI: 0.0% to 15.7%) – though this difference was not statistically significant. A small increase in the prevalence of a screening test consistent with prediabetes and diabetes prevalence was observed with increasing age, but this difference was not statistically significant. While an increasing prevalence with age could not be shown for diabetes mellitus, we did find a positive association between age and positively screened hypertension. Both men and women showed a rising prevalence of elevated blood pressure with increasing age.

Association of depressive symptoms with positive screening results for the metabolic syndrome, its components, hypertension and diabetes mellitus

The first panel of Table 2 and Figure 3 show the standardized coefficients from simple linear regressions of the z-scored PHQ-9 score on the metabolic syndrome, its components, hypertension and diabetes. In all models, higher age and being female were strongly associated with a higher PHQ-9 score. Having the metabolic syndrome, being positively screened for hypertension, prediabetes or diabetes was not associated with a higher PHQ-9 score in our sample. Neither was any of the single components of the metabolic syndrome.

Table 2: Standardized beta coefficients of linear regressions of the z-scored PHQ-9

	Standardized beta coefficient	95% confidence interval	p-value
**Metabolic syndrome**	-0.004	[-0.040, 0.033]	0.847

For peer review only

**Obesity**	0.007	[-0.028, 0.042]	0.680
**Hypertriglyceridemia**	-0.006	[-0.040, 0.029]	0.742
**Reduced HDL-cholesterol**	-0.027	[-0.067, 0.012]	0.177
**Hypertension grade I**	0.028	[-0.007, 0.062]	0.113
**FPG > 100**	-0.022	[-0.061, 0.019]	0.298
**Hypertension grade II**	0.014	[-0.020, 0.048]	0.427
**Prediabetes[a]**	0.005	[-0.034, 0.043]	0.819
**Diabetes[a]**	-0.001	[-0.040, 0.039]	0.972
**Self-reported conditions**			
**Hypertension**	0.032	[-0.003, 0.066]	0.071
**Diabetes**	0.004	[-0.029, 0.038]	0.809
**Heart disease**	0.081	[0.047, 0.114]	<0.001
**Stroke**	0.038	[0.005, 0.071]	0.024
**Self-reported stroke symptoms**			
**Sudden drooping**	0.090	[0.057, 0.123]	<0.001
**Sudden numbness**	0.136	[0.102, 0.168]	<0.001
**Sudden slurring**	0.144	[0.110, 0.176]	<0.001
**Self-reported metabolic syndrome**	0.070	[0.035, 0.104]	<0.001

Each standardized coefficient arises from a separate model controlling for age, sex, gender, place of residence, education, ethnicity and wealth quintile. The separate models can be found in Supplementary Materials (Supplementary Tables 1-17) [a]Using values without random plasma glucose.

Association of depressive symptoms with self-reported conditions and stroke symptoms

Figure 3 shows that having previously been diagnosed by a health care worker with elevated blood pressure or elevated blood sugar was not significantly associated with the PHQ-9 score, whereas the models did show a positive association for ever having been diagnosed with a stroke (standardized beta coefficient (hereafter ‘beta’) = 0.04 [95% confidence interval: 0.01 to 0.07]) or heart disease (beta = 0.08 [95% CI: 0.05 to 0.11]). An even stronger association could be shown for reporting to ever having had a sudden drooping of one side of the face (beta = 0.09 [95% CI: 0.06 to 0.12]), a sudden numbness weakness or dead feeling on one half of the body (beta = 0.14 [95% CI: 0.10 to 0.17]) and a sudden slurring of speech (beta = 0.14 [95% CI: 0.11 to 0.18]).

The ‘self-reported metabolic syndrome’ with one or more out of four self-reported health items present was associated positively with the PHQ-9 score (beta = 0.07 [95% CI: 0.04 to 0.10]). The results did not change substantially when running the regression models for the self-reported health items within the parts of the population who were screened positively for the respective

For peer review only

condition, e.g. the model with self-reported elevated blood pressure within the population who were measured as hypertensive (Supplementary Tables 18-20).

DISCUSSION

Chronic conditions and depressive symptoms

The findings suggest that positive screening results for the metabolic syndrome, hypertension and diabetes have no significant association with depressive symptoms in our sample. This situation is congruent with recent studies from SSA investigating the relationship between CVD and depression. Gelaye et al. [40] did not find an association between depression and the metabolic syndrome or diabetes in a sample of Ethiopian adults, Geldsetzer et al. [22] showed that there was no association between depression and hypertension, diabetes and obesity in a South African sample. In contrast to these results from SSA, relationships between CVD and depression have been widely reported in HIC [3-6,41]. In SSA, NCD including CVD are only recently emerging as a public health concern, while infectious diseases are still the most important cause of death [42]. People and health systems are more attuned to seeing and treating acute infections and maternal health problems than CVD, which might lead to a lower degree of understanding of what it means to have CVD – hence protecting the mental health of people in SSA. This hypothesis will need to be tested in future studies. Individuals with a higher socioeconomic status (SES) can afford higher health care costs than those with a lower SES, for whom it is a greater burden to pay for their health. Fiske et al. [17] found that unaffordable health care costs for individuals affected by CVD have a negative effect on their mental health.

CVD risk factors are asymptomatic, especially in their early stages, which might reduce the negative effects of CVD on mental health. Research in HIC has shown that there are biological factors like chronic inflammation, vascular endothelial dysfunction, neuroendocrine disequilibrium (particularly of the pituitary-adrenal axis) and increased platelet activation that can cause

For peer review only

depression in people with CVD and viceversa [43,44]. It remains unclear in the sub-Saharan African context if these biomedical processes are similarly important in driving an association between depressive symptoms and CVD; further research is needed to investigate this question. Multimorbidity might be another factor linking CVD and depression in HIC [45]. According to a recent study conducted in the same HDSS as this study, multimorbidity is highly prevalent in this sample [46]. Whether experiencing other NCDs in addition to CVD – for instance cancer, COPD, musculoskeletal disorders and other mental illnesses – has an effect on the relationship between depressive symptoms and CVD has not been examined and could be subject to further investigations. Use of antidepressants is linked to a higher risk of obesity, diabetes, and CVD [47] and could thus act as a confounding factor in studies in HIC investigating the association between depressive symptoms and cardiometabolic risk. In Burkina Faso the availability of specialized care is poor [48] and so is the availability of antidepressants, which makes it less likely for Burkinabé to experience the adverse outcomes of antidepressants.

For peer review only

Consequences of CVD

Interestingly, our findings suggest that having experienced the consequences of a stroke as well as having ever been diagnosed with a stroke or heart disease is associated with higher PHQ-9 results. These associations are in congruence with the situation in high-income countries, where especially ischemic events like heart attacks and strokes due to CVD are associated with depression [43]. Ischemic events may lead to physical dysfunction, impairing the ability to work in physical jobs that are important for the social and economic livelihood in a rural SSA environment. Social insecurity and disintegration resulting from these limitations are substantial risk factors to develop depressive symptoms. According to Schatz and Seeley [49], older adults in SSA strongly link their physical functionality to the concept of seeing themselves as independent individuals. With declines in physical functioning, their perceived independence deteriorates, leaving individuals as increasingly dependent and at risk of depression. Limited economic resources hinder the ability of older adults in rural areas to cope with stressors like disability after stroke, and also act to limit access to an already weak health system. This paucity

of diagnosis and treatment options also acts as part of a vicious cycle, as it may lead to substantial delays in accessing the health care system [50]. These barriers to mental health care lead to a lack of knowledge and awareness of an individual's own physical and mental health, making it much less likely to engage in protective behaviors such as regular physical activity, avoiding stimulants and drugs, engaging in stabile relationships or adjusting sleep rhythms and diet[51].

Sociodemographics

Women had a higher prevalence of depressive symptoms than men in our sample, showing that it will be especially important to target women in future interventions. With increasing age, the prevalence of depressive symptoms rose substantially for both men and women following a linear pattern, confirming similar findings from recent studies investigating depression in SSA [22,23]. Mechanisms that protect older populations in HIC include an increasing and positive influence of religiosity, gathered wisdom and life experience, higher economic resources, intensified social engagement and socio-emotional selectivity [17,18]. Examining whether these resilience mechanisms apply to the SSA context should be a concern for future researchers.

Implications

The findings suggest that improving care for measurable CVD conditions – metabolic syndrome, hypertension and diabetes – may have additional benefits for improving depressive symptoms. Even though they might not be directly associated with depressive symptoms, these conditions increase the likelihood of thromboembolic events which are associated with worse mental health. When depression is detected in its early stages, it might also be possible to prevent a part of CVD burden by engaging in preventive diagnostics and treatment in a targeted part of the population. While it is highly prevalent and likely an important correlate and basis for other chronic conditions and care success, depression has been woefully neglected in the global

For peer review only

health response. As diagnostic and therapeutic options exist, this gap should be addressed by policymakers as soon as possible.

Strengths and limitations

Our study had several strengths. It was large and population-based with a randomly selected sample, in contrast to the selective nature of almost all prior studies about depression in Burkina Faso, with the notable exception of Ouédraogo et al. [29]. The use of both questionnaire and biomarker testing allowed objective measurement of chronic conditions, using internationally standardized values allowing comparison to studies elsewhere. Furthermore, the PHQ-9 instrument is locally applicable, being widely used in primary care settings in SSA [23,52-54]. Nevertheless, its translation into the local language, particularly the collaborative manner in which it was done, makes it vulnerable to differences in expression during interviews. While we have adjusted for differential understanding by standardizing scores at the interviewer level, cultural differences in expressing psychological states like sadness may still be important. The cross-sectional design of the questionnaire and the one-time sample do not make it possible to draw causal interpretations from the findings or to confirm diagnoses of hypertension, diabetes and depression. Finally, our sample of adults aged 40 and above is not directly comparable to the typically older cut-offs for aging studies; however given the life expectancy of Burkinabé people (60.8 years [55]) we believe that the respondents represent old individuals within this setting. Clearly our findings only apply to over-40s, and associations may differ in younger individuals.

Conclusion

In conclusion, we find that having the metabolic syndrome, hypertension or diabetes was not associated with the results of the PHQ-9 score among a group of older adults in a rural, sub-Saharan African setting. Interestingly, an association could be found between depressive

For peer review only

symptoms and having experienced stroke symptoms as well as self-reported diagnose of stroke and heart disease. This might be due to the influence of disability after thromboembolic events on the psychological well-being of African adults seen in prior research. It may also be seen as a development foreshadowing an association of CVD and depression in this population, which cannot be seen yet because of lacking awareness, diagnosis and treatment options. Age was strongly associated with depressive symptoms, confirming the linear age pattern found in previous studies in SSA. Our results suggest that older African adults seem to lack resilience mechanisms protecting similar populations in HIC.

Acknowledgements

Funding for the CRSN Heidelberg Aging Study (CHAS) was provided by the Alexander von Humboldt Foundation through an Alexander von Humboldt Professor award to TB, which is funded by the German Federal Ministry of Education and Research. MDW acknowledges support from NIHR Newcastle Biomedical Research Centre. GH is supported by a fellowship from the Wellcome Trust and Royal Society (210479/Z/18/Z). The funders had no role in study design, data collection and analysis, decision to publish, or preparation of the manuscript.

Conflict of Interest

None reported.

Data availability

Data are not publicly available as consent was not given by participants for data to be shared openly. This is in part because entire age cohorts of some villages are included in the dataset, potentially allowing for deductive disclosure with sufficient local information. For this reason,

For peer review only

anonymized data is available from CHAS study data controllers only following signature of a data use agreement restricting onward transmission. Anyone wishing to replicate the analyses presented, or conduct further collaborative analyses using CHAS (which are welcomed and considered based on a letter of intent), should contact Dr Guy Harling (g.harling@ucl.ac.uk) in the first instance.

Contributors

TB and GH conceived and designed the overall CSRN CHAS study. CP, IK, JD, MW and MS contributed to the design of the CSRN CHAS household survey. BB, GH, AS, MB, LO and BC coordinated baseline data collection and preparation. BB conducted the analysis, and wrote, and revised the manuscript. CP and IK supervised the analysis, write up, and development of the manuscript. All authors substantively reviewed manuscripts, inputted into revisions, and approved the final manuscript.

REFERENCES

1 WHO. Depression and Other Common Mental Disorders: Global Health Estimates. 2017.

2 Vos T, Allen C, Arora M, Barber RM, Bhutta ZA, Brown A, et al. Global, regional, and national incidence, prevalence, and years lived with disability for 310 diseases and injuries, 1990–2015: a systematic analysis for the Global Burden of Disease Study 2015. The Lancet. 2016;388:1545-602.

3 Repousi N, Masana MF, Sanchez-Niubo A, Haro JM, Tyrovolas S. Depression and metabolic syndrome in the older population: A review of evidence. J Affect Disord. 2018;237:56-64.

4 Ghanei Gheshlagh R, Parizad N, Sayehmiri K. The Relationship Between Depression and Metabolic Syndrome: Systematic Review and Meta-Analysis Study. Iran Red Crescent Med J. 2016;18:e26523-e.

5 Marazziti D, Rutigliano G, Baroni S, Landi P, Dell'Osso L. Metabolic syndrome and major depression. CNS Spectrums. 2014;19:293-304.

For peer review only

6 Vaccarino V, McClure C, Johnson BD, Sheps DS, Bittner V, Rutledge T, et al. Depression, the metabolic syndrome and cardiovascular risk. Psychosom Med. 2008;70:40-8.

7 Stein DJ, Benjet C, Gureje O, Lund C, Scott KM, Poznyak V, et al. Integrating mental health with other non-communicable diseases. BMJ. 2019;364:l295.

8 Bao AM, Meynen G, Swaab DF. The stress system in depression and neurodegeneration: Focus on the human hypothalamus. Brain Research Reviews. 2008;57:531-53.

9 Björntorp P. Do stress reactions cause abdominal obesity and comorbidities? Obesity Reviews. 2001;2:73-86.

10 Carney RM, Freedland KE, Veith RC. Depression, the Autonomic Nervous System, and Coronary Heart Disease. Psychosomatic Medicine. 2005;67:S29-S33.

11 Golden SH. A review of the evidence for a neuroendocrine link between stress, depression and diabetes mellitus. Curr Diabetes Rev. 2007;3:252-9.

12 Carnethon MR, Biggs ML, Barzilay JI, Smith NL, Vaccarino V, Bertoni AG, et al. Longitudinal Association Between Depressive Symptoms and Incident Type 2 Diabetes Mellitus in Older Adults: The Cardiovascular Health Study. Archives of Internal Medicine. 2007;167:802-7.

13 Ford DE, Erlinger TP. Depression and C-Reactive Protein in US Adults: Data From the Third National Health and Nutrition Examination Survey. Archives of Internal Medicine. 2004;164:1010-4.

14 Pan A, Sun Q, Okereke OI, Rexrode KM, Hu FB. Depression and Risk of Stroke Morbidity and Mortality: A Meta-analysis and Systematic Review. JAMA. 2011;306:1241-9.

15 Skala JA, Freedland KE, Carney RM. Coronary Heart Disease and Depression: A Review of Recent Mechanistic Research. The Canadian Journal of Psychiatry. 2006;51:738-45.

16 Huang C-Q, Dong B-R, Lu Z-C, Yue J-R, Liu Q-X. Chronic diseases and risk for depression in old age: A meta-analysis of published literature. Ageing Research Reviews. 2010;9:131-41.

17 Fiske A, Wetherell JL, Gatz M. Depression in older adults. Annu Rev Clin Psychol. 2009;5:363-89.

18 Blazer DG, Hybels CF. Origins of depression in later life. Psychol Med. 2005;35:1241-52.

19 Robinson RG, Jorge RE. Post-Stroke Depression: A Review. American Journal of Psychiatry. 2015;173:221-31.

20 Ayerbe L, Ayis S, Wolfe CDA, Rudd AG. Natural history, predictors and outcomes of depression after stroke: systematic review and meta-analysis. British Journal of Psychiatry. 2013;202:14-21.

For peer review only

21 Lichtman Judith H, Froelicher Erika S, Blumenthal James A, Carney Robert M, Doering Lynn V, Frasure-Smith N, et al. Depression as a Risk Factor for Poor Prognosis Among Patients With Acute Coronary Syndrome: Systematic Review and Recommendations. Circulation. 2014;129:1350-69.

22 Geldsetzer P, Vaikath M, Wagner R, Rohr JK, Montana L, Gomez-Olive FX, et al. Depressive Symptoms and Their Relation to Age and Chronic Diseases Among Middle-Aged and Older Adults in Rural South Africa. J Gerontol A Biol Sci Med Sci. 2019;74:957-63.

23 Kohler IV, Payne CF, Bandawe C, Kohler HP. The Demography of Mental Health Among Mature Adults in a Low-Income, High-HIV-Prevalence Context. Demography. 2017;54:1529-58.

24 Blazer DG. Depression in late life: review and commentary. J Gerontol A Biol Sci Med Sci. 2003;58:249-65.

25 Thapa SB, Martinez P, Clausen T. Depression and its correlates in South Africa and Ghana among people aged 50 and above: Findings from the WHO Study on global AGEing and adult health. Journal of psychiatry. 2014;17:1000167.

26 Yameogo NV, Samadoulougou A, Kagambega LJ, Yameogo AA, Ilboudo E, Millogo G, et al. [Sleep disorders, anxiety and depressive symptoms and cardiovascular risk among black African hypertensive: cross-sectional study of 414 hypertensive followed as outpatients at the University Hospital of Ouagadougou (Burkina Faso)]. Pan Afr Med J. 2015;21:115.

27 Napon C, Kaboré A, Kaboré J. Post stroke depression in Burkina Faso. The Pan African medical journal. 2012;13:3-.

28 Duthe G, Rossier C, Bonnet D, Soura AB, Corker J. Mental health and urban living in sub-Saharan Africa: major depressive episodes among the urban poor in Ouagadougou, Burkina Faso. Popul Health Metr. 2016;14:18.

29 Ouédraogo A, Ouango JG, Karfo K, Goumbri P, Nanéma D, Sawadogo B. Prévalence des troubles mentaux en population générale au Burkina Faso. L'Encéphale. 2019;45:367-70.

30 Sie A, Louis VR, Gbangou A, Muller O, Niamba L, Stieglbauer G, et al. The Health and Demographic Surveillance System (HDSS) in Nouna, Burkina Faso, 1993-2007. Glob Health Action. 2010;3.

31 Kroenke K, Spitzer RL, Williams JB, Lowe B. The Patient Health Questionnaire Somatic, Anxiety, and Depressive Symptom Scales: a systematic review. Gen Hosp Psychiatry. 2010;32:345-59.

32 Sweetland AC, Belkin GS, Verdeli H. Measuring depression and anxiety in sub-saharan Africa. Depression and anxiety. 2014;31:223-32.

For peer review only

33 Cuijpers P, Vogelzangs N, Twisk J, Kleiboer A, Li J, Penninx BW. Differential mortality rates in major and subthreshold depression: meta-analysis of studies that measured both. British Journal of Psychiatry. 2013;202:22-7.

34 Johnson J, Weissman MM, Klerman GL. Service Utilization and Social Morbidity Associated With Depressive Symptoms in the Community. JAMA. 1992;267:1478-83.

35 Alberti KG, Eckel RH, Grundy SM, Zimmet PZ, Cleeman JI, Donato KA, et al. Harmonizing the metabolic syndrome: a joint interim statement of the International Diabetes Federation Task Force on Epidemiology and Prevention; National Heart, Lung, and Blood Institute; American Heart Association; World Heart Federation; International Atherosclerosis Society; and International Association for the Study of Obesity. Circulation. 2009;120:1640-5.

36 American Diabetes A. 2. Classification and Diagnosis of Diabetes: Standards of Medical Care in Diabetes-2018. Diabetes Care. 2018;41:S13-S27.

37 Kolenikov S, Angeles G. SOCIOECONOMIC STATUS MEASUREMENT WITH DISCRETE PROXY VARIABLES: IS PRINCIPAL COMPONENT ANALYSIS A RELIABLE ANSWER? Review of Income and Wealth. 2009;55:128-65.

38 Filmer D, Pritchett LH. Estimating Wealth Effects Without Expenditure Data—Or Tears: An Application To Educational Enrollments In States Of India*. Demography. 2001;38:115-32.

39 Rutstein SO, Johnson K. The DHS wealth index. Calverton, Maryland, USA: ORC Macro, 2004.

40 Gelaye B, Williams MA, Lemma S, Berhane Y, Fann JR, Vander Stoep A, et al. Major depressive disorder and cardiometabolic disease risk among sub-Saharan African adults. Diabetes & metabolic syndrome. 2015;9:183-91.

41 Pan A, Keum N, Okereke OI, Sun Q, Kivimaki M, Rubin RR, et al. Bidirectional association between depression and metabolic syndrome: a systematic review and meta-analysis of epidemiological studies. Diabetes care. 2012;35:1171-80.

42 Institute for Health Metrics and Evaluation (IHME). GBD Compare Data Visualization. Seattle, WA: IHME, University of Washington. Available from http://vizhub.healthdata.org/gbd-compare. (Accessed 9th of November 2019). 2018.

43 Goldston K, Baillie AJ. Depression and coronary heart disease: A review of the epidemiological evidence, explanatory mechanisms and management approaches. Clinical Psychology Review. 2008;28:288-306.

For peer review only

44 Krishnan KR, Delong M, Kraemer H, Carney R, Spiegel D, Gordon C, et al. Comorbidity of depression with other medical diseases in the elderly. Biol Psychiatry. 2002;52:559-88.

45 Søgaard M, Skjøth F, Kjældgaard JN, Larsen TB, Hjortshøj SP, Riahi S. Atrial fibrillation in patients with severe mental disorders and the risk of stroke, fatal thromboembolic events and bleeding: a nationwide cohort study. BMJ open. 2017;7:e018209-e.

46 Odland ML, Payne C, Witham M, Siedner M, Bärnighausen T, Bountogo M, et al. The epidemiology of multimorbidity in conditions of extreme poverty: a population-based study of older adults in rural Burkina Faso. 2019.

47 Correll CU, Detraux J, De Lepeleire J, De Hert M. Effects of antipsychotics, antidepressants and mood stabilizers on risk for physical diseases in people with schizophrenia, depression and bipolar disorder. World Psychiatry. 2015;14:119-36.

48 Maiga DD, Eaton J. A survey of the mental healthcare systems in five Francophone countries in West Africa: Bénin, Burkina Faso, Côte d'Ivoire, Niger and Togo. International Psychiatry. 2014;11:69-72.

49 Schatz E, Seeley J. Gender, ageing and carework in East and Southern Africa: A review. Glob Public Health. 2015;10:1185-200.

50 Collins KA, Westra HA, Dozois DJ, Burns DD. Gaps in accessing treatment for anxiety and depression: challenges for the delivery of care. Clin Psychol Rev. 2004;24:583-616.

51 Sarris J, O'Neil A, Coulson CE, Schweitzer I, Berk M. Lifestyle medicine for depression. BMC psychiatry. 2014;14:107-.

52 Motlhatlhedi K, Setlhare V, Ganiyu AB, Firth JA. Association between depression in carers and malnutrition in children aged 6 months to 5 years. African Journal of Primary Health Care & Family Medicine. 2017;9:1-6.

53 L'Akoa R M, Noubiap JJ, Fang Y, Ntone FE, Kuaban C. Prevalence and correlates of depressive symptoms in HIV-positive patients: a cross-sectional study among newly diagnosed patients in Yaounde, Cameroon. BMC Psychiatry. 2013;13:228.

54 Bindt C, Appiah-Poku J, Te Bonle M, Schoppen S, Feldt T, Barkmann C, et al. Antepartum depression and anxiety associated with disability in African women: cross-sectional results from the CDS study in Ghana and Cote d'Ivoire. PLoS One. 2012;7:e48396.

55 United Nations Development Program. Human Development Indices and Indicators: 2018 Statistical Update. Country notes - Burkina Faso. Accessed 4th November 2019. 2018.

For peer review only

FIGURES

Figure 1: Mean PHQ-9 scores (z-scored within interviewer) and 95% confidence intervals. Underlying data can be found in Supplementary Materials (Supplementary Table 21)

Figure 2: Prevalence of a positive screening result for the metabolic syndrome, pre-diabetes, diabetes and hypertension; 95% confidence intervals. Underlying data can be found in Supplementary Materials (Supplementary Tables 22 – 25)

Figure 3: Forest plot of associations between non-communicable conditions and PHQ-9 z-scores.

For peer review only

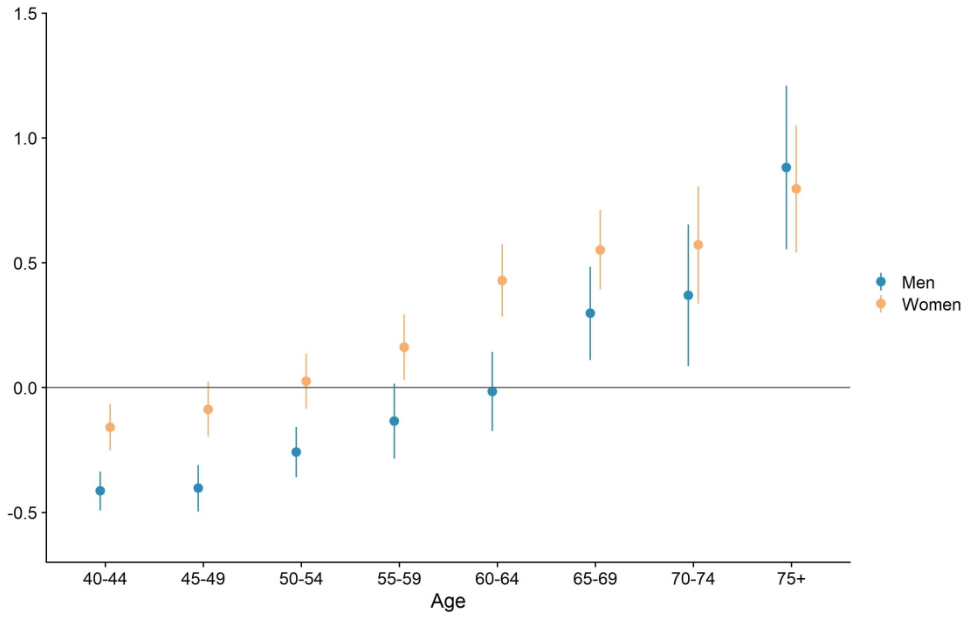

246x152mm (300 x 300 DPI)

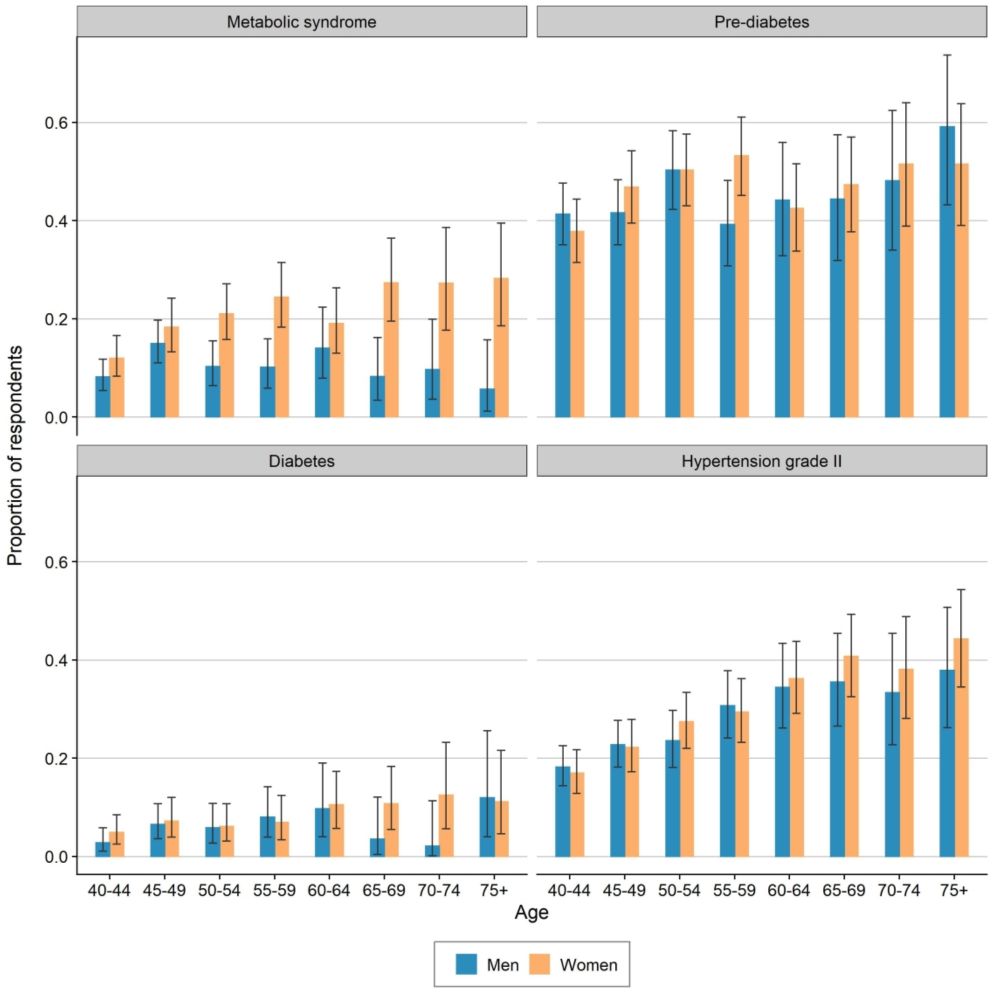

254x254mm (300 x 300 DPI)

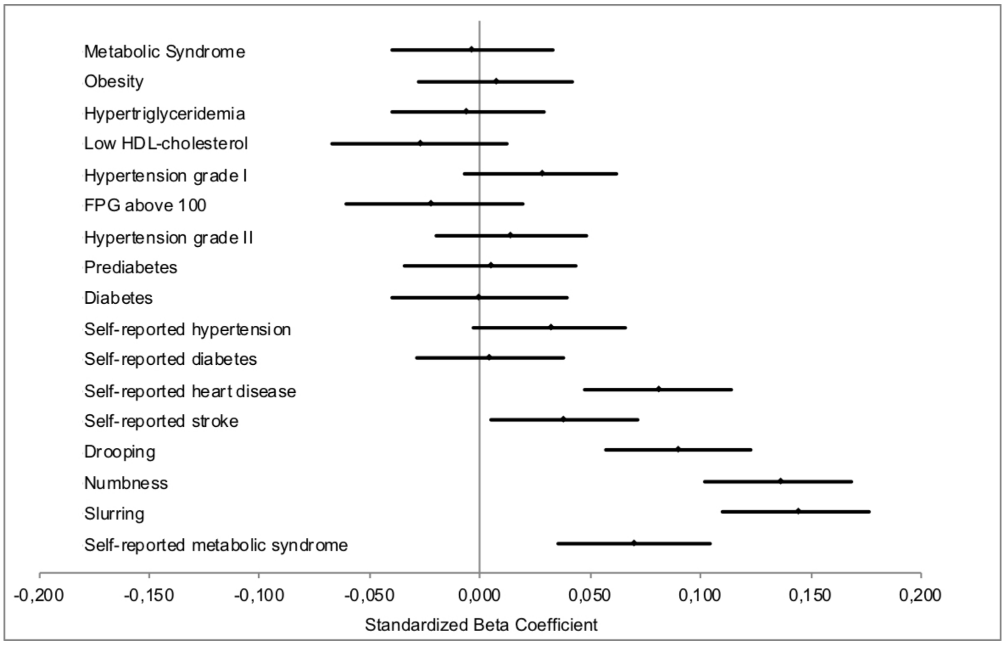

199x127mm (150 x 150 DPI)

SUPPLEMENTARY MATERIAL

Supplementary Figure 1: This figure shows the distribution of the mean raw PHQ-9 values by single interviewers. The differences between interviewers' PHQ-9 values were interpreted as an interviewer effect during analyses. We decided to standardize the results on the interviewer level and add them up afterwards.

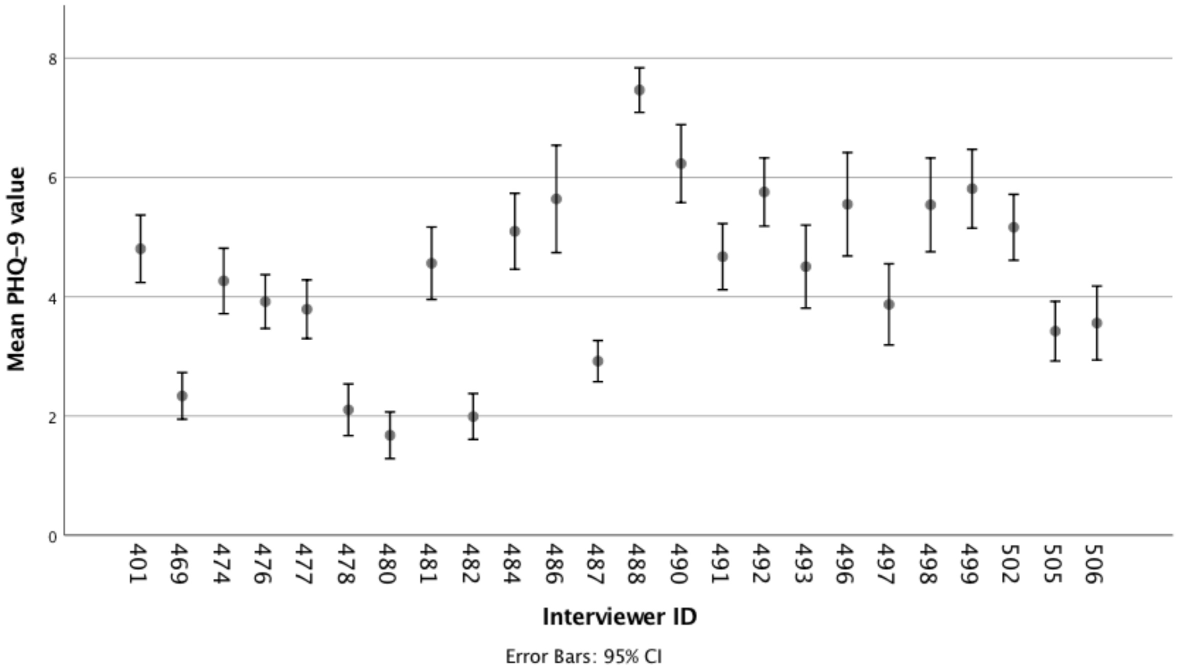

Supplementary Figure 2: The figure shows the standardized PHQ-9 values of one of the interviewers (401) and the normal distribution curve. We z-scored the PHQ-9 results for this interviewer with the standardization function in SPSS. Each value was calculated by dividing the difference between the value and the mean by the standard deviation. This was done for all interviewers separately. Afterwards, all values were included in the outcome variable of the z-scored PHQ-9 score.

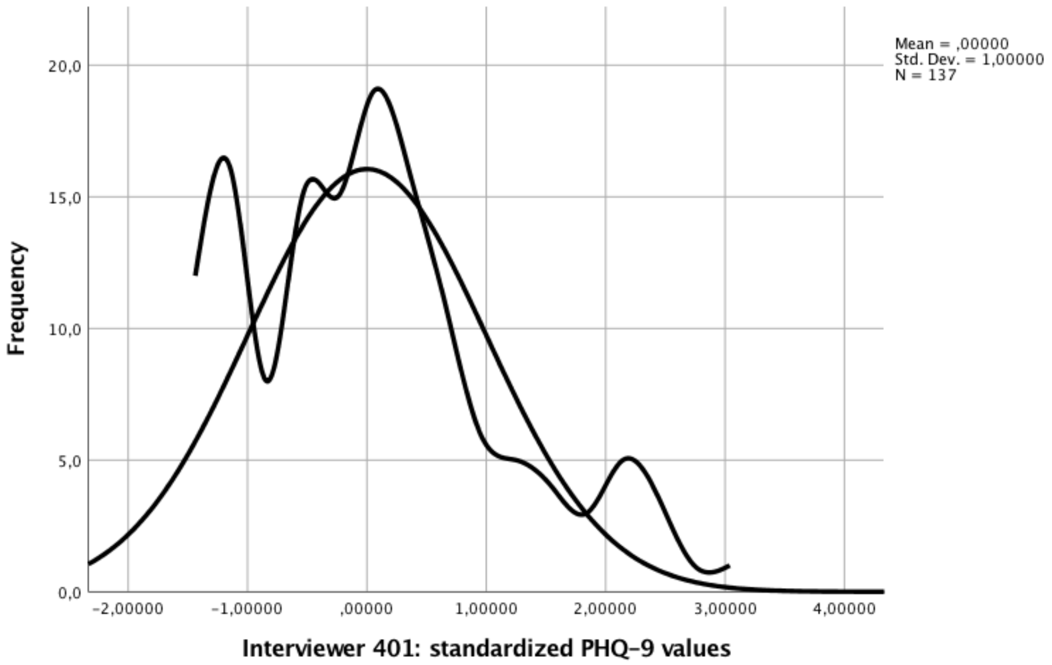

Supplementary Table 1: Linear regression model 1 (Metabolic syndrome)

Dependent variable: Z-scored PHQ-9

		Model 1 (n=2524)			
		beta	CI low	CI high	p
Age	40-44	-	-	-	-
	45-49	.007	-.037	.050	.773
	50-54	.058	.014	.098	.010
	55-59	.098	.053	.136	<.001
	60-64	.144	.101	.184	<.001
	65-69	.182	.139	.221	<.001
	70-74	.147	.104	.183	<.001
	75+	.251	.215	.298	<.001
Sex (ref: male)		.115	.074	.151	<.001
Living in the countryside		-.052	-.088	-.011	.013
Currently married		-.020	-.065	.022	.340
Education	None				
	Some	.018	-.020	.054	.360
	Primary & more	-.049	-.088	-.009	.016
Ethnicity	Dafin				
	Bwama	.017	-.022	.053	.430
	Mossi	.007	-.028	.040	.734
	Other	.022	-.024	.082	.280
Wealth quintile	1				
	2	-.030	-.074	.016	.208
	3	-.051	-.096	-.003	.038
	4	-.058	-.103	-.009	.019
	5	-.083	-.135	-.033	.001
**Metabolic syndrome**		-.004	-.040	.033	.847

For peer review only

Supplementary Table 2: Linear regression model 2
Abdominal Obesity

Dependent variable: Z-scored PHQ-9

		Model 2 (n=2970)			
		beta	CI low	CI high	p
Age	40-44	-	-	-	-
	45-49	.017	-.024	.058	.406
	50-54	.059	.019	.099	.004
	55-59	.101	.062	.140	<.001
	60-64	.143	.104	.181	<.001
	65-69	.189	.150	.226	<.001
	70-74	.164	.126	.200	<.001
	75+	.239	.200	.275	<.001
Sex (ref: male)		.118	.081	.153	<.001
Living in the countryside		-.047	-.078	-.008	.016
Currently married		-.028	-.072	.010	.143
Education	None				
	Some	.019	-.016	.053	.288
	Primary & more	-.033	-.071	.003	.072
Ethnicity	Dafin				
	Bwama	.021	-.017	.055	.302
	Mossi	-.001	-.033	.031	.944
	Other	.026	-.015	.080	.180
Wealth quintile	1				
	2	-.020	-.062	.023	.372
	3	-.056	-.100	-.012	.013
	4	-.066	-.110	-.021	.004
	5	-.087	-.135	-.040	<.001
**Abdominal Obesity**		.007	-.028	.042	.680

For peer review only

Supplementary Table 3: Linear regression model 3 (Hypertriglyceridemia)

Dependent variable: Z-scored PHQ-9

		Model 3 (n=2890)			
		beta	CI low	CI high	p
Age	40-44	-	-	-	-
	45-49	.013	-.028	.055	.531
	50-54	.064	.022	.104	.002
	55-59	.098	.057	.137	<.001
	60-64	.144	.103	.182	<.001
	65-69	.186	.144	.221	<.001
	70-74	.159	.120	.194	<.001
	75+	.243	.203	.280	<.001
Sex (ref: male)		.115	.078	.150	<.001
Living in the countryside		-.052	-.083	-.012	.009
Currently married		-.029	-.073	.010	.141
Education	None				
	Some	.017	-.018	.052	.330
	Primary & more	-.041	-.079	-.004	.029
Ethnicity	Dafin				
	Bwama	.015	-.023	.050	.456
	Mossi	.001	-.031	.034	.938
	Other	.025	-.017	.080	.200
Wealth quintile	1				
	2	-.024	-.067	.020	.285
	3	-.054	-.099	-.010	.017
	4	-.069	-.113	-.023	.003
	5	-.083	-.132	-.036	.001
**Hypertriglyceridemia**		-.006	-.040	.029	.742

For peer review only

Supplementary Table 4: Linear regression model 4
(Reduced HDL-cholesterol)

Dependent variable: Z-scored PHQ-9

		Model 4 (n=2330)			
		beta	CI low	CI high	p
Age	40-44	-	-	-	-
	45-49	.019	-.028	.065	.425
	50-54	.061	.015	.106	.009
	55-59	.110	.063	.151	<.001
	60-64	.154	.107	.193	<.001
	65-69	.185	.141	.227	<.001
	70-74	.170	.124	.206	<.001
	75+	.234	.196	.283	<.001
Sex (ref: male)		.134	.091	.174	<.001
Living in the countryside		-.028	-.078	.016	.193
Currently married		-.031	-.079	.013	.158
Education	None				
	Some	.004	-.034	.042	.843
	Primary & more	-.035	-.084	.006	.092
Ethnicity	Dafin				
	Bwama	.007	-.032	.045	.745
	Mossi	-.016	-.051	.022	.437
	Other	.010	-.048	.080	.620
Wealth quintile	1				
	2	-.035	-.080	.012	.147
	3	-.059	-.106	-.010	.017
	4	-.075	-.121	-.025	.003
	5	-.072	-.133	-.026	.004
**Reduced HDL-cholesterol**		-.027	-.067	.012	.177

For peer review only

Supplementary Table 5: Linear regression model 5 (Hypertension grade I)

Dependent variable: Z-scored PHQ-9

		Model 5 (n=2970)			
		beta	CI low	CI high	p
Age	40-44	-	-	-	-
	45-49	.016	-.025	.057	.441
	50-54	.057	.017	.097	.006
	55-59	.098	.058	.137	<.001
	60-64	.139	.099	.177	<.001
	65-69	.185	.145	.222	<.001
	70-74	.161	.123	.197	<.001
	75+	.235	.196	.271	<.001
Sex (ref: male)		.119	.082	.154	<.001
Living in the countryside		-.046	-.077	-.007	.020
Currently married		-.028	-.072	.010	.141
Education	None				
	Some	.020	-.015	.054	.257
	Primary & more	-.034	-.071	.002	.067
Ethnicity	Dafin				
	Bwama	.020	-.018	.054	.326
	Mossi	.000	-.032	.032	.982
	Other	.027	-.014	.081	.164
Wealth quintile	1				
	2	-.019	-.062	.024	.379
	3	-.055	-.099	-.011	.014
	4	-.066	-.110	-.022	.004
	5	-.087	-.134	-.040	<.001
**Hypertension grade I**		.028	-.007	.062	.113

For peer review only

Supplementary Table 6: Linear regression model 6
(Fasting Plasma Glucose > 100 mg/dl)

Dependent variable: Z-scored PHQ-9

		Model 6 (n=2038)			
		beta	CI low	CI high	p
Age	40-44	-	-	-	-
	45-49	.002	-.046	.049	.939
	50-54	.049	.000	.093	.048
	55-59	.102	.053	.144	<.001
	60-64	.143	.096	.188	<.001
	65-69	.192	.144	.236	<.001
	70-74	.125	.078	.164	<.001
	75+	.246	.206	.297	<.001
Sex (ref: male)		.114	.069	.153	<.001
Living in the countryside		-.077	-.114	-.028	.001
Currently married		-.016	-.065	.031	.488
Education	None				
	Some	.024	-.018	.068	.257
	Primary & more	-.066	-.107	-.021	.004
Ethnicity	Dafin				
	Bwama	-.003	-.045	.040	.904
	Mossi	.010	-.028	.045	.663
	Other	.030	-.019	.094	.190
Wealth quintile	1				
	2	-.028	-.078	.024	.295
	3	-.058	-.110	-.004	.035
	4	-.077	-.126	-.021	.006
	5	-.101	-.154	-.042	.001
**Fasting Plasma Glucose > 100 mg/dl**		-.022	-.061	.019	.298

For peer review only

Supplementary Table 7: Linear regression model 7
(Hypertension grade II)

Dependent variable: Z-scored PHQ-9

		Model 7 (n=2970)			
		beta	CI low	CI high	p
Age	40-44	-	-	-	-
	45-49	.017	-.024	.058	.422
	50-54	.059	.018	.099	.004
	55-59	.100	.060	.139	<.001
	60-64	.141	.102	.179	<.001
	65-69	.188	.148	.224	<.001
	70-74	.163	.125	.199	<.001
	75+	.237	.197	.273	<.001
Sex (ref: male)		.118	.082	.154	<.001
Living in the countryside		-.047	-.078	-.008	.016
Currently married		-.028	-.072	.010	.143
Education	None				
	Some	.019	-.015	.054	.274
	Primary & more	-.033	-.070	.003	.072
Ethnicity	Dafin				
	Bwama	.020	-.017	.054	.309
	Mossi	-.001	-.033	.031	.946
	Other	.026	-.015	.081	.176
Wealth quintile	1				
	2	-.019	-.062	.024	.386
	3	-.055	-.099	-.011	.014
	4	-.066	-.109	-.021	.004
	5	-.086	-.133	-.039	<.001
**Hypertension grade II**		.014	-.020	.048	.427

For peer review only

Supplementary Table 8: Linear regression model 8 (Pre-diabetes)

Dependent variable: Z-scored PHQ-9

		Model 8 (n=2143)			
		beta	CI low	CI high	p
Age	40-44	-	-	-	-
	45-49	.005	-.042	.052	.838
	50-54	.050	.002	.093	.040
	55-59	.099	.050	.139	<.001
	60-64	.142	.096	.187	<.001
	65-69	.204	.155	.244	<.001
	70-74	.128	.082	.166	<.001
	75+	.240	.198	.287	<.001
Sex (ref: male)		.110	.066	.148	<.001
Living in the countryside		-.070	-.106	-.022	.003
Currently married		-.018	-.065	.028	.432
Education	None				
	Some	.026	-.015	.067	.216
	Primary & more	-.071	-.110	-.026	.001
Ethnicity	Dafin				
	Bwama	.000	-.041	.041	.999
	Mossi	.007	-.030	.042	.756
	Other	.038	-.008	.102	.097
Wealth quintile	1				
	2	-.024	-.073	.027	.368
	3	-.056	-.106	-.003	.040
	4	-.067	-.115	-.013	.014
	5	-.097	-.148	-.039	.001
**Pre-diabetes**		.005	-.034	.043	.819

For peer review only

Supplementary Table 9: Linear regression model 9 (Diabetes)

Dependent variable: Z-scored PHQ-9

		Model 9 (n=2043)			
		beta	CI low	CI high	p
Age	40-44	-	-	-	-
	45-49	.001	-.047	.048	.983
	50-54	.048	.000	.092	.051
	55-59	.104	.055	.146	<.001
	60-64	.143	.096	.188	<.001
	65-69	.191	.143	.235	<.001
	70-74	.123	.077	.163	<.001
	75+	.246	.204	.295	<.001
Sex (ref: male)		.112	.066	.150	<.001
Living in the countryside		-.081	-.118	-.031	.001
Currently married		-.017	-.065	.030	.474
Education	None				
	Some	.031	-.011	.075	.147
	Primary & more	-.066	-.105	-.020	.004
Ethnicity	Dafin				
	Bwama	-.010	-.051	.033	.672
	Mossi	.008	-.030	.043	.735
	Other	.031	-.018	.095	.179
Wealth quintile	1				
	2	-.033	-.082	.019	.227
	3	-.069	-.120	-.014	.013
	4	-.083	-.132	-.027	.003
	5	-.105	-.158	-.045	<.001
**Diabetes**		-.001	-.040	.039	.972

For peer review only

Supplementary Table 10: Linear regression model 10
(Self-reported hypertension)

Dependent variable: Z-scored PHQ-9

		Model 10 (n=2972)			
		beta	CI low	CI high	p
Age	40-44	-	-	-	-
	45-49	.016	-.025	.057	.434
	50-54	.056	.015	.095	.007
	55-59	.093	.053	.131	<.001
	60-64	.143	.103	.180	<.001
	65-69	.179	.140	.217	<.001
	70-74	.152	.115	.190	<.001
	75+	.235	.196	.271	<.001
Sex (ref: male)		.119	.083	.155	<.001
Living in the countryside		-.044	-.074	-.005	.027
Currently married		-.036	-.080	.003	.066
Education	None				
	Some	.017	-.017	.051	.329
	Primary & more	-.027	-.062	.010	.152
Ethnicity	Dafin				
	Bwama	.017	-.021	.051	.404
	Mossi	.000	-.032	.032	.999
	Other	.026	-.014	.081	.172
Wealth quintile	1				
	2	-.015	-.058	.029	.512
	3	-.050	-.093	-.005	.028
	4	-.060	-.104	-.015	.009
	5	-.087	-.133	-.039	<.001
**Self-reported hypertension**		.032	-.003	.066	.071

For peer review only

Supplementary Table 11: Linear regression model 11
(Self-reported diabetes)

Dependent variable: Z-scored PHQ-9

		Model 11 (n=2991)			
		beta	CI low	CI high	p
Age	40-44	-	-	-	-
	45-49	.013	-.028	.053	.541
	50-54	.058	.018	.097	.005
	55-59	.098	.057	.135	<.001
	60-64	.144	.104	.181	<.001
	65-69	.182	.143	.219	<.001
	70-74	.154	.117	.190	<.001
	75+	.233	.194	.269	<.001
Sex (ref: male)		.123	.086	.158	<.001
Living in the countryside		-.044	-.074	-.005	.024
Currently married		-.036	-.080	.002	.062
Education	None				
	Some	.020	-.014	.054	.250
	Primary & more	-.024	-.060	.012	.195
Ethnicity	Dafin				
	Bwama	.016	-.022	.050	.438
	Mossi	-.001	-.032	.031	.965
	Other	.023	-.018	.076	.230
Wealth quintile	1				
	2	-.015	-.058	.028	.500
	3	-.054	-.097	-.010	.016
	4	-.065	-.108	-.020	.004
	5	-.085	-.130	-.037	<.001
**Self-reported diabetes**		.004	-.029	.038	.809

For peer review only

Supplementary Table 12: Linear regression model 12
(Self-reported heart disease)

Dependent variable: Z-scored PHQ-9

		Model 12 (n=3001)			
		beta	CI low	CI high	p
Age	40-44	-	-	-	-
	45-49	.012	-.029	.052	.567
	50-54	.056	.016	.095	.006
	55-59	.094	.054	.132	<.001
	60-64	.143	.105	.181	<.001
	65-69	.181	.143	.219	<.001
	70-74	.161	.124	.197	<.001
	75+	.236	.197	.272	<.001
Sex (ref: male)		.112	.076	.148	<.001
Living in the countryside		-.047	-.077	-.008	.016
Currently married		-.029	-.072	.009	.128
Education	None				
	Some	.018	-.017	.052	.314
	Primary & more	-.026	-.062	.010	.159
Ethnicity	Dafin				
	Bwama	.016	-.021	.051	.410
	Mossi	-.002	-.033	.030	.931
	Other	.021	-.021	.074	.272
Wealth quintile	1				
	2	-.021	-.063	.022	.338
	3	-.058	-.102	-.015	.009
	4	-.071	-.115	-.027	.002
	5	-.092	-.138	-.045	<.001
**Self-reported heart disease**		.081	.047	.114	<.001

For peer review only

Supplementary Table 13: Linear regression model 13
(Self-reported stroke)

Dependent variable: Z-scored PHQ-9

		Model 13 (n=3026)			
		beta	CI low	CI high	p
Age	40-44	-	-	-	-
	45-49	.015	-.025	.056	.455
	50-54	.058	.018	.097	.004
	55-59	.094	.055	.133	<.001
	60-64	.143	.104	.181	<.001
	65-69	.185	.147	.222	<.001
	70-74	.162	.125	.198	<.001
	75+	.236	.197	.272	<.001
Sex (ref: male)		.122	.086	.157	<.001
Living in the countryside		-.049	-.079	-.010	.012
Currently married		-.031	-.074	.008	.110
Education	None				
	Some	.019	-.015	.053	.282
	Primary & more	-.027	-.062	.009	.147
Ethnicity	Dafin				
	Bwama	.019	-.018	.053	.344
	Mossi	-.001	-.032	.031	.960
	Other	.023	-.018	.076	.224
Wealth quintile	1				
	2	-.018	-.061	.024	.401
	3	-.055	-.098	-.011	.014
	4	-.065	-.109	-.021	.004
	5	-.087	-.134	-.040	<.001
**Self-reported stroke**		.038	.005	.071	.024

For peer review only

Supplementary Table 14: Linear regression model 14
(Sudden drooping)

Dependent variable: Z-scored PHQ-9

		Model 14 (n=3026)			
		beta	CI low	CI high	p
Age	40-44	-	-	-	-
	45-49	.013	-.027	.054	.519
	50-54	.054	.014	.093	.008
	55-59	.094	.055	.132	<.001
	60-64	.137	.099	.175	<.001
	65-69	.183	.144	.220	<.001
	70-74	.157	.120	.193	<.001
	75+	.235	.196	.271	<.001
Sex (ref: male)		.120	.084	.155	<.001
Living in the countryside		-.049	-.078	-.010	.012
Currently married		-.031	-.075	.006	.100
Education	None				
	Some	.019	-.015	.053	.284
	Primary & more	-.027	-.062	.009	.147
Ethnicity	Dafin				
	Bwama	.015	-.021	.049	.438
	Mossi	-.001	-.033	.030	.947
	Other	.022	-.019	.075	.241
Wealth quintile	1				
	2	-.017	-.059	.026	.446
	3	-.056	-.099	-.013	.012
	4	-.066	-.109	-.022	.003
	5	-.087	-.133	-.041	<.001
**Sudden drooping**		.090	.057	.123	<.001

For peer review only

Supplementary Table 15: Linear regression model 15
(Sudden numbness)

Dependent variable: Z-scored PHQ-9

		Model 15 (n=3026)			
		beta	CI low	CI high	p
Age	40-44	-	-	-	-
	45-49	.017	-.024	.057	.419
	50-54	.059	.019	.098	.004
	55-59	.095	.056	.133	<.001
	60-64	.135	.096	.172	<.001
	65-69	.178	.140	.215	<.001
	70-74	.155	.118	.190	<.001
	75+	.227	.189	.263	<.001
Sex (ref: male)		.117	.081	.151	<.001
Living in the countryside		-.051	-.080	-.012	.008
Currently married		-.034	-.078	.003	.071
Education	None				
	Some	.016	-.018	.049	.366
	Primary & more	-.026	-.062	.009	.147
Ethnicity	Dafin				
	Bwama	.017	-.019	.051	.380
	Mossi	.001	-.031	.032	.965
	Other	.020	-.022	.072	.291
Wealth quintile	1				
	2	-.019	-.061	.023	.378
	3	-.061	-.104	-.017	.006
	4	-.072	-.115	-.028	.001
	5	-.097	-.143	-.051	<.001
**Sudden numbness**		.136	.102	.168	<.001

For peer review only

Supplementary Table 16: Linear regression model 16 (Sudden slurring)

Dependent variable: Z-scored PHQ-9

		Model 16 (n=3026)			
		beta	CI low	CI high	p
Age	40-44	-	-	-	-
	45-49	.016	-.025	.055	.448
	50-54	.056	.017	.096	.005
	55-59	.093	.054	.131	<.001
	60-64	.134	.096	.172	<.001
	65-69	.175	.137	.212	<.001
	70-74	.155	.118	.190	<.001
	75+	.227	.189	.263	<.001
Sex (ref: male)		.117	.081	.151	<.001
Living in the countryside		-.052	-.081	-.013	.007
Currently married		-.037	-.081	.000	.050
Education	None				
	Some	.019	-.015	.053	.264
	Primary & more	-.029	-.064	.007	.113
Ethnicity	Dafin				
	Bwama	.017	-.019	.051	.379
	Mossi	.001	-.030	.032	.954
	Other	.017	-.026	.068	.374
Wealth quintile	1				
	2	-.022	-.064	.020	.312
	3	-.060	-.103	-.017	.006
	4	-.072	-.116	-.029	.001
	5	-.091	-.136	-.044	<.001
**Sudden slurring**		.144	.110	.176	<.001

For peer review only

Supplementary Table 17: Linear regression model 17
(Self-reported metabolic syndrome)

Dependent variable: Z-scored PHQ-9

		Model 17 (n=2948)			
		beta	CI low	CI high	p
Age	40-44	-	-	-	-
	45-49	.014	-.027	.055	.497
	50-54	.055	.014	.094	.008
	55-59	.092	.052	.130	<.001
	60-64	.141	.101	.178	<.001
	65-69	.174	.135	.212	<.001
	70-74	.151	.114	.188	<.001
	75+	.230	.190	.266	<.001
Sex (ref: male)		.117	.080	.152	<.001
Living in the countryside		-.039	-.070	.000	.049
Currently married		-.039	-.083	-.001	.045
Education	None				
	Some	.018	-.017	.052	.314
	Primary & more	-.029	-.064	.008	.124
Ethnicity	Dafin				
	Bwama	.015	-.022	.049	.460
	Mossi	-.001	-.033	.031	.956
	Other	.024	-.017	.079	.201
Wealth quintile	1				
	2	-.016	-.059	.027	.459
	3	-.051	-.095	-.007	.024
	4	-.066	-.110	-.021	.004
	5	-.091	-.137	-.042	<.001
**Self-reported metabolic syndrome**		.070	.035	.104	<.001

For peer review only

Supplementary Table 18: Descriptive statistics of the self-reported diagnosis of hypertension and diabetes within the whole sample

	Total	Female	Male
**Self-reported diagnosis of hypertension**	*n* = 3007<br>507 (17.1%)	*n* = 1513<br>294 (19.8%)	*n* = 1494<br>213 (14.4%)
**Self-reported diagnosis of diabetes**	*n* = 2993<br>70 (2.3%)	*n* = 1505<br>32 (2.1%)	*n* = 1488<br>38 (2.6%)

Supplementary Table 19: Descriptive statistics of the self-reported diagnosis of hypertension and diabetes within the affected* part of the sample

	Total	Female	Male
**Self-reported diagnosis of hypertension**	*n* = 1674<br>346 (21.0%)	*n* = 841<br>203 (24.6%)	*n* = 832<br>143 (17.3%)
**Self-reported diagnosis of diabetes**	*n* = 143<br>20 (14.0%)	*n* = 85<br>11 (13.0%)	*n* = 58<br>9 (15.5%)

*In case of Hypertension: Systolic BP >130 mmHg or Diastolic BP >80 mmHg
Diabetes: Fasting Glucose >126 mg/dl, random glucose >200 mg/dl or HbA1c >6.5%

Supplementary Table 20: Measuring the effect of a diagnosis of hypertension/diabetes given by a health care worker among hypertensive/diabetic part of the population.
Given in the table are the standardized coefficients of multiple linear regression of the z-scored PHQ-9 score on self-reported diagnosis of hypertension within the hypertensive population[a] and diagnosis of diabetes within the diabetic population[b].

	Standardized beta coefficient	p-value
**Ever been diagnosed with hypertension**	0.033	0.061
**Ever been diagnosed with diabetes**	-0.025	0.762

All variables used in separate models controlling for age, sex, gender, place of residence, education, ethnicity and wealth quintile.
[a] Systolic BP >130 mmHg or Diastolic BP >80 mmHg
[b] Fasting Glucose >126 mg/dl, random glucose >200 mg/dl or HbA1c >6.5%

**Supplementary Table 21:** Distribution of the z-scored PHQ-9 values					
		PHQ-9 value	CI lower	CI upper	2 Std. Dev.
m	40-44	-0,414	-0,493	-0,336	0,078
w	40-44	-0,160	-0,252	-0,067	0,092
m	45-49	-0,403	-0,496	-0,311	0,092
w	45-49	-0,087	-0,196	0,022	0,109
m	50-54	-0,259	-0,359	-0,158	0,100
w	50-54	0,025	-0,084	0,135	0,110
m	55-59	-0,135	-0,285	0,015	0,150
w	55-59	0,162	0,030	0,293	0,131
m	60-64	-0,016	-0,175	0,142	0,159
w	60-64	0,428	0,284	0,572	0,144
m	65-69	0,297	0,111	0,484	0,186
w	65-69	0,552	0,393	0,710	0,159
m	70-74	0,369	0,085	0,653	0,284
w	70-74	0,571	0,337	0,806	0,235
m	75+	0,881	0,553	1,210	0,328
w	75+	0,795	0,541	1,050	0,254

For peer review only

**Supplementary Table 22:** Prevalence of the metabolic syndrome				
		Proportion	CI lower	CI upper
m	40-44	0,082	0,054	0,118
w	40-44	0,120	0,083	0,166
m	45-49	0,150	0,110	0,197
w	45-49	0,183	0,133	0,242
m	50-54	0,103	0,064	0,155
w	50-54	0,210	0,158	0,271
m	55-59	0,101	0,059	0,159
w	55-59	0,244	0,183	0,315
m	60-64	0,140	0,079	0,224
w	60-64	0,190	0,130	0,263
m	65-69	0,082	0,034	0,162
w	65-69	0,274	0,195	0,364
m	70-74	0,097	0,036	0,199
w	70-74	0,273	0,177	0,386
m	75+	0,057	0,012	0,157
w	75+	0,282	0,186	0,395

For peer review only

Supplementary Table 23: Prevalence of a positive screening result for pre-diabetes

		Proportion	CI lower	CI upper
m	40-44	0,413	0,351	0,476
w	40-44	0,378	0,315	0,444
m	45-49	0,416	0,351	0,483
w	45-49	0,468	0,395	0,542
m	50-54	0,503	0,423	0,583
w	50-54	0,503	0,430	0,576
m	55-59	0,392	0,308	0,482
w	55-59	0,532	0,451	0,611
m	60-64	0,442	0,328	0,559
w	60-64	0,425	0,338	0,516
m	65-69	0,444	0,319	0,575
w	65-69	0,473	0,377	0,570
m	70-74	0,481	0,340	0,624
w	70-74	0,515	0,389	0,640
m	75+	0,591	0,432	0,737
w	75+	0,515	0,390	0,638

For peer review only

**Supplementary Table 24:** Prevalence of a positive screening result for diabetes mellitus				
		Proportion	CI lower	CI upper
m	40-44	0,028	0,011	0,058
w	40-44	0,049	0,025	0,085
m	45-49	0,065	0,036	0,107
w	45-49	0,072	0,039	0,120
m	50-54	0,058	0,027	0,108
w	50-54	0,061	0,031	0,107
m	55-59	0,080	0,039	0,142
w	55-59	0,069	0,034	0,124
m	60-64	0,097	0,040	0,190
w	60-64	0,105	0,057	0,173
m	65-69	0,035	0,004	0,121
w	65-69	0,107	0,055	0,183
m	70-74	0,021	0,001	0,113
w	70-74	0,125	0,056	0,232
m	75+	0,119	0,040	0,256
w	75+	0,111	0,046	0,216

For peer review only

**Supplementary Table 25:** Prevalence of a positive screening result for hypertension gr. II				
		Proportion	CI lower	CI upper
m	40-44	0,182	0,144	0,225
w	40-44	0,169	0,128	0,217
m	45-49	0,227	0,182	0,277
w	45-49	0,222	0,172	0,279
m	50-54	0,235	0,181	0,297
w	50-54	0,274	0,220	0,334
m	55-59	0,306	0,241	0,378
w	55-59	0,294	0,232	0,362
m	60-64	0,344	0,261	0,434
w	60-64	0,362	0,291	0,438
m	65-69	0,355	0,265	0,454
w	65-69	0,407	0,325	0,493
m	70-74	0,333	0,227	0,454
w	70-74	0,380	0,281	0,488
m	75+	0,379	0,262	0,507
w	75+	0,442	0,345	0,543

For peer review only

BMJ Open

Depressive symptoms and cardiovascular disease: a population-based study of older adults in rural Burkina Faso

Journal:	*BMJ Open*
Manuscript ID	bmjopen-2020-038199.R1
Article Type:	Original research
Date Submitted by the Author:	02-May-2020
Complete List of Authors:	Brinkmann, Ben; Heidelberg University, Heidelberg Institute of Global Health<br>Payne, Collin F.; • School of Demography, The Australian National University, Canberra, Australia, School of Demography<br>Kohler, Iliana ; University of Pennsylvania, Population Studies Center (PSC) and Department of Sociology<br>Harling, Guy; University College London, Institute for Global Health; Harvard Center for Population and Development Studies<br>Davies, Justine ; University of Birmingham, Institute of Applied Health Research<br>Witham, Miles; AGE Research Group, NIHR Newcastle Biomedical Research Centre, Newcastle University; Newcastle Upon Tyne Hospitals NHS Foundation Trust<br>Siedner, Mark; Harvard Medical School, Department of Medicine, Massachusetts General Hospital<br>Sie, Ali; Centre de Recherche en Sante de Nouna<br>Bountogo, Mamadou; Centre de Recherche en Sante de Nouna<br>Ouermi, Lucienne; Centre de Recherche en Sante de Nouna<br>Coulibaly, Boubacar; Centre de Recherche en Sante de Nouna<br>Bärnighausen, Till ; Heidelberg University, Heidelberg Institute of Global Health; Africa Health Research Institute
**Primary Subject Heading**:	Public health
Secondary Subject Heading:	Public health, Mental health, Cardiovascular medicine, Epidemiology, Geriatric medicine
Keywords:	Depression & mood disorders < PSYCHIATRY, Hypertension < CARDIOLOGY, STROKE MEDICINE, PUBLIC HEALTH, EPIDEMIOLOGY, MENTAL HEALTH

SCHOLARONE™
Manuscripts

BMJ

I, the Submitting Author has the right to grant and does grant on behalf of all authors of the Work (as defined in the below author licence), an exclusive licence and/or a non-exclusive licence for contributions from authors who are: i) UK Crown employees; ii) where BMJ has agreed a CC-BY licence shall apply, and/or iii) in accordance with the terms applicable for US Federal Government officers or employees acting as part of their official duties; on a worldwide, perpetual, irrevocable, royalty-free basis to BMJ Publishing Group Ltd ("BMJ") its licensees and where the relevant Journal is co-owned by BMJ to the co-owners of the Journal, to publish the Work in this journal and any other BMJ products and to exploit all rights, as set out in our licence.

The Submitting Author accepts and understands that any supply made under these terms is made by BMJ to the Submitting Author unless you are acting as an employee on behalf of your employer or a postgraduate student of an affiliated institution which is paying any applicable article publishing charge ("APC") for Open Access articles. Where the Submitting Author wishes to make the Work available on an Open Access basis (and intends to pay the relevant APC), the terms of reuse of such Open Access shall be governed by a Creative Commons licence – details of these licences and which Creative Commons licence will apply to this Work are set out in our licence referred to above.

Other than as permitted in any relevant BMJ Author's Self Archiving Policies, I confirm this Work has not been accepted for publication elsewhere, is not being considered for publication elsewhere and does not duplicate material already published. I confirm all authors consent to publication of this Work and authorise the granting of this licence.

For peer review only

Title:

Depressive symptoms and cardiovascular disease: a population-based study of older adults in rural Burkina Faso

Corresponding Author:

Ben Brinkmann

Im Neuenheimer Feld 130.3

69120 Heidelberg

Germany

ben.brinkmann@stud.uni-heidelberg.de

+49 1573 6194470

Authors:

Ben Brinkmann[1], mail: ben.brinkmann@stud.uni-heidelberg.de

Collin Payne, PhD[2], mail: collin.payne@anu.edu.au

Iliana V. Kohler, PhD[3], mail: iliana@pop.upenn.edu

Guy Harling, ScD[4,5,6,7], mail: g.harling@ucl.ac.uk

Justine I. Davies[8], mail: j.davies.6@bham.ac.uk

Miles D. Witham[9], mail: Miles.Witham@newcastle.ac.uk

Mark J. Siedner[10], mail: MSIEDNER@mgh.harvard.edu

Ali Sié, MD, PhD[11], mail: sieali.crsn@fasonet.bf

Mamadou Bountogo, MD, MSc[11], mail: drbountogo@yahoo.fr

Lucienne Ouermi, MD[11], mail: ouermil@yahoo.fr

Boubacar Coulibaly, MD[11], mail: boubacar@fasonet.bf

Till Bärnighausen, MD, ScD[1,4], mail: till.baernighausen@uni-heidelberg.de

Affiliations:

[1] Heidelberg Institute of Global Health, Heidelberg University, Germany.

[2] School of Demography, Australian National University, Canberra, Australian Capital Territory, Australia.

For peer review only

[3] Population Studies Center (PSC) and Department of Sociology, University of Pennsylvania, Philadelphia, PA, USA.

[4] Harvard Center for Population and Development Studies, Cambridge, MA, USA.

[5] Institute for Global Health, University College London, London, UK.

[6] Africa Health Research Institute, KwaZulu-Natal, South Africa

[7] MRC/WITS Rural Public Health and Health Transitions Research Unit (Agincourt), Faculty of Health Sciences, University of the Witwatersrand, Johannesburg, South Africa

[8] Institute of Applied Health Research, University of Birmingham, Birmingham, UK

[9] AGE Research Group, NIHR Newcastle Biomedical Research Centre, Newcastle University and Newcastle upon Tyne Hospitals NHS Trust, Newcastle, UK

[10] Department of Medicine, Massachusetts General Hospital, Harvard Medical School, Boston, MA, USA

[11] Centre de Recherche en Santé de Nouna, Nouna, Burkina Faso.

Word count:

4,610

For peer review only

ABSTRACT

Objectives: To contribute to the current understanding of depressive disorders in sub-Saharan African (SSA) countries by examining the association of depressive symptoms with cardiovascular and cardiometabolic conditions in a population-based study of middle-aged and older adults in rural Burkina Faso.

Setting: This study was conducted in the Nouna Health and Demographic Surveillance System (HDSS) in north-western Burkina Faso, in a mixed rural and small-town environment. The data were obtained between May and July 2018.

Participants: Consenting adults over 40 years of age (n=3,026).

Primary and secondary outcome measures: Depressive symptoms were assessed using the Patient Health Questionnaire depression module (PHQ-9). Chronic cardiometabolic conditions were assessed via a lipid panel and HbA1c measures from serum, alongside anthropometry and blood pressure measurements and a self-reported questionnaire. Multivariable linear regression was used to test the relationship between depressive symptoms and cardiovascular/cardiometabolic conditions, after controlling for sociodemographic factors.

Results: Depressive symptoms were not associated with the metabolic syndrome (standardized beta coefficient = 0.00 [95% confidence interval (CI): -0.04 to 0.03]), hypertension (beta = 0.01 [95% CI: -0.02 to 0.05]), diabetes mellitus (beta = 0.00 [95% CI: -0.04 to 0.04]) and past diagnosis of elevated blood pressure or blood sugar. Prior stroke diagnosis (beta = 0.04 [95% CI: 0.01 to 0.07]) or heart disease (beta = 0.08 [95% CI: 0.05 to 0.11]) was positively associated with the standardized PHQ-9 score, as were self-reported stroke symptoms.

Conclusion: Objectively measured cardiometabolic conditions had no significant association with depressive symptoms in an older, poor, rural SSA population, in contrast to observations in high income

For peer review only

countries (HIC). However, consequences of cardiovascular disease such as stroke and heart attack were associated with depressive symptoms in older adults in Burkina Faso.

STRENGTHS TO THIS STUDY

- This study was large and population-based with a randomly selected sample, in contrast to the selective nature of prior studies about depression in Burkina Faso.
- The use of biomarker testing for cardiovascular health and the PHQ-9 depression screening instrument has been validated in primary care settings in SSA and provides internationally comparable data.

LIMITATIONS TO THIS STUDY

- The oral translation of the study questionnaire into local languages makes it vulnerable to differences in expression during interviews.
- The cross-sectional design of the questionnaire limits our ability to determine temporal ordering of and causal links between cardiovascular and mental health events.
- We were not able to clinically confirm diagnoses of hypertension, diabetes, metabolic syndrome and depression in study participants.

For peer review only

KEY QUESTIONS

What is already known?

Depressive symptoms are positively associated with cardiovascular disease risk factors in high income countries (HIC). Previous literature from SSA shows that these associations may not generalize to lower income countries (LIC).

What are the new findings?

Cardiovascular disease risk factors and conditions like the metabolic syndrome, hypertension, diabetes mellitus and self-reported elevated blood pressure and sugar were not associated with depressive symptoms in a rural, poor Burkina Faso environment. Prior diagnosis of stroke or heart disease as well as self-reported stroke symptoms were positively associated with depressive symptoms.

What do the new findings imply?

Depressive symptoms in older adults with cardiovascular disease might be prevented when detected in early stages, as well as cardiovascular conditions in adults with depressive symptoms. As accessible diagnostic and therapeutic options exist for both cardiovascular disease and depressive symptoms, this gap should be addressed by policymakers as soon as possible.

For peer review only

INTRODUCTION

The decline in communicable diseases and the ageing of populations worldwide, including low-income countries, means that cardiovascular disease (CVD) is now the leading cause of mortality worldwide, accounting for an estimated 31% of all deaths [1]. The leading cause of morbidity in the Global Burden of Disease study 2017, however, was depressive disorders [2].

In high-income contexts, CVD risk factors as summarized by the metabolic syndrome – obesity, dyslipidemia, hypertension and diabetes mellitus – are bidirectionally associated with depressive disorders [3-6]. People with depression are more likely to smoke tobacco, eat an unhealthy diet, be physically inactive and use alcohol to excess, which makes them more vulnerable to develop the metabolic syndrome [7]. Elevated activity of the hypothalamus-pituitary adrenal (HPA) axis caused by chronic stress in people with depression increases the risk for obesity, dyslipidemia and diabetes [8,9]. Alterations in the autonomic nervous system found in people with depression [10] contribute to hypertension and increased insulin resistance. Other related factors adding to the hypothesis of a shared pathophysiology are over-activated inflammatory pathways, oxidative stress, endothelial dysfunction and hormone disequilibrium [5,11-13].

CVDs such as ischemic heart disease and cerebrovascular stroke are associated with subsequent depression. While depression increases the risk of heart attack and stroke [14,15], both are also associated with higher levels of later depression [16-18]. Post-stroke depression might be a result of the brain damage from the stroke but can also be a psychological reaction to the stroke illness and to its disabling consequences. Risk factors for depressive symptoms after stroke include stroke severity, disability after stroke, cognitive impairment, lack of social support and genetic predisposition [19,20]. Furthermore, depression worsens the outcomes of stroke [20] and myocardial infarction [21], increasing mortality and worsening long-term functional results.

In recent years, a small but growing body of evidence from sub-Saharan Africa (SSA) suggests that associations between CVD, diabetes and depression seen in HIC may not generalize to lower-income settings [22,23]. In addition, it has been found that the age pattern for depressive

For peer review only

disorders differs substantially from high-income countries. Depression prevalence in high-income countries peaks around age 60 and declines in the following decades, resulting in an inverted U-shape [24]. In contrast, in low-income contexts the prevalence of depressive symptoms appears to increase consistently with age [23,25].

In this paper, we seek to contribute to the current understanding of depressive disorders in sub-Saharan African countries by using newly collected data from Burkina Faso. Specifically, we aim to examine the potential associations of depressive symptoms with the metabolic syndrome, hypertension, diabetes mellitus and self-reported chronic conditions and stroke symptoms as well as the age pattern of depressive symptoms. Depression and mental disorders more generally are understudied in Burkina Faso and the evidence about the relationships with CVDs is inconclusive. Two recent studies examined the prevalence of depressive symptoms within pre-selected populations, Yameogo et al. [26] focused on hypertensive outpatients and Napon et al. [27] investigated depressive symptoms among people who had a stroke. Duthé et al. [28] worked with a sample of urban adults in the capital city of Ouagadougou, finding an association between broadly defined self-reported chronic conditions and depressive symptoms. Very recently, Ouédraogo et al. [29] conducted a population-based study determining the prevalence of depressive symptoms in Burkina Faso for the first time.

In contrast to these previous studies, our work uses a large population-based sample in a mixed rural and small-town environment among a population of adults over 40 years of age. This allows us to examine the possible associations of depressive symptoms and CVD in older adults with less selection bias. It is of great importance to gather more information about the relationships between depressive symptoms and other diseases' morbidity in this SSA context, as it can provide novel ideas and solutions to better serve the healthcare needs of these populations.

METHODS

For peer review only

Sample

This population-based study was conducted in the Nouna Health and Demographic Surveillance System (HDSS) in north-western Burkina Faso, which is run by the Centre de Recherche en Santé de Nouna (CRSN) [30]. This HDSS is located in a rural region consisting of 58 villages centered around the town of Nouna. In 2015 there were about 107,000 individuals within the area, living in approximately 15,000 households. Of these individuals, approximately 18,000 were over the age of 40; we selected 3,998 of these individuals in a stratified two-stage cluster random sampling approach. Our first-stage sampling unit was at the level of villages and the seven sectors of Nouna town. Where there were fewer than 50 individuals aged over 40 (six villages in the 2015 census), we included all of them in our sample. We then took the remaining sample in equal proportions from all remaining villages and sectors, selecting at random within village/sectors.

Data collection

Between May and July 2018 all sampled individuals were invited to participate at their homes. Consenting participants completed a questionnaire on socioeconomic and demographic characteristics, their physical, cognitive and mental health, and access to and usage of health care. Field workers were trained to administer the questionnaire, using French as the main language. Translations from French to the local dialect Djula were done within the training module and clearly communicated to all field workers verbally. Written Djula literacy is very limited in this setting, necessitating verbal rather than written translation.

Indicators of physical health such as weight, height and blood pressure were measured by study staff. Blood pressure was measured using Omron Series 7 portable blood pressure machines (Omron Healthcare, Kyoto, Japan). After 15 minutes of rest, three measurements were taken in the left arm of the seated respondents with 5 minutes between each measurement. The mean of the second and third measurement was calculated and used in analyses. A blood sample was

For peer review only

collected to measure glycated hemoglobin (HbA1c), triglycerides, high density lipoprotein (HDL), and total cholesterol. To measure blood glucose levels, a point-of-care finger-prick test was carried out. The participants were asked when they last consumed food or beverages to determine whether the glucose values were fasting or non-fasting. All blood collection as well as finger-prick tests were conducted by trained and certified phlebotomists.

Outcome Variable

To measure depressive symptoms, the Patient Health Questionnaire depression module (PHQ-9) was used. The PHQ-9 is an instrument designed for primary care, either to make a probable diagnosis of major depressive disorder (MDD) or to continuously measure depressive symptoms with a score between 0 and 27 [31]. Higher scores represent more severe depressive symptoms. The scale consists of 9 items, with participants asked if they experienced a range of depressive symptoms in the past two weeks, including: (1) little interest or pleasure in doing things; (2) feeling down, depressed, or hopeless; (3) trouble falling or staying asleep, or sleeping too much; (4) feeling tired or having little energy; (5) poor appetite or overeating; (6) feeling bad about yourself; (7) trouble concentrating on things; (8) moving or speaking so slowly/rapidly that other people could have noticed; and (9) thoughts that you would be better off dead of or hurting yourself in some way. The response categories are: (0) not at all, (1) several days, (2) more than half of the days, (3) nearly every day.

Because PHQ-9 cut-points for diagnosing MDD in the sub-Saharan African context remain unclear [32], we use the PHQ-9 as a continuous score to reflect the amount of depressive symptoms an individual experienced in the past two weeks prior to the questioning. We emphasize that the score does not equal a diagnosis of depression. This linear specification avoids a binary categorization and allows subthreshold depression to be evaluated in our analyses. Subthreshold depression is characterized by clinically relevant symptomatology, and while less severe than MDD, its high prevalence can generate greater economic burden – in higher-income settings it is responsible for more doctor-patient consultations than MDD [33,34].

For peer review only

To account for potential interviewer effects, we calculated Z-scores for the PHQ-9 results within interviewer before analysis (Supplementary Figures 1 and 2).

Explanatory Variables

Based on the Harmonized Joint Scientific Statement (Harmonized) criteria [35], we defined someone as having the metabolic syndrome if they met at least three of five conditions: (1) body mass index (BMI) ≥ 30 kg/m^2; (2) mean systolic BP ≥ 130 mmHg or a mean diastolic BP ≥ 85 mmHg; (3) triglycerides ≥ 150 mg/dl; (4) HDL-cholesterol < 40 mg/dl in men, < 50 mg/dl in women; and (5) fasting blood glucose ≥ 100 mg/dl.

We also considered several individual chronic conditions as explanatory variables: having a positive screening result for hypertension grade II (i.e., BP levels of a mean systolic BP ≥ 140 mmHg or a mean diastolic BP ≥ 90 mmHg) [36]; having a positive screening result for prediabetes, defined as a fasting blood glucose value between 100 and 126 mg/dl and/or an HbA1c value between 5.7 and 6.5 %, based on the classification criteria of the American Diabetes Associations [37]; having a positive screening result for diabetes mellitus, defined as a fasting blood glucose value ≥ 126 mg/dl or an HbA1c value ≥ 6.5 %.

In the remainder of this article, we define 'hypertension' as a positive screening result for hypertension, rather than the clinically diagnosed condition (i.e., by a medically trained health care provider) according to country specific requirements. The same accounts for 'diabetes mellitus' and the 'metabolic syndrome'

Last, we used self-reported measures of chronic health conditions. These included whether the respondent reported ever being diagnosed with: elevated BP; elevated blood sugar; a heart attack; a stroke; or had ever experienced any of three stroke symptoms: sudden drooping of one side of the face; sudden numbness, weakness or dead feeling on one half of the body; sudden difficulty speaking or slurring of speech. The stroke symptoms were selected according to the

FAST (“Face”, “Arm”, “Speech”, “Time”) system, which was created by the Brain Attack Coalition based on the Cincinnati Pre-Hospital Stroke Scale in 1999 [38]. We also created a ‘self-reported metabolic syndrome’ variable, defined as reporting any of the first four self-reported diagnoses.

The wealth index was constructed using polychoric principal components analysis (PCA) methods as described by Kolenikov and Angeles [39]. This method is similar to the Filmer and Pritchett [40] PCA method commonly used by Demographic and Household Surveys [41], but incorporates continuous variables and a lack of ownership into wealth index quantification. Variables in the final calculation included, among others: main source of water, toilet type, number of bedrooms, number of cows, horses, and other animals, hectares of land, electricity, cell phone, and bank account.

Statistical Analysis

Baseline descriptive statistics were obtained and means calculated. Confidence intervals for binomial variables were calculated using the Clopper-Pearson intervals. Multivariable linear regression conducted to see how physical morbidity measures were associated with depressive symptoms. The standardized beta coefficients that are used to express the degree as to which morbidity variables are associated with the PHQ-9 score represent the percent value in which the probability of a yes/no event changes for every one standard deviation change in the PHQ-9 score. All models were adjusted for demographic and socioeconomic characteristics of the respondents, including age (in 5-year groups), sex, place of residence (rural/semi-rural), marital status (not-married/married), education (none/some, but none completed/primary completed or more), ethnicity (Dafin, Mossi, Bwama, other) and a wealth index. We standardized all coefficients so that they represented the standard deviations of change in the PHQ-9 score associated with a one-unit change in morbidity variable. All analyses were conducted using SPSS v25 (IBM, New York, USA)

For peer review only

Ethics

This study received ethics approval from the national health ethics committee in Ouagadougou (#2018-5-053), the CRSN institutional ethics committee in Nouna (#2018-04) and the ethics committee of the medical faculty of the Ruprecht-Karls-Universität Heidelberg (#S-120/2018).

Patients and Public Involvement

Patients and the public were not involved in the design, or conduct, or reporting, or dissemination plans of our research.

RESULTS

Summary statistics for the sample used in the present analysis is presented in Table 1. Of the 3,998 sampled individuals, 3,026 (75.7%) participants were found, consented and completed all questions from the PHQ-9 depression module and were included in the analysis. 1523 (50.3%) of the respondents were women, 1503 men. Women were older on average (mean: 55.5 years, standard deviation (SD): 11.2) than men (mean: 53.1 years, SD: 10.6). They were also more likely to be without formal schooling (90.7%) than men (78.1%), less likely to be married (55.3%, men: 84.7%) and had a higher mean BMI (22.5, SD:5.0; men: 21.8, SD: 3.7). Hypertension, diabetes and hypertriglyceridemia all were equally distributed between both sexes. Elevated blood pressure was highly prevalent in the sample: 27.3% screened positive for grade II hypertension. The prevalence of elevated blood sugar in this population was relatively low (5.0% to 6.8%). 70.8% of the women had an HDL-cholesterol-level below the cut-point to fulfil the criteria for the metabolic syndrome, while only 45.7% of the men had an HDL-cholesterol below the cutoff.

For peer review only

Table 1: Sample characteristics

	**Total** *N* **= 3026**	**Female** *n* **= 1523**	**Male** *n* **= 1503**
**Age group (y)**			
**40-44**	685 (22.6%)	300 (19.7%)	385 (25.6%)
**45-49**	579 (19.1%)	250 (16.4%)	329 (21.9%)
**50-54**	478 (15.8%)	252 (16.5%)	226 (15.0%)
**55-59**	393 (13.0%)	202 (13.3%)	191 (12.7%)
**60-64**	304 (10.0%)	178 (11.7%)	126 (8.4%)
**65-69**	249 (8.2%)	142 (9.3%)	107 (7.1%)
**70-74**	166 (5.5%)	94 (6.2%)	72 (4.8%)
**≥75**	172 (5.7%)	105 (6.9%)	67 (4.5%)
**Place of residence**			
**Nouna**	838 (27.7%)	452 (29.7%)	386 (25.7%)
**Village**	2188 (72.3%)	1071 (70.3%)	1117 (74.3%)
**Ethnicity**			
**Dafin**	1176 (38.9%)	575 (37.8%)	601 (40.0%)
**Bwama**	927 (30.6%)	490 (32.2%)	437 (29.1%)
**Mossi**	403 (13.3%)	199 (13.1%)	204 (13.6%)
**Other**[a]	520 (17.1%)	259 (16.9%)	261 (17.3%)
**Education**			
**No formal schooling**	2555 (84.4%)	1381 (90.7%)	1174 (78.1%)
**Some schooling, but none completed**	253 (8.4%)	79 (5.2%)	174 (11.6%)
**Primary schooling completed & more**	218 (7.2%)	63 (4.1%)	155 (10.3%)
**Currently married**	2115 (69.9%)	842 (55.3%)	1273 (84.7%)
**Body mass index**[b]			
**<18.5 kg/m²**	498 (16.8%)	268 (17.8%)	230 (15.6%)
**18.5-24.9 kg/m²**	1884 (63.4%)	875 (58.3%)	1009 (68.8%)
**25.0-29.9 kg/m²**	441 (14.8%)	249 (16.6%)	192 (13.1%)
**≥30.0 kg/m²**	148 (5.0%)	110 (7.3%)	38 (2.6%)
**Hypertension**[c]			
**Grade I**	1674 (56.3%)	841 (56.0%)	833 (56.7%)
**Grade II**	812 (27.3%)	433 (28.8%)	379 (25.8%)
**Diabetes mellitus**[d]			
**Prediabetes**	974 (45.4%)	530 (46.6%)	444 (44.1%)
**Diabetes**	139 (6.8%)	84 (7.7%)	55 (5.7%)
**Triglycerides (≥150 mg/dl)**[e]	335 (11.6%)	177 (12.0%)	158 (11.2%)
**HDL-cholesterol**[f] **(<40 mg/dl in females, <50 mg/dl in males)**[g]	1366 (58.6%)	849 (70.8%)	517 (45.7%)
**Metabolic Syndrome**[h]	394 (15.6%)	260 (20.4%)	134 (10.7%)
**Self-reported conditions**			
**Elevated blood pressure**	507 (17.1%)	294 (19.8%)	213 (14.4%)
**Elevated blood sugar**	70 (2.3%)	32 (2.1%)	38 (2.6%)
**Heart disease**	166 (5.5%)	116 (7.7%)	50 (3.3%)
**Stroke**	40 (1.3%)	16 (1.1%)	24 (1.6%)
**Self-reported stroke symptoms**			
**Sudden drooping**	58 (1.9%)	33 (2.2%)	25 (1.7%)
**Sudden numbness**	240 (7.9%)	133 (8.7%)	107 (7.1%)
**Sudden slurring**	159 (5.3%)	88 (5.8%)	71 (4.7%)
**Self-reported metabolic syndrome**[i]	688 (23.3%)	396 (26.8%)	292 (19.9%)

[a] Peulh, Samo, Other.
[b] Calculated by weight divided by squared height in meters (kg/m²), 55 missing values due to missing height/weight measurements.

[c] Hypertension grade I: mean systolic blood pressure (BP) ≥ 130 mmHg or mean diastolic BP ≥ 85 mmHg, hypertension grade II: mean systolic BP ≥ 140 mmHg or mean diastolic BP ≥ 90 mmHg. 55 missing values due to missing BP measurements.
[d] Prediabetes: fasting blood glucose (FBG) value between 100 and 126 mg/dl and/or an HbA1c value between 5.7 and 6.5%, 882 missing values due to missing HbA1c measurements and non-fasting results that were excluded. Diabetes: FBG value ≥ 126 mg/dl or an HbA1c value ≥ 6.5 %, 982 missing values due to missing HbA1c measurements and non-fasting results that were excluded.
[e] 135 missing values due to missing Triglyceride measurements.
[f] high-density-lipoprotein-cholesterol
[g] 696 missing values due to missing HDL-cholesterol measurements.
[h] Metabolic syndrome: three of five conditions: (1) body mass index (BMI) ≥ 30 kg/m^2; (2) mean systolic BP ≥ 130 mmHg or mean diastolic BP ≥ 85 mmHg; (3) triglycerides ≥ 150 mg/dl; (4) HDL-cholesterol < 40 mg/dl in men, < 50 mg/dl in women; or (5) FBG ≥ 100 mg/dl. 502 missing values due to missing height, weight, HDL-cholesterol, and Triglyceride measurements as well as non-fasting glucose measurements.
[i] 78 missing values.

Age patterns of depressive symptoms

As Figure 1 shows, the absolute mean values of the z-scored PHQ-9 scores rose continually with age in both men and women. Considering only men, the mean PHQ-9 value for the youngest group of respondents, the 40-44 years old, was -0.41 [95% confidence interval: -0.49 to -0.34]. This value rose constantly up to 0.88 [95% CI: 0.55 to 1.2] within the oldest age group of over 75-years-old. PHQ-9 scores were generally higher among women corresponding to higher levels of depressive symptoms that were also positively correlated with age. The mean value for women in the youngest age group was -0,16 [95% CI: -0.25 to -0.07], rising to 0,80 [95% CI: 0.54 to 1.05] for women in the oldest group.

Age patterns of the metabolic syndrome, diabetes mellitus, and hypertension

Figure 2 shows that the prevalence of the metabolic syndrome increased in a statistically significant way with age for women – it increased from a mean of 12.0% [95% CI: 8.3% to 16.6%) within 40-44-year-olds to 28.2% [95% CI: 18.6% to 39.5%) within the 75+ age group. Surprisingly, the prevalence rate for men declined slightly with age, going from 8.2% [95% CI: 5.4% to 11.8%) to 5.7% [95% CI: 0.0% to 15.7%) – though this difference was not statistically significant. A small increase in the prevalence of prediabetes and diabetes was observed with increasing age, but this difference was not statistically significant. While an increasing prevalence with age could not be shown for diabetes mellitus, we did find a positive association between age

For peer review only

and hypertension. Both men and women showed a rising prevalence of elevated blood pressure with increasing age.

Association of depressive symptoms with the metabolic syndrome, its components, hypertension and diabetes mellitus

The first panel of Table 2 and Figure 3 show the standardized coefficients from simple linear regressions of the z-scored PHQ-9 score on the metabolic syndrome, its components, hypertension and diabetes. In all models, higher age and being female were strongly associated with a higher PHQ-9 score. Having the metabolic syndrome, being positively screened for hypertension, prediabetes or diabetes was not associated with a higher PHQ-9 score. Neither was any of the single components of the metabolic syndrome.

Table 2: Standardized beta coefficients derived from separate multivariable linear regression models investigating the relationship between cardiometabolic and cardiovascular conditions and the z-scored PHQ-9 results.

	Standardized beta coefficient	95% confidence interval	p-value
**Metabolic syndrome**	-0.004	[-0.040, 0.033]	0.847
**Obesity**	0.007	[-0.028, 0.042]	0.680
**Hypertriglyceridemia**	-0.006	[-0.040, 0.029]	0.742
**Reduced HDL-cholesterol**	-0.027	[-0.067, 0.012]	0.177
**Hypertension grade I**	0.028	[-0.007, 0.062]	0.113
**FPG > 100**	-0.022	[-0.061, 0.019]	0.298
**Hypertension grade II**	0.014	[-0.020, 0.048]	0.427
**Prediabetes**	0.005	[-0.034, 0.043]	0.819
**Diabetes**	-0.001	[-0.040, 0.039]	0.972
**Self-reported conditions**			
**Hypertension**	0.032	[-0.003, 0.066]	0.071
**Diabetes**	0.004	[-0.029, 0.038]	0.809
**Heart disease**	0.081	[0.047, 0.114]	<0.001
**Stroke**	0.038	[0.005, 0.071]	0.024
**Self-reported stroke symptoms**			
**Sudden drooping**	0.090	[0.057, 0.123]	<0.001
**Sudden numbness**	0.136	[0.102, 0.168]	<0.001
**Sudden slurring**	0.144	[0.110, 0.176]	<0.001
**Self-reported metabolic syndrome**	0.070	[0.035, 0.104]	<0.001

Each standardized coefficient arises from a separate model controlling for age, sex, gender, place of residence, education, ethnicity and wealth quintile. The separate models can be found in Supplementary Materials (Supplementary Tables 1-17).

For peer review only

Association of depressive symptoms with self-reported conditions and stroke symptoms

Figure 3 shows that having previously been diagnosed by a health care worker with elevated blood pressure or elevated blood sugar was not significantly associated with the PHQ-9 score, whereas the models did show a positive association for ever having been diagnosed with a stroke (standardized beta coefficient (hereafter ‘beta’) = 0.04 [95% confidence interval: 0.01 to 0.07]) or heart disease (beta = 0.08 [95% CI: 0.05 to 0.11]). An even stronger association could be shown for reporting to ever having had a sudden drooping of one side of the face (beta = 0.09 [95% CI: 0.06 to 0.12]), a sudden numbness weakness or dead feeling on one half of the body (beta = 0.14 [95% CI: 0.10 to 0.17]) and a sudden slurring of speech (beta = 0.14 [95% CI: 0.11 to 0.18]).

The ‘self-reported metabolic syndrome’ with one or more out of four self-reported health items present was associated positively with the PHQ-9 score (beta = 0.07 [95% CI: 0.04 to 0.10]). The results did not change substantially when running the regression models for the self-reported health items within the parts of the population who were screened positively for the respective condition, e.g. the model with self-reported elevated blood pressure within the population who were measured as hypertensive (Supplementary Tables 18-20).

DISCUSSION

Depressive symptoms were not associated with the metabolic syndrome, hypertension, diabetes mellitus and past diagnosis of elevated blood pressure or blood sugar. Prior stroke diagnosis or heart disease were positively associated with the standardized PHQ-9 score, as were self-reported stroke symptoms. Additionally, the prevalence of depressive symptoms rose substantially with increasing age for both men and women, confirming similar findings from recent studies investigating depression in SSA and standing in contrast to findings from high income countries. The findings indicate that in a setting where cardiovascular conditions are not

For peer review only

the dominant health issue, non-symptomatic conditions like low-grade hypertension do not have detrimental effects on people's mental health.

Chronic conditions and depressive symptoms

The findings suggest that the metabolic syndrome, hypertension and diabetes have no significant association with depressive symptoms in our sample. This situation is congruent with recent studies from SSA investigating the relationship between CVD and depression. Gelaye et al. [42] did not find an association between depression and the metabolic syndrome or diabetes in a sample of Ethiopian adults, Geldsetzer et al. [22] showed that there was no association between depression and hypertension, diabetes and obesity in a South African sample. In contrast to these results from SSA, relationships between CVD and depression have been widely reported in HIC [3-6,43]. In SSA, NCD including CVD are only recently emerging as a public health concern, while infectious diseases are still the most important cause of death [44]. People and health systems are more attuned to seeing and treating acute infections and maternal health problems than CVD, which might lead to a lower degree of understanding of what it means to have CVD – hence protecting the mental health of people in SSA. This hypothesis will need to be tested in future studies. Individuals with a higher socioeconomic status (SES) can afford higher health care costs than those with a lower SES, for whom it is a greater burden to pay for their health. Fiske et al. [17] found that unaffordable health care costs for individuals affected by CVD have a negative effect on their mental health.

CVD risk factors are asymptomatic, especially in their early stages, which might reduce the negative effects of CVD on mental health. Research in HIC has shown that there are biological factors like chronic inflammation, vascular endothelial dysfunction, neuroendocrine disequilibrium (particularly of the pituitary-adrenal axis) and increased platelet activation that can cause depression in people with CVD and viceversa [45,46]. It remains unclear in the sub-Saharan African context if these biomedical processes are similarly important in driving an association between depressive symptoms and CVD; further research is needed to investigate this question.

For peer review only

Multimorbidity might be another factor linking CVD and depression in HIC [47]. According to a recent study conducted in the same HDSS as this study, multimorbidity is highly prevalent in this sample [48]. Whether experiencing other NCDs in addition to CVD – for instance cancer, COPD, musculoskeletal disorders and other mental illnesses – has an effect on the relationship between depressive symptoms and CVD has not been examined and could be subject to further investigations. Use of antidepressants is linked to a higher risk of obesity, diabetes, and CVD [49] and could thus act as a confounding factor in studies in HIC investigating the association between depressive symptoms and cardiometabolic risk. In Burkina Faso the availability of specialized care is poor [50] and so is the availability of antidepressants, which makes it less likely for Burkinabé to experience the adverse outcomes of antidepressants.

Consequences of CVD

Interestingly, our findings suggest that having experienced the consequences of a stroke as well as having ever been diagnosed with a stroke or heart disease is associated with higher PHQ-9 results. These associations are in congruence with the situation in high-income countries, where especially ischemic events like heart attacks and strokes due to CVD are associated with depression [45]. Ischemic events may lead to physical dysfunction, impairing the ability to work in physical jobs that are important for the social and economic livelihood in a rural SSA environment. Social insecurity and disintegration resulting from these limitations are substantial risk factors to develop depressive symptoms. According to Schatz and Seeley [51], older adults in SSA strongly link their physical functionality to the concept of seeing themselves as independent individuals. With declines in physical functioning, their perceived independence deteriorates, leaving individuals as increasingly dependent and at risk of depression. Limited economic resources hinder the ability of older adults in rural areas to cope with stressors like disability after stroke, and also act to limit access to an already weak health system. This paucity of diagnosis and treatment options also acts as part of a vicious cycle, as it may lead to substantial delays in accessing the health care system [52]. These barriers to mental health care lead to a lack of knowledge and awareness of an individual’s own physical and mental health,

For peer review only

making it much less likely to engage in protective behaviors such as regular physical activity, avoiding stimulants and drugs, engaging in stabile relationships or adjusting sleep rhythms and diet[53].

Sociodemographics

Women had a higher prevalence of depressive symptoms than men in our sample, showing that it will be especially important to target women in future interventions. With increasing age, the prevalence of depressive symptoms rose substantially for both men and women, confirming similar findings from recent studies investigating depression in SSA [22,23]. Mechanisms that protect older populations in HIC include an increasing and positive influence of religiosity, gathered wisdom and life experience, higher economic resources, intensified social engagement and socio-emotional selectivity [17,18]. Examining whether these resilience mechanisms apply to the SSA context should be a focus for future research.

Implications

The findings suggest that improving care for measurable CVD conditions – metabolic syndrome, hypertension and diabetes – may have additional benefits for improving depressive symptoms. Even though they might not be directly associated with depressive symptoms, these conditions increase the likelihood of thromboembolic events which are associated with worse mental health. When depression is detected in its early stages, it might also be possible to prevent a part of CVD burden by engaging in preventive diagnostics and treatment in a targeted part of the population. While it is highly prevalent and likely an important correlate and basis for other chronic conditions and care success, depression has been woefully neglected in the global health response. As diagnostic and therapeutic options exist, this gap should be addressed by policymakers as soon as possible.

For peer review only

Strengths and limitations

Our study had several strengths. It was large and population-based with a randomly selected sample, in contrast to the selective nature of almost all prior studies about depression in Burkina Faso, with the notable exception of Ouédraogo et al. [29]. The use of both questionnaire and biomarker testing allowed objective measurement of chronic conditions, using internationally standardized values allowing comparison to studies elsewhere. Furthermore, the PHQ-9 instrument is locally applicable, being widely used in population-based studies and primary care settings in SSA [23,54-56]. Nevertheless, its translation into the local language, particularly the collaborative manner in which it was done, makes it vulnerable to differences in expression during interviews. While we have adjusted for differential understanding by standardizing scores at the interviewer level, cultural differences in expressing psychological states like sadness may still be important. The cross-sectional design of the questionnaire and the one-time sample do not make it possible to draw causal interpretations from the findings or to confirm diagnoses of hypertension, diabetes and depression. Finally, our sample of adults aged 40 and above is not directly comparable to the typically older cut-offs for aging studies; however given the life expectancy of Burkinabé people (60.8 years [57]) we believe that the respondents represent old individuals within this setting. Clearly our findings only apply to over-40s, and associations may differ in younger individuals.

Our analyses do not account for the potential that some individuals in our sample may be on medication to control a cardiovascular condition. Respondents who self-reported having hypertension, elevated blood sugar, hypercholesterolemia, or heart disease were asked a single combined question on whether they had received any treatment in the last two weeks. However, the definition of "treatment" was not specified any further, and therefore may represent treatments as varied as medication, physical exercise, dietary change, or traditional healing. Self-reported rates of treatment were generally low, with only 11% of those with hypertension and 33% of those with self-reported hypertension reported having received treatment in the last two weeks. The numbers for elevated blood sugar were even lower: 8.6% of those with diabetes and

For peer review only

22.9% of those with self-reported elevated blood sugar said they had received treatment in the last two weeks. Our data showing low treatment rates are consistent with studies in SSA that document similar patterns for hypertension [58-61] and diabetes [62]. The non-specific nature of the available treatment variable means that any analyses using this variable would be nearly impossible to meaningfully interpret, and we therefore chose to omit self-reported treatment from our analyses.

Conclusion

In conclusion, we find that having the metabolic syndrome, hypertension or diabetes was not associated with the results of the PHQ-9 score among a group of older adults in a rural, sub-Saharan African setting. Interestingly, an association could be found between depressive symptoms and having experienced stroke symptoms as well as self-reported diagnose of stroke and heart disease. This might be due to the influence of disability after thromboembolic events on the psychological well-being of African adults seen in prior research. It may also be seen as a development foreshadowing an association of CVD and depression in this population, which cannot be seen yet because of lacking awareness, diagnosis and treatment options. Age was strongly associated with depressive symptoms, confirming the age patterns found in previous studies in SSA.

Acknowledgements

Funding for the CRSN Heidelberg Aging Study (CHAS) was provided by the Alexander von Humboldt Foundation through an Alexander von Humboldt Professor award to TB, which is funded by the German Federal Ministry of Education and Research. MDW acknowledges support from NIHR Newcastle Biomedical Research Centre. GH is supported by a fellowship from the Wellcome Trust and Royal Society (210479/Z/18/Z). The funders had no role in study design, data collection and analysis, decision to publish, or preparation of the manuscript.

For peer review only

Conflict of Interest

None reported.

Data availability

Data are not publicly available as consent was not given by participants for data to be shared openly. This is in part because entire age cohorts of some villages are included in the dataset, potentially allowing for deductive disclosure with sufficient local information. For this reason, anonymized data is available from CHAS study data controllers only following signature of a data use agreement restricting onward transmission. Anyone wishing to replicate the analyses presented, or conduct further collaborative analyses using CHAS (which are welcomed and considered based on a letter of intent), should contact Dr Guy Harling (g.harling@ucl.ac.uk) in the first instance.

Contributors

TB and GH conceived and designed the overall CRSN CHAS study. CP, IK, JD, MW and MS contributed to the design of the CRSN CHAS household survey. BB, GH, AS, MB, LO and BC coordinated baseline data collection and preparation. BB conducted the analysis, and wrote, and revised the manuscript. CP and IK supervised the analysis, write up, and development of the manuscript. All authors substantively reviewed manuscripts, inputted into revisions, and approved the final manuscript.

For peer review only

REFERENCES

1 WHO. Depression and Other Common Mental Disorders: Global Health Estimates. 2017.

2 James SL, Abate D, Abate KH, Abay SM, Abbafati C, Abbasi N, et al. Global, regional, and national incidence, prevalence, and years lived with disability for 354 diseases and injuries for 195 countries and territories, 1990–2017: a systematic analysis for the Global Burden of Disease Study 2017. The Lancet. 2018;392:1789-858.

3 Repousi N, Masana MF, Sanchez-Niubo A, Haro JM, Tyrovolas S. Depression and metabolic syndrome in the older population: A review of evidence. J Affect Disord. 2018;237:56-64.

4 Ghanei Gheshlagh R, Parizad N, Sayehmiri K. The Relationship Between Depression and Metabolic Syndrome: Systematic Review and Meta-Analysis Study. Iran Red Crescent Med J. 2016;18:e26523-e.

5 Marazziti D, Rutigliano G, Baroni S, Landi P, Dell'Osso L. Metabolic syndrome and major depression. CNS Spectrums. 2014;19:293-304.

6 Vaccarino V, McClure C, Johnson BD, Sheps DS, Bittner V, Rutledge T, et al. Depression, the metabolic syndrome and cardiovascular risk. Psychosom Med. 2008;70:40-8.

7 Stein DJ, Benjet C, Gureje O, Lund C, Scott KM, Poznyak V, et al. Integrating mental health with other non-communicable diseases. BMJ. 2019;364:l295.

8 Bao AM, Meynen G, Swaab DF. The stress system in depression and neurodegeneration: Focus on the human hypothalamus. Brain Research Reviews. 2008;57:531-53.

9 Björntorp P. Do stress reactions cause abdominal obesity and comorbidities? Obesity Reviews. 2001;2:73-86.

10 Carney RM, Freedland KE, Veith RC. Depression, the Autonomic Nervous System, and Coronary Heart Disease. Psychosomatic Medicine. 2005;67:S29-S33.

11 Golden SH. A review of the evidence for a neuroendocrine link between stress, depression and diabetes mellitus. Curr Diabetes Rev. 2007;3:252-9.

12 Carnethon MR, Biggs ML, Barzilay JI, Smith NL, Vaccarino V, Bertoni AG, et al. Longitudinal Association Between Depressive Symptoms and Incident Type 2 Diabetes Mellitus in Older Adults: The Cardiovascular Health Study. Archives of Internal Medicine. 2007;167:802-7.

13 Ford DE, Erlinger TP. Depression and C-Reactive Protein in US Adults: Data From the Third National Health and Nutrition Examination Survey. Archives of Internal Medicine. 2004;164:1010-4.

For peer review only

14 Pan A, Sun Q, Okereke OI, Rexrode KM, Hu FB. Depression and Risk of Stroke Morbidity and Mortality: A Meta-analysis and Systematic Review. JAMA. 2011;306:1241-9.

15 Skala JA, Freedland KE, Carney RM. Coronary Heart Disease and Depression: A Review of Recent Mechanistic Research. The Canadian Journal of Psychiatry. 2006;51:738-45.

16 Huang C-Q, Dong B-R, Lu Z-C, Yue J-R, Liu Q-X. Chronic diseases and risk for depression in old age: A meta-analysis of published literature. Ageing Research Reviews. 2010;9:131-41.

17 Fiske A, Wetherell JL, Gatz M. Depression in older adults. Annu Rev Clin Psychol. 2009;5:363-89.

18 Blazer DG, Hybels CF. Origins of depression in later life. Psychol Med. 2005;35:1241-52.

19 Robinson RG, Jorge RE. Post-Stroke Depression: A Review. American Journal of Psychiatry. 2015;173:221-31.

20 Ayerbe L, Ayis S, Wolfe CDA, Rudd AG. Natural history, predictors and outcomes of depression after stroke: systematic review and meta-analysis. British Journal of Psychiatry. 2013;202:14-21.

21 Lichtman Judith H, Froelicher Erika S, Blumenthal James A, Carney Robert M, Doering Lynn V, Frasure-Smith N, et al. Depression as a Risk Factor for Poor Prognosis Among Patients With Acute Coronary Syndrome: Systematic Review and Recommendations. Circulation. 2014;129:1350-69.

22 Geldsetzer P, Vaikath M, Wagner R, Rohr JK, Montana L, Gomez-Olive FX, et al. Depressive Symptoms and Their Relation to Age and Chronic Diseases Among Middle-Aged and Older Adults in Rural South Africa. J Gerontol A Biol Sci Med Sci. 2019;74:957-63.

23 Kohler IV, Payne CF, Bandawe C, Kohler HP. The Demography of Mental Health Among Mature Adults in a Low-Income, High-HIV-Prevalence Context. Demography. 2017;54:1529-58.

24 Blazer DG. Depression in late life: review and commentary. J Gerontol A Biol Sci Med Sci. 2003;58:249-65.

25 Thapa SB, Martinez P, Clausen T. Depression and its correlates in South Africa and Ghana among people aged 50 and above: Findings from the WHO Study on global AGEing and adult health. Journal of psychiatry. 2014;17:1000167.

26 Yameogo NV, Samadoulougou A, Kagambega LJ, Yameogo AA, Ilboudo E, Millogo G, et al. [Sleep disorders, anxiety and depressive symptoms and cardiovascular risk among black African hypertensive: cross-sectional study of 414 hypertensive followed as outpatients at the University Hospital of Ouagadougou (Burkina Faso)]. Pan Afr Med J. 2015;21:115.

For peer review only

27 Napon C, Kaboré A, Kaboré J. Post stroke depression in Burkina Faso. The Pan African medical journal. 2012;13:3-.

28 Duthe G, Rossier C, Bonnet D, Soura AB, Corker J. Mental health and urban living in sub-Saharan Africa: major depressive episodes among the urban poor in Ouagadougou, Burkina Faso. Popul Health Metr. 2016;14:18.

29 Ouédraogo A, Ouango JG, Karfo K, Goumbri P, Nanéma D, Sawadogo B. Prévalence des troubles mentaux en population générale au Burkina Faso. L'Encéphale. 2019;45:367-70.

30 Sie A, Louis VR, Gbangou A, Muller O, Niamba L, Stieglbauer G, et al. The Health and Demographic Surveillance System (HDSS) in Nouna, Burkina Faso, 1993-2007. Glob Health Action. 2010;3.

31 Kroenke K, Spitzer RL, Williams JB, Lowe B. The Patient Health Questionnaire Somatic, Anxiety, and Depressive Symptom Scales: a systematic review. Gen Hosp Psychiatry. 2010;32:345-59.

32 Sweetland AC, Belkin GS, Verdeli H. Measuring depression and anxiety in sub-saharan Africa. Depression and anxiety. 2014;31:223-32.

33 Cuijpers P, Vogelzangs N, Twisk J, Kleiboer A, Li J, Penninx BW. Differential mortality rates in major and subthreshold depression: meta-analysis of studies that measured both. British Journal of Psychiatry. 2013;202:22-7.

34 Johnson J, Weissman MM, Klerman GL. Service Utilization and Social Morbidity Associated With Depressive Symptoms in the Community. JAMA. 1992;267:1478-83.

35 Alberti KG, Eckel RH, Grundy SM, Zimmet PZ, Cleeman JI, Donato KA, et al. Harmonizing the metabolic syndrome: a joint interim statement of the International Diabetes Federation Task Force on Epidemiology and Prevention; National Heart, Lung, and Blood Institute; American Heart Association; World Heart Federation; International Atherosclerosis Society; and International Association for the Study of Obesity. Circulation. 2009;120:1640-5.

36 Whelton PK, Carey RM. The 2017 American College of Cardiology/American Heart Association Clinical Practice Guideline for High Blood Pressure in Adults. JAMA Cardiol. 2018;3:352-3.

37 American Diabetes A. 2. Classification and Diagnosis of Diabetes: Standards of Medical Care in Diabetes-2018. Diabetes Care. 2018;41:S13-S27.

38 Kothari RU, Pancioli A, Liu T, Brott T, Broderick J. Cincinnati Prehospital Stroke Scale: Reproducibility and Validity. Annals of Emergency Medicine. 1999;33:373-8.

For peer review only

39 Kolenikov S, Angeles G. Socioeconomic Status Measurement With Discrete Proxy Variables: Is Principal Component Analysis A Reliable Answer? Review of Income and Wealth. 2009;55:128-65.

40 Filmer D, Pritchett LH. Estimating Wealth Effects Without Expenditure Data—Or Tears: An Application To Educational Enrollments In States Of India*. Demography. 2001;38:115-32.

41 Rutstein SO, Johnson K. The DHS wealth index. Calverton, Maryland, USA: ORC Macro, 2004.

42 Gelaye B, Williams MA, Lemma S, Berhane Y, Fann JR, Vander Stoep A, et al. Major depressive disorder and cardiometabolic disease risk among sub-Saharan African adults. Diabetes & metabolic syndrome. 2015;9:183-91.

43 Pan A, Keum N, Okereke OI, Sun Q, Kivimaki M, Rubin RR, et al. Bidirectional association between depression and metabolic syndrome: a systematic review and meta-analysis of epidemiological studies. Diabetes care. 2012;35:1171-80.

44 Institute for Health Metrics and Evaluation (IHME). GBD Compare Data Visualization. Seattle, WA: IHME, University of Washington. Available from http://vizhub.healthdata.org/gbd-compare. (Accessed 9th of November 2019). 2018.

45 Goldston K, Baillie AJ. Depression and coronary heart disease: A review of the epidemiological evidence, explanatory mechanisms and management approaches. Clinical Psychology Review. 2008;28:288-306.

46 Krishnan KR, Delong M, Kraemer H, Carney R, Spiegel D, Gordon C, et al. Comorbidity of depression with other medical diseases in the elderly. Biol Psychiatry. 2002;52:559-88.

47 Søgaard M, Skjøth F, Kjældgaard JN, Larsen TB, Hjortshøj SP, Riahi S. Atrial fibrillation in patients with severe mental disorders and the risk of stroke, fatal thromboembolic events and bleeding: a nationwide cohort study. BMJ open. 2017;7:e018209-e.

48 Odland ML, Payne C, Witham M, Siedner M, Bärnighausen T, Bountogo M, et al. The epidemiology of multimorbidity in conditions of extreme poverty: a population-based study of older adults in rural Burkina Faso. Manuscript submitted for publication. 2020.

49 Correll CU, Detraux J, De Lepeleire J, De Hert M. Effects of antipsychotics, antidepressants and mood stabilizers on risk for physical diseases in people with schizophrenia, depression and bipolar disorder. World Psychiatry. 2015;14:119-36.

50 Maiga DD, Eaton J. A survey of the mental healthcare systems in five Francophone countries in West Africa: Bénin, Burkina Faso, Côte d'Ivoire, Niger and Togo. International Psychiatry. 2014;11:69-72.

For peer review only

51 Schatz E, Seeley J. Gender, ageing and carework in East and Southern Africa: A review. Glob Public Health. 2015;10:1185-200.

52 Collins KA, Westra HA, Dozois DJ, Burns DD. Gaps in accessing treatment for anxiety and depression: challenges for the delivery of care. Clin Psychol Rev. 2004;24:583-616.

53 Sarris J, O'Neil A, Coulson CE, Schweitzer I, Berk M. Lifestyle medicine for depression. BMC psychiatry. 2014;14:107-.

54 Motlhatlhedi K, Setlhare V, Ganiyu AB, Firth JA. Association between depression in carers and malnutrition in children aged 6 months to 5 years. African Journal of Primary Health Care & Family Medicine. 2017;9:1-6.

55 L'Akoa R M, Noubiap JJ, Fang Y, Ntone FE, Kuaban C. Prevalence and correlates of depressive symptoms in HIV-positive patients: a cross-sectional study among newly diagnosed patients in Yaounde, Cameroon. BMC Psychiatry. 2013;13:228.

56 Bindt C, Appiah-Poku J, Te Bonle M, Schoppen S, Feldt T, Barkmann C, et al. Antepartum depression and anxiety associated with disability in African women: cross-sectional results from the CDS study in Ghana and Cote d'Ivoire. PLoS One. 2012;7:e48396.

57 United Nations, Department of Economic and Social Affairs, Population Division. World Population Prospects 2019, custom data acquired via website. Accessed 23rd March 2020. 2019.

58 Cappuccio Francesco P, Micah Frank B, Emmett L, Kerry Sally M, Antwi S, Martin-Peprah R, et al. Prevalence, Detection, Management, and Control of Hypertension in Ashanti, West Africa. Hypertension. 2004;43:1017-22.

59 Ataklte F, Erqou S, Kaptoge S, Taye B, Echouffo-Tcheugui Justin B, Kengne Andre P. Burden of Undiagnosed Hypertension in Sub-Saharan Africa. Hypertension. 2015;65:291-8.

60 Hendriks ME, Wit FWNM, Roos MTL, Brewster LM, Akande TM, de Beer IH, et al. Hypertension in Sub-Saharan Africa: Cross-Sectional Surveys in Four Rural and Urban Communities. PLOS ONE. 2012;7:e32638.

61 Musinguzi G, Nuwaha F. Prevalence, Awareness and Control of Hypertension in Uganda. PLOS ONE. 2013;8:e62236.

62 Nuche-Berenguer B, Kupfer LE. Readiness of Sub-Saharan Africa Healthcare Systems for the New Pandemic, Diabetes: A Systematic Review. Journal of Diabetes Research. 2018;2018:9262395.

For peer review only

1 **FIGURES**

2 **Figure 1:** Mean PHQ-9 scores (z-scored within interviewer) and 95% confidence intervals.
3 Underlying data can be found in Supplementary Materials (Supplementary Table 21)

4 **Figure 2:** Prevalence of the metabolic syndrome, pre-diabetes, diabetes and hypertension grade
5 II; 95% confidence intervals. Underlying data can be found in Supplementary Materials
6 (Supplementary Tables 22 – 25)

7 **Figure 3:** Forest plot of associations between non-communicable conditions and PHQ-9 z-
8 scores.

For peer review only

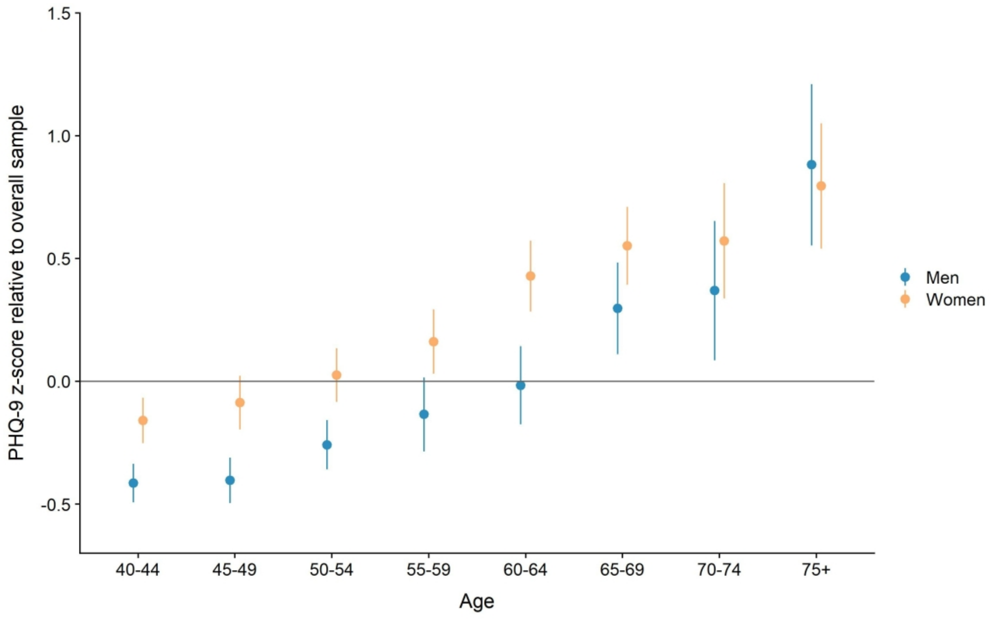

Figure 1: Mean PHQ-9 scores (z-scored within interviewer) and 95% confidence intervals. Underlying data can be found in Supplementary Materials (Supplementary Table 21).

246x152mm (300 x 300 DPI)

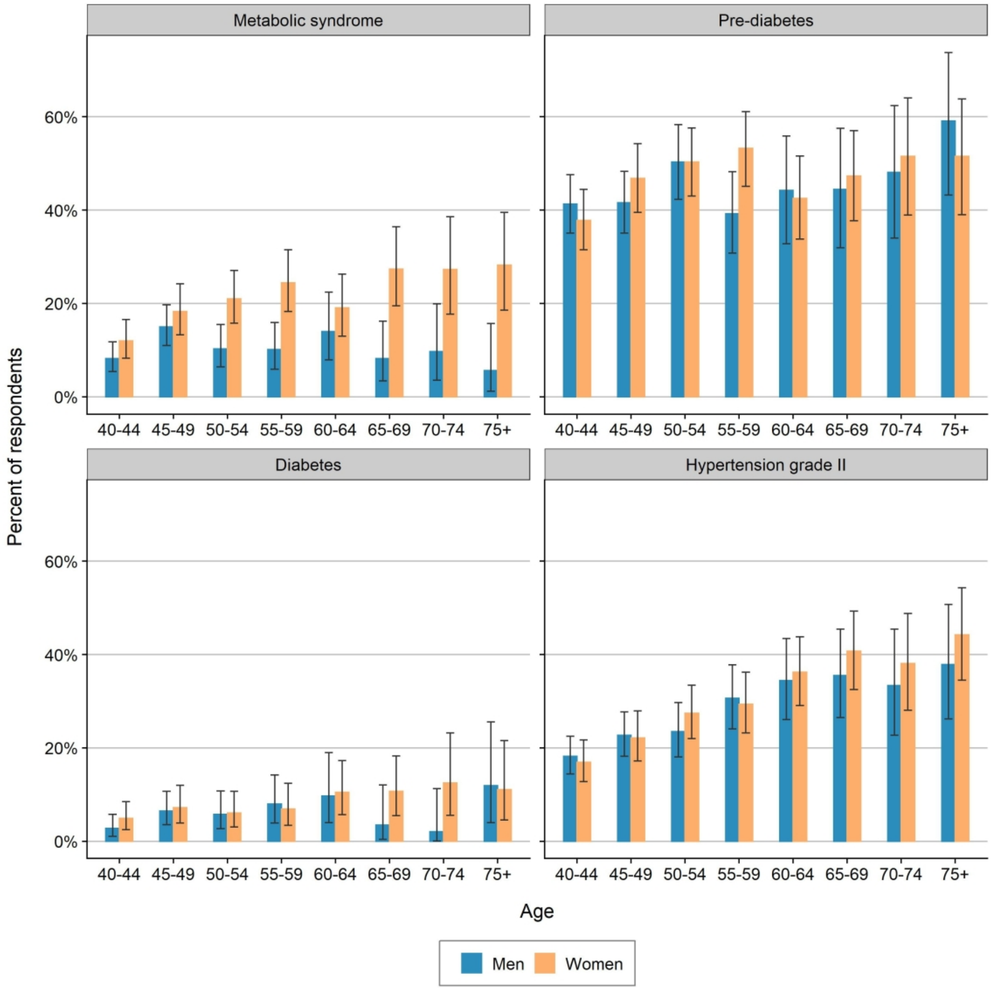

Figure 2: Prevalence of the metabolic syndrome, pre-diabetes, diabetes and hypertension grade II; 95% confidence intervals. Underlying data can be found in Supplementary Materials (Supplementary Tables 22 – 25).

254x254mm (300 x 300 DPI)

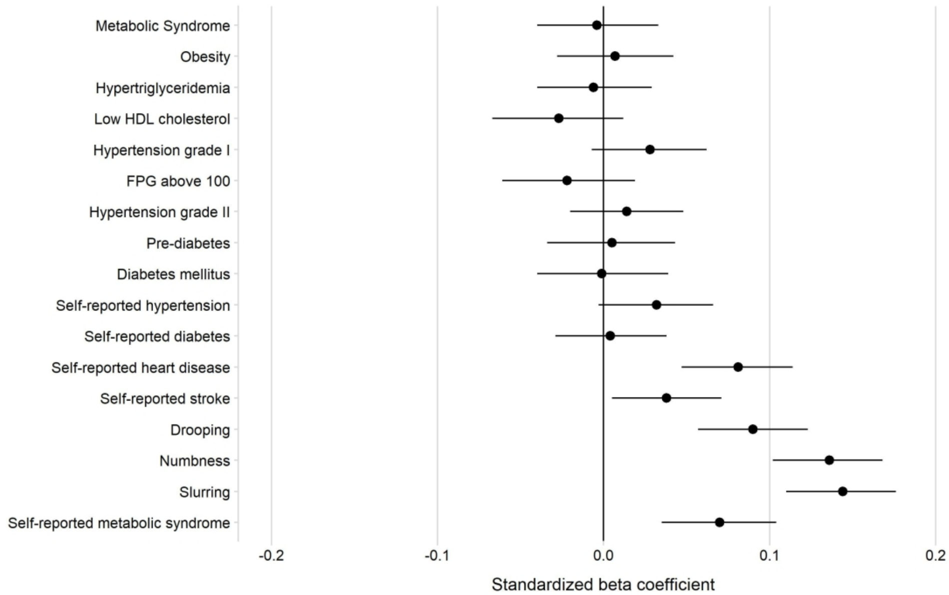

Figure 3: Forest plot of associations between non-communicable conditions and PHQ-9 z-scores.

246x152mm (300 x 300 DPI)

SUPPLEMENTARY MATERIAL

Supplementary Figure 1: This figure shows the distribution of the mean raw PHQ-9 values by single interviewers. The differences between interviewers' PHQ-9 values were interpreted as an interviewer effect during analyses. We decided to standardize the results on the interviewer level and add them up afterwards.

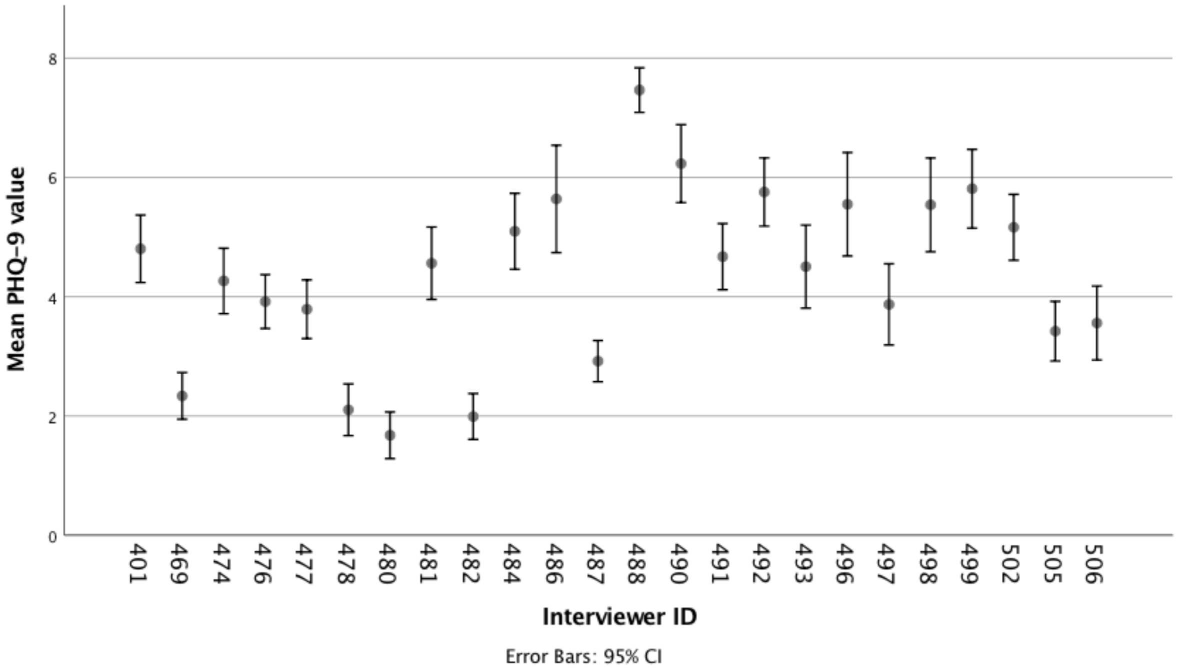

Supplementary Figure 2: The figure shows the standardized PHQ-9 values of one of the interviewers (401) and the normal distribution curve. We z-scored the PHQ-9 results for this interviewer with the standardization function in SPSS. Each value was calculated by dividing the difference between the value and the mean by the standard deviation. This was done for all interviewers separately. Afterwards, all values were included in the outcome variable of the z-scored PHQ-9 score.

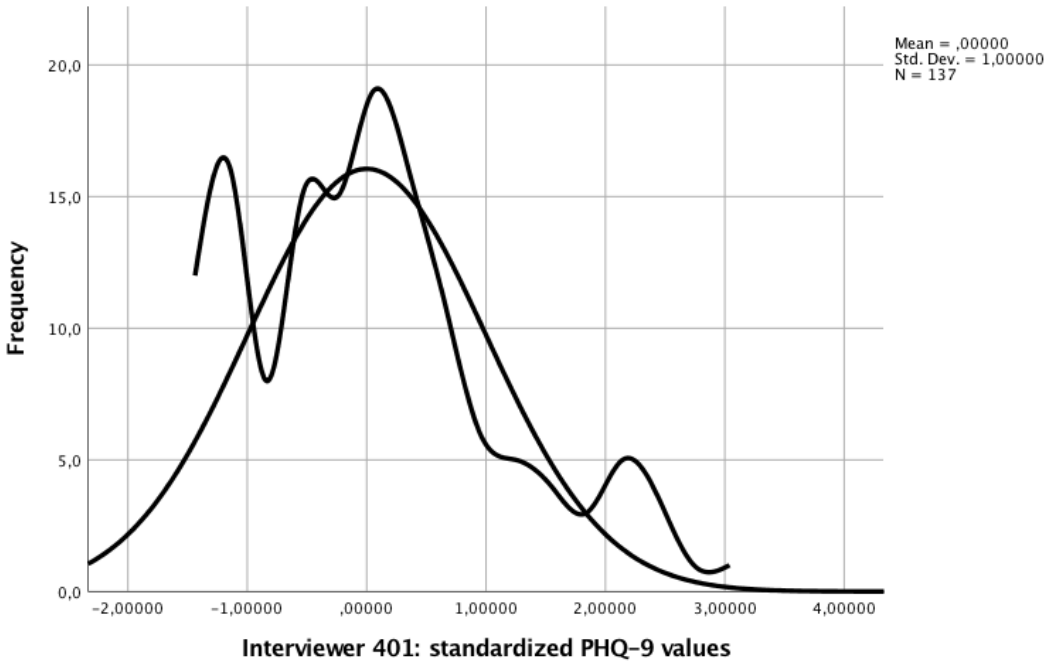

Supplementary Table 1: Linear regression model 1 (Metabolic syndrome)

Dependent variable: Z-scored PHQ-9

		Model 1 (n=2524)			
		beta	CI low	CI high	p
Age	40-44	-	-	-	-
	45-49	.007	-.037	.050	.773
	50-54	.058	.014	.098	.010
	55-59	.098	.053	.136	<.001
	60-64	.144	.101	.184	<.001
	65-69	.182	.139	.221	<.001
	70-74	.147	.104	.183	<.001
	75+	.251	.215	.298	<.001
Sex (ref: male)		.115	.074	.151	<.001
Living in the countryside		-.052	-.088	-.011	.013
Currently married		-.020	-.065	.022	.340
Education	None				
	Some	.018	-.020	.054	.360
	Primary & more	-.049	-.088	-.009	.016
Ethnicity	Dafin				
	Bwama	.017	-.022	.053	.430
	Mossi	.007	-.028	.040	.734
	Other	.022	-.024	.082	.280
Wealth quintile	1				
	2	-.030	-.074	.016	.208
	3	-.051	-.096	-.003	.038
	4	-.058	-.103	-.009	.019
	5	-.083	-.135	-.033	.001
**Metabolic syndrome**		-.004	-.040	.033	.847

For peer review only

Supplementary Table 2: Linear regression model 2
Abdominal Obesity

Dependent variable: Z-scored PHQ-9

		Model 2 (n=2970)			
		beta	CI low	CI high	p
Age	40-44	-	-	-	-
	45-49	.017	-.024	.058	.406
	50-54	.059	.019	.099	.004
	55-59	.101	.062	.140	<.001
	60-64	.143	.104	.181	<.001
	65-69	.189	.150	.226	<.001
	70-74	.164	.126	.200	<.001
	75+	.239	.200	.275	<.001
Sex (ref: male)		.118	.081	.153	<.001
Living in the countryside		-.047	-.078	-.008	.016
Currently married		-.028	-.072	.010	.143
Education	None				
	Some	.019	-.016	.053	.288
	Primary & more	-.033	-.071	.003	.072
Ethnicity	Dafin				
	Bwama	.021	-.017	.055	.302
	Mossi	-.001	-.033	.031	.944
	Other	.026	-.015	.080	.180
Wealth quintile	1				
	2	-.020	-.062	.023	.372
	3	-.056	-.100	-.012	.013
	4	-.066	-.110	-.021	.004
	5	-.087	-.135	-.040	<.001
**Abdominal Obesity**		.007	-.028	.042	.680

For peer review only

Supplementary Table 3: Linear regression model 3 (Hypertriglyceridemia)

Dependent variable: Z-scored PHQ-9

		Model 3 (n=2890)			
		beta	CI low	CI high	p
Age	40-44	-	-	-	-
	45-49	.013	-.028	.055	.531
	50-54	.064	.022	.104	.002
	55-59	.098	.057	.137	<.001
	60-64	.144	.103	.182	<.001
	65-69	.186	.144	.221	<.001
	70-74	.159	.120	.194	<.001
	75+	.243	.203	.280	<.001
Sex (ref: male)		.115	.078	.150	<.001
Living in the countryside		-.052	-.083	-.012	.009
Currently married		-.029	-.073	.010	.141
Education	None				
	Some	.017	-.018	.052	.330
	Primary & more	-.041	-.079	-.004	.029
Ethnicity	Dafin				
	Bwama	.015	-.023	.050	.456
	Mossi	.001	-.031	.034	.938
	Other	.025	-.017	.080	.200
Wealth quintile	1				
	2	-.024	-.067	.020	.285
	3	-.054	-.099	-.010	.017
	4	-.069	-.113	-.023	.003
	5	-.083	-.132	-.036	.001
**Hypertriglyceridemia**		-.006	-.040	.029	.742

For peer review only

Supplementary Table 4: Linear regression model 4
(Reduced HDL-cholesterol)

Dependent variable: Z-scored PHQ-9

		Model 4 (n=2330)			
		beta	CI low	CI high	p
Age	40-44	-	-	-	-
	45-49	.019	-.028	.065	.425
	50-54	.061	.015	.106	.009
	55-59	.110	.063	.151	<.001
	60-64	.154	.107	.193	<.001
	65-69	.185	.141	.227	<.001
	70-74	.170	.124	.206	<.001
	75+	.234	.196	.283	<.001
Sex (ref: male)		.134	.091	.174	<.001
Living in the countryside		-.028	-.078	.016	.193
Currently married		-.031	-.079	.013	.158
Education	None				
	Some	.004	-.034	.042	.843
	Primary & more	-.035	-.084	.006	.092
Ethnicity	Dafin				
	Bwama	.007	-.032	.045	.745
	Mossi	-.016	-.051	.022	.437
	Other	.010	-.048	.080	.620
Wealth quintile	1				
	2	-.035	-.080	.012	.147
	3	-.059	-.106	-.010	.017
	4	-.075	-.121	-.025	.003
	5	-.072	-.133	-.026	.004
**Reduced HDL-cholesterol**		-.027	-.067	.012	.177

For peer review only

Supplementary Table 5: Linear regression model 5 (Hypertension grade I)

Dependent variable: Z-scored PHQ-9

		Model 5 (n=2970)			
		beta	CI low	CI high	p
Age	40-44	-	-	-	-
	45-49	.016	-.025	.057	.441
	50-54	.057	.017	.097	.006
	55-59	.098	.058	.137	<.001
	60-64	.139	.099	.177	<.001
	65-69	.185	.145	.222	<.001
	70-74	.161	.123	.197	<.001
	75+	.235	.196	.271	<.001
Sex (ref: male)		.119	.082	.154	<.001
Living in the countryside		-.046	-.077	-.007	.020
Currently married		-.028	-.072	.010	.141
Education	None				
	Some	.020	-.015	.054	.257
	Primary & more	-.034	-.071	.002	.067
Ethnicity	Dafin				
	Bwama	.020	-.018	.054	.326
	Mossi	.000	-.032	.032	.982
	Other	.027	-.014	.081	.164
Wealth quintile	1				
	2	-.019	-.062	.024	.379
	3	-.055	-.099	-.011	.014
	4	-.066	-.110	-.022	.004
	5	-.087	-.134	-.040	<.001
**Hypertension grade I**		.028	-.007	.062	.113

For peer review only

Supplementary Table 6: Linear regression model 6
(Fasting Plasma Glucose > 100 mg/dl)

Dependent variable: Z-scored PHQ-9

		Model 6 (n=2038)			
		beta	CI low	CI high	p
Age	40-44	-	-	-	-
	45-49	.002	-.046	.049	.939
	50-54	.049	.000	.093	.048
	55-59	.102	.053	.144	<.001
	60-64	.143	.096	.188	<.001
	65-69	.192	.144	.236	<.001
	70-74	.125	.078	.164	<.001
	75+	.246	.206	.297	<.001
Sex (ref: male)		.114	.069	.153	<.001
Living in the countryside		-.077	-.114	-.028	.001
Currently married		-.016	-.065	.031	.488
Education	None				
	Some	.024	-.018	.068	.257
	Primary & more	-.066	-.107	-.021	.004
Ethnicity	Dafin				
	Bwama	-.003	-.045	.040	.904
	Mossi	.010	-.028	.045	.663
	Other	.030	-.019	.094	.190
Wealth quintile	1				
	2	-.028	-.078	.024	.295
	3	-.058	-.110	-.004	.035
	4	-.077	-.126	-.021	.006
	5	-.101	-.154	-.042	.001
**Fasting Plasma Glucose > 100 mg/dl**		-.022	-.061	.019	.298

For peer review only

Supplementary Table 7: Linear regression model 7
(Hypertension grade II)

Dependent variable: Z-scored PHQ-9

		Model 7 (n=2970)			
		beta	CI low	CI high	p
Age	40-44	-	-	-	-
	45-49	.017	-.024	.058	.422
	50-54	.059	.018	.099	.004
	55-59	.100	.060	.139	<.001
	60-64	.141	.102	.179	<.001
	65-69	.188	.148	.224	<.001
	70-74	.163	.125	.199	<.001
	75+	.237	.197	.273	<.001
Sex (ref: male)		.118	.082	.154	<.001
Living in the countryside		-.047	-.078	-.008	.016
Currently married		-.028	-.072	.010	.143
Education	None				
	Some	.019	-.015	.054	.274
	Primary & more	-.033	-.070	.003	.072
Ethnicity	Dafin				
	Bwama	.020	-.017	.054	.309
	Mossi	-.001	-.033	.031	.946
	Other	.026	-.015	.081	.176
Wealth quintile	1				
	2	-.019	-.062	.024	.386
	3	-.055	-.099	-.011	.014
	4	-.066	-.109	-.021	.004
	5	-.086	-.133	-.039	<.001
**Hypertension grade II**		.014	-.020	.048	.427

For peer review only

Supplementary Table 8: Linear regression model 8 (Pre-diabetes)

Dependent variable: Z-scored PHQ-9

		Model 8 (n=2143)			
		beta	CI low	CI high	p
Age	40-44	-	-	-	-
	45-49	.005	-.042	.052	.838
	50-54	.050	.002	.093	.040
	55-59	.099	.050	.139	<.001
	60-64	.142	.096	.187	<.001
	65-69	.204	.155	.244	<.001
	70-74	.128	.082	.166	<.001
	75+	.240	.198	.287	<.001
Sex (ref: male)		.110	.066	.148	<.001
Living in the countryside		-.070	-.106	-.022	.003
Currently married		-.018	-.065	.028	.432
Education	None				
	Some	.026	-.015	.067	.216
	Primary & more	-.071	-.110	-.026	.001
Ethnicity	Dafin				
	Bwama	.000	-.041	.041	.999
	Mossi	.007	-.030	.042	.756
	Other	.038	-.008	.102	.097
Wealth quintile	1				
	2	-.024	-.073	.027	.368
	3	-.056	-.106	-.003	.040
	4	-.067	-.115	-.013	.014
	5	-.097	-.148	-.039	.001
**Pre-diabetes**		.005	-.034	.043	.819

For peer review only

Supplementary Table 9: Linear regression model 9 (Diabetes)

Dependent variable: Z-scored PHQ-9

		Model 9 (n=2043)			
		beta	CI low	CI high	p
Age	40-44	-	-	-	-
	45-49	.001	-.047	.048	.983
	50-54	.048	.000	.092	.051
	55-59	.104	.055	.146	<.001
	60-64	.143	.096	.188	<.001
	65-69	.191	.143	.235	<.001
	70-74	.123	.077	.163	<.001
	75+	.246	.204	.295	<.001
Sex (ref: male)		.112	.066	.150	<.001
Living in the countryside		-.081	-.118	-.031	.001
Currently married		-.017	-.065	.030	.474
Education	None				
	Some	.031	-.011	.075	.147
	Primary & more	-.066	-.105	-.020	.004
Ethnicity	Dafin				
	Bwama	-.010	-.051	.033	.672
	Mossi	.008	-.030	.043	.735
	Other	.031	-.018	.095	.179
Wealth quintile	1				
	2	-.033	-.082	.019	.227
	3	-.069	-.120	-.014	.013
	4	-.083	-.132	-.027	.003
	5	-.105	-.158	-.045	<.001
**Diabetes**		-.001	-.040	.039	.972

For peer review only

Supplementary Table 10: Linear regression model 10
(Self-reported hypertension)

Dependent variable: Z-scored PHQ-9

		Model 10 (n=2972)			
		beta	CI low	CI high	p
Age	40-44	-	-	-	-
	45-49	.016	-.025	.057	.434
	50-54	.056	.015	.095	.007
	55-59	.093	.053	.131	<.001
	60-64	.143	.103	.180	<.001
	65-69	.179	.140	.217	<.001
	70-74	.152	.115	.190	<.001
	75+	.235	.196	.271	<.001
Sex (ref: male)		.119	.083	.155	<.001
Living in the countryside		-.044	-.074	-.005	.027
Currently married		-.036	-.080	.003	.066
Education	None				
	Some	.017	-.017	.051	.329
	Primary & more	-.027	-.062	.010	.152
Ethnicity	Dafin				
	Bwama	.017	-.021	.051	.404
	Mossi	.000	-.032	.032	.999
	Other	.026	-.014	.081	.172
Wealth quintile	1				
	2	-.015	-.058	.029	.512
	3	-.050	-.093	-.005	.028
	4	-.060	-.104	-.015	.009
	5	-.087	-.133	-.039	<.001
**Self-reported hypertension**		.032	-.003	.066	.071

For peer review only

Supplementary Table 11: Linear regression model 11
(Self-reported diabetes)

Dependent variable: Z-scored PHQ-9

		Model 11 (n=2991)			
		beta	CI low	CI high	p
Age	40-44	-	-	-	-
	45-49	.013	-.028	.053	.541
	50-54	.058	.018	.097	.005
	55-59	.098	.057	.135	<.001
	60-64	.144	.104	.181	<.001
	65-69	.182	.143	.219	<.001
	70-74	.154	.117	.190	<.001
	75+	.233	.194	.269	<.001
Sex (ref: male)		.123	.086	.158	<.001
Living in the countryside		-.044	-.074	-.005	.024
Currently married		-.036	-.080	.002	.062
Education	None				
	Some	.020	-.014	.054	.250
	Primary & more	-.024	-.060	.012	.195
Ethnicity	Dafin				
	Bwama	.016	-.022	.050	.438
	Mossi	-.001	-.032	.031	.965
	Other	.023	-.018	.076	.230
Wealth quintile	1				
	2	-.015	-.058	.028	.500
	3	-.054	-.097	-.010	.016
	4	-.065	-.108	-.020	.004
	5	-.085	-.130	-.037	<.001
**Self-reported diabetes**		.004	-.029	.038	.809

For peer review only

Supplementary Table 12: Linear regression model 12
(Self-reported heart disease)

Dependent variable: Z-scored PHQ-9

		Model 12 (n=3001)			
		beta	CI low	CI high	p
Age	40-44	-	-	-	-
	45-49	.012	-.029	.052	.567
	50-54	.056	.016	.095	.006
	55-59	.094	.054	.132	<.001
	60-64	.143	.105	.181	<.001
	65-69	.181	.143	.219	<.001
	70-74	.161	.124	.197	<.001
	75+	.236	.197	.272	<.001
Sex (ref: male)		.112	.076	.148	<.001
Living in the countryside		-.047	-.077	-.008	.016
Currently married		-.029	-.072	.009	.128
Education	None				
	Some	.018	-.017	.052	.314
	Primary & more	-.026	-.062	.010	.159
Ethnicity	Dafin				
	Bwama	.016	-.021	.051	.410
	Mossi	-.002	-.033	.030	.931
	Other	.021	-.021	.074	.272
Wealth quintile	1				
	2	-.021	-.063	.022	.338
	3	-.058	-.102	-.015	.009
	4	-.071	-.115	-.027	.002
	5	-.092	-.138	-.045	<.001
**Self-reported heart disease**		.081	.047	.114	<.001

For peer review only

Supplementary Table 13: Linear regression model 13
(Self-reported stroke)

Dependent variable: Z-scored PHQ-9

		Model 13 (n=3026)			
		beta	CI low	CI high	p
Age	40-44	-	-	-	-
	45-49	.015	-.025	.056	.455
	50-54	.058	.018	.097	.004
	55-59	.094	.055	.133	<.001
	60-64	.143	.104	.181	<.001
	65-69	.185	.147	.222	<.001
	70-74	.162	.125	.198	<.001
	75+	.236	.197	.272	<.001
Sex (ref: male)		.122	.086	.157	<.001
Living in the countryside		-.049	-.079	-.010	.012
Currently married		-.031	-.074	.008	.110
Education	None				
	Some	.019	-.015	.053	.282
	Primary & more	-.027	-.062	.009	.147
Ethnicity	Dafin				
	Bwama	.019	-.018	.053	.344
	Mossi	-.001	-.032	.031	.960
	Other	.023	-.018	.076	.224
Wealth quintile	1				
	2	-.018	-.061	.024	.401
	3	-.055	-.098	-.011	.014
	4	-.065	-.109	-.021	.004
	5	-.087	-.134	-.040	<.001
**Self-reported stroke**		.038	.005	.071	.024

For peer review only

Supplementary Table 14: Linear regression model 14
(Sudden drooping)

Dependent variable: Z-scored PHQ-9

		Model 14 (n=3026)			
		beta	CI low	CI high	p
Age	40-44	-	-	-	-
	45-49	.013	-.027	.054	.519
	50-54	.054	.014	.093	.008
	55-59	.094	.055	.132	<.001
	60-64	.137	.099	.175	<.001
	65-69	.183	.144	.220	<.001
	70-74	.157	.120	.193	<.001
	75+	.235	.196	.271	<.001
Sex (ref: male)		.120	.084	.155	<.001
Living in the countryside		-.049	-.078	-.010	.012
Currently married		-.031	-.075	.006	.100
Education	None				
	Some	.019	-.015	.053	.284
	Primary & more	-.027	-.062	.009	.147
Ethnicity	Dafin				
	Bwama	.015	-.021	.049	.438
	Mossi	-.001	-.033	.030	.947
	Other	.022	-.019	.075	.241
Wealth quintile	1				
	2	-.017	-.059	.026	.446
	3	-.056	-.099	-.013	.012
	4	-.066	-.109	-.022	.003
	5	-.087	-.133	-.041	<.001
**Sudden drooping**		.090	.057	.123	<.001

For peer review only

Supplementary Table 15: Linear regression model 15
(Sudden numbness)

Dependent variable: Z-scored PHQ-9

		Model 15 (n=3026)			
		beta	CI low	CI high	p
Age	40-44	-	-	-	-
	45-49	.017	-.024	.057	.419
	50-54	.059	.019	.098	.004
	55-59	.095	.056	.133	<.001
	60-64	.135	.096	.172	<.001
	65-69	.178	.140	.215	<.001
	70-74	.155	.118	.190	<.001
	75+	.227	.189	.263	<.001
Sex (ref: male)		.117	.081	.151	<.001
Living in the countryside		-.051	-.080	-.012	.008
Currently married		-.034	-.078	.003	.071
Education	None				
	Some	.016	-.018	.049	.366
	Primary & more	-.026	-.062	.009	.147
Ethnicity	Dafin				
	Bwama	.017	-.019	.051	.380
	Mossi	.001	-.031	.032	.965
	Other	.020	-.022	.072	.291
Wealth quintile	1				
	2	-.019	-.061	.023	.378
	3	-.061	-.104	-.017	.006
	4	-.072	-.115	-.028	.001
	5	-.097	-.143	-.051	<.001
**Sudden numbness**		.136	.102	.168	<.001

For peer review only

Supplementary Table 16: Linear regression model 16 (Sudden slurring)

Dependent variable: Z-scored PHQ-9

		Model 16 (n=3026)			
		beta	CI low	CI high	p
Age	40-44	-	-	-	-
	45-49	.016	-.025	.055	.448
	50-54	.056	.017	.096	.005
	55-59	.093	.054	.131	<.001
	60-64	.134	.096	.172	<.001
	65-69	.175	.137	.212	<.001
	70-74	.155	.118	.190	<.001
	75+	.227	.189	.263	<.001
Sex (ref: male)		.117	.081	.151	<.001
Living in the countryside		-.052	-.081	-.013	.007
Currently married		-.037	-.081	.000	.050
Education	None				
	Some	.019	-.015	.053	.264
	Primary & more	-.029	-.064	.007	.113
Ethnicity	Dafin				
	Bwama	.017	-.019	.051	.379
	Mossi	.001	-.030	.032	.954
	Other	.017	-.026	.068	.374
Wealth quintile	1				
	2	-.022	-.064	.020	.312
	3	-.060	-.103	-.017	.006
	4	-.072	-.116	-.029	.001
	5	-.091	-.136	-.044	<.001
**Sudden slurring**		.144	.110	.176	<.001

For peer review only

Supplementary Table 17: Linear regression model 17
(Self-reported metabolic syndrome)

Dependent variable: Z-scored PHQ-9

		Model 17 (n=2948)			
		beta	CI low	CI high	p
Age	40-44	-	-	-	-
	45-49	.014	-.027	.055	.497
	50-54	.055	.014	.094	.008
	55-59	.092	.052	.130	<.001
	60-64	.141	.101	.178	<.001
	65-69	.174	.135	.212	<.001
	70-74	.151	.114	.188	<.001
	75+	.230	.190	.266	<.001
Sex (ref: male)		.117	.080	.152	<.001
Living in the countryside		-.039	-.070	.000	.049
Currently married		-.039	-.083	-.001	.045
Education	None				
	Some	.018	-.017	.052	.314
	Primary & more	-.029	-.064	.008	.124
Ethnicity	Dafin				
	Bwama	.015	-.022	.049	.460
	Mossi	-.001	-.033	.031	.956
	Other	.024	-.017	.079	.201
Wealth quintile	1				
	2	-.016	-.059	.027	.459
	3	-.051	-.095	-.007	.024
	4	-.066	-.110	-.021	.004
	5	-.091	-.137	-.042	<.001
**Self-reported metabolic syndrome**		.070	.035	.104	<.001

For peer review only

Supplementary Table 18: Descriptive statistics of the self-reported diagnosis of hypertension and diabetes within the whole sample

	Total	Female	Male
**Self-reported diagnosis of hypertension**	*n* = 3007<br>507 (17.1%)	*n* = 1513<br>294 (19.8%)	*n* = 1494<br>213 (14.4%)
**Self-reported diagnosis of diabetes**	*n* = 2993<br>70 (2.3%)	*n* = 1505<br>32 (2.1%)	*n* = 1488<br>38 (2.6%)

Supplementary Table 19: Descriptive statistics of the self-reported diagnosis of hypertension and diabetes within the affected* part of the sample

	Total	Female	Male
**Self-reported diagnosis of hypertension**	*n* = 1674<br>346 (21.0%)	*n* = 841<br>203 (24.6%)	*n* = 832<br>143 (17.3%)
**Self-reported diagnosis of diabetes**	*n* = 143<br>20 (14.0%)	*n* = 85<br>11 (13.0%)	*n* = 58<br>9 (15.5%)

*In case of Hypertension: Systolic BP >130 mmHg or Diastolic BP >80 mmHg
Diabetes: Fasting Glucose >126 mg/dl, random glucose >200 mg/dl or HbA1c >6.5%

Supplementary Table 20: Measuring the effect of a diagnosis of hypertension/diabetes given by a health care worker among hypertensive/diabetic part of the population.
Given in the table are the standardized coefficients of multiple linear regression of the z-scored PHQ-9 score on self-reported diagnosis of hypertension within the hypertensive population[a] and diagnosis of diabetes within the diabetic population[b].

	Standardized beta coefficient	p-value
**Ever been diagnosed with hypertension**	0.033	0.061
**Ever been diagnosed with diabetes**	-0.025	0.762

All variables used in separate models controlling for age, sex, gender, place of residence, education, ethnicity and wealth quintile.
[a] Systolic BP >130 mmHg or Diastolic BP >80 mmHg
[b] Fasting Glucose >126 mg/dl, random glucose >200 mg/dl or HbA1c >6.5%

**Supplementary Table 21:** Distribution of the z-scored PHQ-9 values					
		PHQ-9 value	CI lower	CI upper	2 Std. Dev.
m	40-44	-0,414	-0,493	-0,336	0,078
w	40-44	-0,160	-0,252	-0,067	0,092
m	45-49	-0,403	-0,496	-0,311	0,092
w	45-49	-0,087	-0,196	0,022	0,109
m	50-54	-0,259	-0,359	-0,158	0,100
w	50-54	0,025	-0,084	0,135	0,110
m	55-59	-0,135	-0,285	0,015	0,150
w	55-59	0,162	0,030	0,293	0,131
m	60-64	-0,016	-0,175	0,142	0,159
w	60-64	0,428	0,284	0,572	0,144
m	65-69	0,297	0,111	0,484	0,186
w	65-69	0,552	0,393	0,710	0,159
m	70-74	0,369	0,085	0,653	0,284
w	70-74	0,571	0,337	0,806	0,235
m	75+	0,881	0,553	1,210	0,328
w	75+	0,795	0,541	1,050	0,254

For peer review only

**Supplementary Table 22:** Prevalence of the metabolic syndrome				
		Proportion	CI lower	CI upper
m	40-44	0,082	0,054	0,118
w	40-44	0,120	0,083	0,166
m	45-49	0,150	0,110	0,197
w	45-49	0,183	0,133	0,242
m	50-54	0,103	0,064	0,155
w	50-54	0,210	0,158	0,271
m	55-59	0,101	0,059	0,159
w	55-59	0,244	0,183	0,315
m	60-64	0,140	0,079	0,224
w	60-64	0,190	0,130	0,263
m	65-69	0,082	0,034	0,162
w	65-69	0,274	0,195	0,364
m	70-74	0,097	0,036	0,199
w	70-74	0,273	0,177	0,386
m	75+	0,057	0,012	0,157
w	75+	0,282	0,186	0,395

For peer review only

**Supplementary Table 23:** Prevalence of a positive screening result for pre-diabetes				
		Proportion	CI lower	CI upper
m	40-44	0,413	0,351	0,476
w	40-44	0,378	0,315	0,444
m	45-49	0,416	0,351	0,483
w	45-49	0,468	0,395	0,542
m	50-54	0,503	0,423	0,583
w	50-54	0,503	0,430	0,576
m	55-59	0,392	0,308	0,482
w	55-59	0,532	0,451	0,611
m	60-64	0,442	0,328	0,559
w	60-64	0,425	0,338	0,516
m	65-69	0,444	0,319	0,575
w	65-69	0,473	0,377	0,570
m	70-74	0,481	0,340	0,624
w	70-74	0,515	0,389	0,640
m	75+	0,591	0,432	0,737
w	75+	0,515	0,390	0,638

For peer review only

Supplementary Table 24: Prevalence of a positive screening result for diabetes mellitus				
		Proportion	CI lower	CI upper
m	40-44	0,028	0,011	0,058
w	40-44	0,049	0,025	0,085
m	45-49	0,065	0,036	0,107
w	45-49	0,072	0,039	0,120
m	50-54	0,058	0,027	0,108
w	50-54	0,061	0,031	0,107
m	55-59	0,080	0,039	0,142
w	55-59	0,069	0,034	0,124
m	60-64	0,097	0,040	0,190
w	60-64	0,105	0,057	0,173
m	65-69	0,035	0,004	0,121
w	65-69	0,107	0,055	0,183
m	70-74	0,021	0,001	0,113
w	70-74	0,125	0,056	0,232
m	75+	0,119	0,040	0,256
w	75+	0,111	0,046	0,216

For peer review only

**Supplementary Table 25:** Prevalence of a positive screening result for hypertension gr. II				
		Proportion	CI lower	CI upper
m	40-44	0,182	0,144	0,225
w	40-44	0,169	0,128	0,217
m	45-49	0,227	0,182	0,277
w	45-49	0,222	0,172	0,279
m	50-54	0,235	0,181	0,297
w	50-54	0,274	0,220	0,334
m	55-59	0,306	0,241	0,378
w	55-59	0,294	0,232	0,362
m	60-64	0,344	0,261	0,434
w	60-64	0,362	0,291	0,438
m	65-69	0,355	0,265	0,454
w	65-69	0,407	0,325	0,493
m	70-74	0,333	0,227	0,454
w	70-74	0,380	0,281	0,488
m	75+	0,379	0,262	0,507
w	75+	0,442	0,345	0,543

For peer review only

BMJ Open

Depressive symptoms and cardiovascular disease: a population-based study of older adults in rural Burkina Faso

Journal:	*BMJ Open*
Manuscript ID	bmjopen-2020-038199.R2
Article Type:	Original research
Date Submitted by the Author:	03-Aug-2020

Complete List of Authors:	Brinkmann, Ben; Heidelberg University, Heidelberg Institute of Global Health<br>Payne, Collin F.; • School of Demography, The Australian National University, Canberra, Australia, School of Demography<br>Kohler, Iliana ; University of Pennsylvania, Population Studies Center (PSC) and Department of Sociology<br>Harling, Guy; University College London, Institute for Global Health; Harvard Center for Population and Development Studies<br>Davies, Justine ; University of Birmingham, Institute of Applied Health Research<br>Witham, Miles; AGE Research Group, NIHR Newcastle Biomedical Research Centre, Newcastle University; Newcastle Upon Tyne Hospitals NHS Foundation Trust<br>Siedner, Mark; Harvard Medical School, Department of Medicine, Massachusetts General Hospital<br>Sie, Ali; Centre de Recherche en Sante de Nouna<br>Bountogo, Mamadou; Centre de Recherche en Sante de Nouna<br>Ouermi, Lucienne; Centre de Recherche en Sante de Nouna<br>Coulibaly, Boubacar; Centre de Recherche en Sante de Nouna<br>Bärnighausen, Till ; Heidelberg University, Heidelberg Institute of Global Health; Africa Health Research Institute

**Primary Subject Heading**:	Public health
Secondary Subject Heading:	Public health, Mental health, Cardiovascular medicine, Epidemiology, Geriatric medicine
Keywords:	Depression & mood disorders < PSYCHIATRY, Hypertension < CARDIOLOGY, STROKE MEDICINE, PUBLIC HEALTH, EPIDEMIOLOGY, MENTAL HEALTH

SCHOLARONE™
Manuscripts

I, the Submitting Author has the right to grant and does grant on behalf of all authors of the Work (as defined in the below author licence), an exclusive licence and/or a non-exclusive licence for contributions from authors who are: i) UK Crown employees; ii) where BMJ has agreed a CC-BY licence shall apply, and/or iii) in accordance with the terms applicable for US Federal Government officers or employees acting as part of their official duties; on a worldwide, perpetual, irrevocable, royalty-free basis to BMJ Publishing Group Ltd ("BMJ") its licensees and where the relevant Journal is co-owned by BMJ to the co-owners of the Journal, to publish the Work in this journal and any other BMJ products and to exploit all rights, as set out in our licence.

The Submitting Author accepts and understands that any supply made under these terms is made by BMJ to the Submitting Author unless you are acting as an employee on behalf of your employer or a postgraduate student of an affiliated institution which is paying any applicable article publishing charge ("APC") for Open Access articles. Where the Submitting Author wishes to make the Work available on an Open Access basis (and intends to pay the relevant APC), the terms of reuse of such Open Access shall be governed by a Creative Commons licence – details of these licences and which Creative Commons licence will apply to this Work are set out in our licence referred to above.

Other than as permitted in any relevant BMJ Author's Self Archiving Policies, I confirm this Work has not been accepted for publication elsewhere, is not being considered for publication elsewhere and does not duplicate material already published. I confirm all authors consent to publication of this Work and authorise the granting of this licence.

For peer review only

Title:

Depressive symptoms and cardiovascular disease: a population-based study of older adults in rural Burkina Faso

Corresponding Author:

Ben Brinkmann

Im Neuenheimer Feld 130.3

69120 Heidelberg

Germany

ben.brinkmann@stud.uni.heidelberg.de

+49 1573 6194470

Authors:

Ben Brinkmann[1], mail: ben.brinkmann@stud.uni-heidelberg.de

Collin Payne, PhD[2], mail: collin.payne@anu.edu.au

Iliana V. Kohler, PhD[3], mail: iliana@pop.upenn.edu

Guy Harling, ScD[4,5,6,7], mail: g.harling@ucl.ac.uk

Justine I. Davies[8], mail: j.davies.6@bham.ac.uk

Miles D. Witham[9], mail: Miles.Witham@newcastle.ac.uk

Mark J. Siedner[10], mail: MSIEDNER@mgh.harvard.edu

Ali Sié, MD, PhD[11], mail: sieali.crsn@fasonet.bf

Mamadou Bountogo, MD, MSc[11], mail: drbountogo@yahoo.fr

Lucienne Ouermi, MD[11], mail: ouermil@yahoo.fr

Boubacar Coulibaly, MD[11], mail: boubacar@fasonet.bf

Till Bärnighausen, MD, ScD[1,4], mail: till.baernighausen@uni-heidelberg.de

Affiliations:

[1] Heidelberg Institute of Global Health, Heidelberg University, Germany.

[2] School of Demography, Australian National University, Canberra, Australian Capital Territory, Australia.

For peer review only

[3] Population Studies Center (PSC) and Department of Sociology, University of Pennsylvania, Philadelphia, PA, USA.

[4] Harvard Center for Population and Development Studies, Cambridge, MA, USA.

[5] Institute for Global Health, University College London, London, UK.

[6] Africa Health Research Institute, KwaZulu-Natal, South Africa

[7] MRC/WITS Rural Public Health and Health Transitions Research Unit (Agincourt), Faculty of Health Sciences, University of the Witwatersrand, Johannesburg, South Africa

[8] Institute of Applied Health Research, University of Birmingham, Birmingham, UK

[9] AGE Research Group, NIHR Newcastle Biomedical Research Centre, Newcastle University and Newcastle upon Tyne Hospitals NHS Trust, Newcastle, UK

[10] Department of Medicine, Massachusetts General Hospital, Harvard Medical School, Boston, MA, USA

[11] Centre de Recherche en Santé de Nouna, Nouna, Burkina Faso.

Word count:

4,692

For peer review only

ABSTRACT

Objectives: To contribute to the current understanding of depressive disorders in sub-Saharan African (SSA) countries by examining the association of depressive symptoms with cardiovascular and cardiometabolic conditions in a population-based study of middle-aged and older adults in rural Burkina Faso.

Setting: This study was conducted in the Nouna Health and Demographic Surveillance System (HDSS) in north-western Burkina Faso, in a mixed rural and small-town environment. The data were obtained between May and July 2018.

Participants: Consenting adults over 40 years of age (n=3,026).

Primary and secondary outcome measures: Depressive symptoms were assessed using the Patient Health Questionnaire depression module (PHQ-9). Chronic cardiometabolic conditions were assessed via a lipid panel and HbA1c measures from serum, alongside anthropometry and blood pressure measurements and a self-reported questionnaire. Multivariable linear regression was used to test the relationship between depressive symptoms and cardiovascular/cardiometabolic conditions, after controlling for sociodemographic factors.

Results: Depressive symptoms were not associated with the metabolic syndrome (standardized beta coefficient = 0.00 [95% confidence interval (CI): -0.04 to 0.03]), hypertension (beta = 0.01 [95% CI: -0.02 to 0.05]), diabetes mellitus (beta = 0.00 [95% CI: -0.04 to 0.04]) and past diagnosis of elevated blood pressure or blood sugar. Prior stroke diagnosis (beta = 0.04 [95% CI: 0.01 to 0.07]) or heart disease (beta = 0.08 [95% CI: 0.05 to 0.11]) was positively associated with the standardized PHQ-9 score, as were self-reported stroke symptoms.

Conclusion: Objectively measured cardiometabolic conditions had no significant association with depressive symptoms in an older, poor, rural SSA population, in contrast to observations in high

income countries (HIC). However, consequences of cardiovascular disease such as stroke and heart attack were associated with depressive symptoms in older adults in Burkina Faso.

For peer review only

STRENGTHS TO THIS STUDY

- This study was large and population-based with a randomly selected sample, in contrast to the selective nature of prior studies about depression in Burkina Faso.
- The use of biomarker testing for cardiovascular health and the PHQ-9 depression screening instrument that has been validated in primary care settings in SSA, provides internationally comparable data.

LIMITATIONS TO THIS STUDY

- The oral translation of the study questionnaire into local languages makes it vulnerable to differences in expression during interviews.
- The cross-sectional design of the questionnaire limits our ability to determine temporal ordering of and causal links between cardiovascular and mental health events.
- We were not able to clinically confirm diagnoses of hypertension, diabetes and depression in study participants.

For peer review only

INTRODUCTION

The decline in communicable diseases and the ageing of populations worldwide, including low-income countries, means that cardiovascular disease (CVD) is now the leading cause of mortality worldwide, accounting for an estimated 31% of all deaths [1]. The leading cause of morbidity in the Global Burden of Disease study 2017, however, was depressive disorders [2].

In high-income contexts, CVD risk factors as summarized by the metabolic syndrome – obesity, dyslipidemia, hypertension and diabetes mellitus – are bidirectionally associated with depressive disorders [3-6]. People with depression are more likely to smoke tobacco, eat an unhealthy diet, be physically inactive and use alcohol to excess, which makes them more vulnerable to develop the metabolic syndrome [7]. Elevated activity of the hypothalamus-pituitary adrenal (HPA) axis caused by chronic stress in people with depression increases the risk for obesity, dyslipidemia and diabetes [8,9]. Alterations in the autonomic nervous system found in people with depression [10] contribute to hypertension and increased insulin resistance. Other related factors adding to the hypothesis of a shared pathophysiology are over-activated inflammatory pathways, oxidative stress, endothelial dysfunction and hormone disequilibrium [5,11-13].

CVDs such as ischemic heart disease and cerebrovascular stroke are associated with subsequent depression. While depression increases the risk of heart attack and stroke [14,15], both are also associated with higher levels of later depression [16-18]. Post-stroke depression might be a result of the brain damage from the stroke but can also be a psychological reaction to the stroke illness and to its disabling consequences. Risk factors for depressive symptoms after stroke include stroke severity, disability after stroke, cognitive impairment, lack of social support and genetic predisposition [19,20]. Furthermore, depression worsens the outcomes of stroke [20] and myocardial infarction [21], increasing mortality and worsening long-term functional results.

In recent years, a small but growing body of evidence from sub-Saharan Africa (SSA) suggests that associations between CVD, diabetes and depression seen in HIC may not generalize to lower-income settings [22,23]. In addition, it has been found that the age pattern for depressive

For peer review only

disorders differs substantially from high-income countries. Depression prevalence in high-income countries peaks around age 60 and declines in the following decades, resulting in an inverted U-shape [24]. In contrast, in low-income contexts the prevalence of depressive symptoms appears to increase consistently with age [23,25].

In this paper, we seek to contribute to the current understanding of depressive disorders in sub-Saharan African countries by using newly collected data from Burkina Faso. Specifically, we aim to examine the potential associations of depressive symptoms with the metabolic syndrome, hypertension, diabetes mellitus and self-reported chronic conditions and stroke symptoms as well as the age pattern of depressive symptoms. Depression and mental disorders more generally are understudied in Burkina Faso and the evidence about the relationships with CVDs is inconclusive. Two recent studies examined the prevalence of depressive symptoms within pre-selected populations, Yameogo et al. [26] focused on hypertensive outpatients and Napon et al. [27] investigated depressive symptoms among people who had a stroke. Duthé et al. [28] worked with a sample of urban adults in the capital city of Ouagadougou, finding an association between broadly defined self-reported chronic conditions and depressive symptoms. Very recently, Ouédraogo et al. [29] conducted a population-based study determining the prevalence of depressive symptoms in Burkina Faso for the first time.

In contrast to these previous studies, our work uses a large population-based sample in a mixed rural and small-town environment among a population of adults over 40 years of age. This allows us to examine the possible associations of depressive symptoms and CVD in older adults with less selection bias. It is of great importance to gather more information about the relationships between depressive symptoms and other diseases' morbidity in this SSA context, as it can provide novel ideas and solutions to better serve the healthcare needs of these populations.

For peer review only

METHODS

Sample

This population-based study was conducted in the Nouna Health and Demographic Surveillance System (HDSS) in north-western Burkina Faso, which is run by the Centre de Recherche en Santé de Nouna (CRSN) [30]. This HDSS is located in a rural region consisting of 58 villages centered around the town of Nouna. In 2015 there were about 107,000 individuals within the area, living in approximately 15,000 households. Of these individuals, approximately 18,000 were over the age of 40; we selected 3,998 of these individuals in a stratified two-stage cluster random sampling approach. We expected 25% non-response due to mortality, inadequate mobility or people rejecting to participate, with an aimed 3,000 responses. Our first-stage sampling unit was at the level of villages and the seven sectors of Nouna town. Where there were fewer than 50 individuals aged over 40 (six villages in the 2015 census), we included all of them in our sample. We then took the remaining sample in equal proportions from all remaining villages and sectors, selecting at random within village/sectors.

Data collection

Between May and July 2018 all sampled individuals were invited to participate at their homes. Consenting participants completed a questionnaire on socioeconomic and demographic characteristics, their physical, cognitive and mental health, and access to and usage of health care. Field workers were trained to administer the questionnaire, using French as the main language. Translations from French to the local dialect Djula were done within the training module and clearly communicated to all field workers verbally. Written Djula literacy is very limited in this setting, necessitating verbal rather than written translation.

Indicators of physical health such as weight, height and blood pressure were measured by study staff. Blood pressure (BP) was measured using Omron Series 7 portable blood pressure

For peer review only

machines (Omron Healthcare, Kyoto, Japan). After 15 minutes of rest, three measurements were taken in the left arm of the seated respondents with 5 minutes between each measurement. The mean of the second and third measurement was calculated and used in analyses. A blood sample was collected to measure glycated hemoglobin (HbA1c), triglycerides, high density lipoprotein (HDL), and total cholesterol. To measure blood glucose levels, a point-of-care finger-prick test was carried out. The participants were asked when they last consumed food or beverages to determine whether the glucose values were fasting or non-fasting. All blood collection as well as finger-prick tests were conducted by trained and certified phlebotomists.

Outcome Variable

To measure depressive symptoms, the Patient Health Questionnaire depression module (PHQ-9) was used. The PHQ-9 is an instrument designed for primary care, either to make a probable diagnosis of major depressive disorder (MDD) or to continuously measure depressive symptoms with a score between 0 and 27 [31]. Higher scores represent more severe depressive symptoms. The scale consists of 9 items, with participants asked if they experienced a range of depressive symptoms in the past two weeks, including: (1) little interest or pleasure in doing things; (2) feeling down, depressed, or hopeless; (3) trouble falling or staying asleep, or sleeping too much; (4) feeling tired or having little energy; (5) poor appetite or overeating; (6) feeling bad about yourself; (7) trouble concentrating on things; (8) moving or speaking so slowly/rapidly that other people could have noticed; and (9) thoughts that you would be better off dead of or hurting yourself in some way. The response categories are: (0) not at all, (1) several days, (2) more than half of the days, (3) nearly every day.

Because PHQ-9 cut-points for diagnosing MDD in the sub-Saharan African context remain unclear [32], we use the PHQ-9 as a continuous score to reflect the amount of depressive symptoms an individual experienced in the past two weeks prior to the questioning. We emphasize that the score does not equal a diagnosis of depression. This linear specification avoids a binary categorization and allows subthreshold depression to be evaluated in our

For peer review only

analyses. Subthreshold depression is characterized by clinically relevant symptomatology, and while less severe than MDD, its high prevalence can generate greater economic burden – in higher-income settings it is responsible for more doctor-patient consultations than MDD [33,34]. To account for potential interviewer effects, we calculated Z-scores for the PHQ-9 results within interviewer before analysis (Supplementary Figures 1 and 2).

Explanatory Variables

Based on the Harmonized Joint Scientific Statement (Harmonized) criteria [35], we defined someone as having the metabolic syndrome if they met at least three of five conditions: (1) body mass index (BMI) ≥ 30 kg/m^2; (2) mean systolic BP ≥ 130 mmHg or a mean diastolic BP ≥ 85 mmHg; (3) triglycerides ≥ 150 mg/dl; (4) HDL-cholesterol < 40 mg/dl in men, < 50 mg/dl in women; and (5) fasting blood glucose ≥ 100 mg/dl.

We also considered several individual chronic conditions as explanatory variables: having a positive screening result for hypertension grade II (i.e., BP levels of a mean systolic BP ≥ 140 mmHg or a mean diastolic BP ≥ 90 mmHg) [36]; having a positive screening result for prediabetes, defined as a fasting blood glucose value between 100 and 126 mg/dl and/or an HbA1c value between 5.7 and 6.5 %, based on the classification criteria of the American Diabetes Associations [37]; having a positive screening result for diabetes mellitus, defined as a fasting blood glucose value ≥ 126 mg/dl or an HbA1c value ≥ 6.5 %.

In the remainder of this article, we define 'hypertension' as a positive screening result for hypertension, rather than the clinically diagnosed condition (i.e., by a medically trained health care provider) according to country specific requirements. The same accounts for 'diabetes mellitus' and the 'metabolic syndrome'

Last, we used self-reported measures of chronic health conditions. These included whether the respondent reported ever being diagnosed with: elevated BP; elevated blood sugar; a heart

For peer review only

attack; a stroke; or had ever experienced any of three stroke symptoms: sudden drooping of one side of the face; sudden numbness, weakness or dead feeling on one half of the body; sudden difficulty speaking or slurring of speech. The stroke symptoms were selected according to the FAST ("Face", "Arm", "Speech", "Time") system, which was created by the Brain Attack Coalition based on the Cincinnati Pre-Hospital Stroke Scale in 1999 [38]. We also created a 'self-reported metabolic syndrome' variable, defined as reporting any of the first four self-reported diagnoses.

The wealth index was constructed using polychoric principal components analysis (PCA) methods as described by Kolenikov and Angeles [39]. This method is similar to the Filmer and Pritchett [40] PCA method commonly used by Demographic and Household Surveys [41], but incorporates continuous variables and a lack of ownership into wealth index quantification. Variables in the final calculation included, among others: main source of water, toilet type, number of bedrooms, number of cows, horses, and other animals, hectares of land, electricity, cell phone, and bank account.

Statistical Analysis

Baseline descriptive statistics were obtained and means calculated. Confidence intervals for binomial variables were calculated using the Clopper-Pearson intervals. Multivariable linear regression was conducted to see how physical morbidity measures were associated with depressive symptoms. The standardized beta coefficients that are used to express the degree as to which morbidity variables are associated with the PHQ-9 score represent the percent value in which the probability of a yes/no event changes for every one standard deviation change in the PHQ-9 score. All models were adjusted for demographic and socioeconomic characteristics of the respondents, including age (in 5-year groups), sex, place of residence (rural/semi-rural), marital status (not-married/married), education (none/some, but none completed/primary completed or more), ethnicity (Dafin, Mossi, Bwama, other) and a wealth index. We standardized all coefficients so that they represented the standard deviations of change in the PHQ-9 score

For peer review only

associated with a one-unit change in morbidity variable. Missing values were excluded listwise from analyses. All analyses were conducted using SPSS v25 (IBM, New York, USA).

Ethics

This study received ethics approval from the national health ethics committee in Ouagadougou (#2018-5-053), the CRSN institutional ethics committee in Nouna (#2018-04) and the ethics committee of the medical faculty of the Ruprecht-Karls-Universität Heidelberg (#S-120/2018).

Patients and Public Involvement

Patients and the public were not involved in the design, or conduct, or reporting, or dissemination plans of our research.

RESULTS

Summary statistics for the sample used in the present analysis are presented in Table 1. Of the 3,998 sampled individuals, 3,026 (75.7%) participants were found, consented and completed all questions from the PHQ-9 depression module and were included in the analysis. 1523 (50.3%) of the respondents were women, 1503 men. Women were older on average (mean: 55.5 years, standard deviation (SD): 11.2) than men (mean: 53.1 years, SD: 10.6). They were also more likely to be without formal schooling (90.7%) than men (78.1%), less likely to be married (55.3%, men: 84.7%) and had a higher mean BMI (22.5, SD: 5.0; men: 21.8, SD: 3.7). Hypertension, diabetes and hypertriglyceridemia all were equally distributed between both sexes. Elevated blood pressure was highly prevalent in the sample: 27.3% screened positive for grade II hypertension. The prevalence of diabetes in this population was relatively low: 6.8%. Among the

For peer review only

women, 70.8% had an HDL-cholesterol-level below the cut-point to fulfil the criteria for the metabolic syndrome, while only 45.7% of the men had an HDL-cholesterol below the cutoff.

Table 1: Sample characteristics

	**Total (%)** ***N* = 3026 (100)**	**Female (%)** ***n* = 1523 (50.3)**	**Male (%)** ***n* = 1503 (49.7)**
Age group (y)			
40-44	685 (22.6%)	300 (19.7%)	385 (25.6%)
45-49	579 (19.1%)	250 (16.4%)	329 (21.9%)
50-54	478 (15.8%)	252 (16.5%)	226 (15.0%)
55-59	393 (13.0%)	202 (13.3%)	191 (12.7%)
60-64	304 (10.0%)	178 (11.7%)	126 (8.4%)
65-69	249 (8.2%)	142 (9.3%)	107 (7.1%)
70-74	166 (5.5%)	94 (6.2%)	72 (4.8%)
≥75	172 (5.7%)	105 (6.9%)	67 (4.5%)
Place of residence			
Nouna	838 (27.7%)	452 (29.7%)	386 (25.7%)
Village	2188 (72.3%)	1071 (70.3%)	1117 (74.3%)
Ethnicity			
Dafin	1176 (38.9%)	575 (37.8%)	601 (40.0%)
Bwama	927 (30.6%)	490 (32.2%)	437 (29.1%)
Mossi	403 (13.3%)	199 (13.1%)	204 (13.6%)
Other[a]	520 (17.1%)	259 (16.9%)	261 (17.3%)
Education			
No formal schooling	2555 (84.4%)	1381 (90.7%)	1174 (78.1%)
Some schooling, but none completed	253 (8.4%)	79 (5.2%)	174 (11.6%)
Primary schooling completed and more	218 (7.2%)	63 (4.1%)	155 (10.3%)
Currently married	2115 (69.9%)	842 (55.3%)	1273 (84.7%)
Body mass index[b]			
<18.5	498 (16.8%)	268 (17.8%)	230 (15.6%)
18.5-24.9	1884 (63.4%)	875 (58.3%)	1009 (68.8%)
25.0-29.9	441 (14.8%)	249 (16.6%)	192 (13.1%)
≥30.0	148 (5.0%)	110 (7.3%)	38 (2.6%)
Hypertension[c]			
Grade I	1674 (56.3%)	841 (56.0%)	833 (56.7%)
Grade II	812 (27.3%)	433 (28.8%)	379 (25.8%)
Diabetes mellitus[d]			
Prediabetes	974 (45.4%)	530 (46.6%)	444 (44.1%)
Diabetes	139 (6.8%)	84 (7.7%)	55 (5.7%)
Hypertriglyceridemia[e]	335 (11.6%)	177 (12.0%)	158 (11.2%)
Low HDL[f]-cholesterol[g]	1366 (58.6%)	849 (70.8%)	517 (45.7%)
Metabolic Syndrome[h]	394 (15.6%)	260 (20.4%)	134 (10.7%)
Self-reported conditions			
Elevated blood pressure	507 (17.1%)	294 (19.8%)	213 (14.4%)
Elevated blood sugar	70 (2.3%)	32 (2.1%)	38 (2.6%)
Heart disease	166 (5.5%)	116 (7.7%)	50 (3.3%)
Stroke	40 (1.3%)	16 (1.1%)	24 (1.6%)
Self-reported stroke symptoms			
Sudden drooping	58 (1.9%)	33 (2.2%)	25 (1.7%)
Sudden numbness	240 (7.9%)	133 (8.7%)	107 (7.1%)
Sudden slurring	159 (5.3%)	88 (5.8%)	71 (4.7%)

For peer review only - http://bmjopen.bmj.com/site/about/guidelines.xhtml

Self-reported metabolic syndrome[i]	688 (23.3%)	396 (26.8%)	292 (19.9%)

[a] Peulh, Samo, Other. [b] Calculated by weight divided by squared height in meters (kg/m^2), 55 missing values due to missing height/weight measurements. [c] Hypertension grade I: mean systolic blood pressure (BP) ≥ 130 mmHg or mean diastolic BP ≥ 85 mmHg, hypertension grade II: mean systolic BP ≥ 140 mmHg or mean diastolic BP ≥ 90 mmHg. 55 missing values due to missing BP measurements. [d] Prediabetes: fasting blood glucose (FBG) value between 100 and 126 mg/dl and/or an HbA1c value between 5.7 and 6.5%, 882 missing values due to missing HbA1c measurements and non-fasting results that were excluded. Diabetes: FBG value ≥ 126 mg/dl or an HbA1c value ≥ 6.5 %, 982 missing values due to missing HbA1c measurements and non-fasting results that were excluded. [e] Hypertriglyceridemia: Triglycerides ≥150 mg/dl, 135 missing values due to missing Triglyceride measurements. [f] high-density-lipoprotein-cholesterol [g] Low HDL-cholesterol: <40 mg/dl in females, <50 mg/dl in males, 696 missing values due to missing HDL-cholesterol measurements. [h] 502Metabolic syndrome: three of five conditions: (1) body mass index (BMI) ≥ 30 kg/m^2; (2) mean systolic BP ≥ 130 mmHg or mean diastolic BP ≥ 85 mmHg; (3) triglycerides ≥ 150 mg/dl; (4) HDL-cholesterol < 40 mg/dl in men, < 50 mg/dl in women; or (5) FBG ≥ 100 mg/dl. missing values due to missing height, weight, HDL-cholesterol, and Triglyceride measurements as well as non-fasting glucose measurements. [i] 78 missing values.

Age patterns of depressive symptoms

As Figure 1 shows, the absolute mean values of the z-scored PHQ-9 scores rose continually with age in both men and women. Considering only men, the mean PHQ-9 value for the youngest group of respondents, the 40-44 years old, was -0.41 [95% confidence interval (CI): -0.49 to -0.34]. This value rose constantly up to 0.88 [95% CI: 0.55 to 1.2] within the oldest age group of over 75-years-old. PHQ-9 scores were generally higher among women corresponding to higher levels of depressive symptoms that were also positively correlated with age. The mean value for women in the youngest age group was -0,16 [95% CI: -0.25 to -0.07], rising to 0,80 [95% CI: 0.54 to 1.05] for women in the oldest group. Underlying data can be found in Supplementary Materials (Supplementary Table 1).

Age patterns of the metabolic syndrome, diabetes mellitus, and hypertension

Figure 2 shows that the prevalence of the metabolic syndrome increased in a statistically significant way with age for women – it increased from a mean of 12.0% [95% CI: 8.3% to 16.6%) within 40-44-year-olds to 28.2% [95% CI: 18.6% to 39.5%) within the 75+ age group. Surprisingly, the prevalence rate for men declined slightly with age, going from 8.2% [95% CI: 5.4% to 11.8%) to 5.7% [95% CI: 0.0% to 15.7%) – though this difference was not statistically significant. A small

For peer review only

increase in the prevalence of prediabetes and diabetes was observed with increasing age, but this difference was not statistically significant. While an increasing prevalence with age could not be shown for diabetes mellitus, we did find a positive association between age and hypertension. Both men and women showed a rising prevalence of elevated blood pressure with increasing age. Underlying data can be found in Supplementary Materials (Supplementary Tables 2-5).

Association of depressive symptoms with the metabolic syndrome, its components, hypertension and diabetes mellitus

The first panel of Table 2 and Figure 3 show the standardized coefficients from simple linear regressions of the z-scored PHQ-9 score on the metabolic syndrome, its components, hypertension and diabetes. In all models, higher age and being female were strongly associated with a higher PHQ-9 score. Having the metabolic syndrome, being positively screened for hypertension, prediabetes or diabetes was not associated with a higher PHQ-9 score. Neither was any of the single components of the metabolic syndrome.

Table 2: Standardized beta coefficients derived from separate multivariable linear regression models investigating the relationship between cardiometabolic and cardiovascular conditions and the z-scored PHQ-9 results.

	**Standardized beta coefficient**	**95% confidence interval**	**p-value**
Metabolic syndrome	-0.004	[-0.040 to 0.033]	0.847
Obesity	0.007	[-0.028 to 0.042]	0.680
Hypertriglyceridemia	-0.006	[-0.040 to 0.029]	0.742
Reduced HDL-cholesterol	-0.027	[-0.067 to 0.012]	0.177
Hypertension grade I	0.028	[-0.007 to 0.062]	0.113
FPG > 100	-0.022	[-0.061 to 0.019]	0.298
Hypertension grade II	0.014	[-0.020 to 0.048]	0.427
Prediabetes	0.005	[-0.034 to 0.043]	0.819
Diabetes	-0.001	[-0.040 to 0.039]	0.972
Self-reported conditions			
Hypertension	0.032	[-0.003 to 0.066]	0.071
Diabetes	0.004	[-0.029 to 0.038]	0.809
Heart disease	0.081	[0.047 to 0.114]	<0.001
Stroke	0.038	[0.005 to 0.071]	0.024
Self-reported stroke symptoms			
Sudden drooping	0.090	[0.057 to 0.123]	<0.001
Sudden numbness	0.136	[0.102 to 0.168]	<0.001
Sudden slurring	0.144	[0.110 to 0.176]	<0.001
Self-reported metabolic syndrome	0.070	[0.035 to 0.104]	<0.001

For peer review only

Each standardized coefficient arises from a separate model controlling for age, sex, gender, place of residence, education, ethnicity and wealth quintile. The separate models can be found in Supplementary Materials (Supplementary Tables 6-22).

Association of depressive symptoms with self-reported conditions and stroke symptoms

Figure 3 shows that having previously been diagnosed by a health care worker with elevated blood pressure or elevated blood sugar was not significantly associated with the PHQ-9 score, whereas the models did show a positive association for ever having been diagnosed with a stroke (standardized beta coefficient (hereafter 'beta') = 0.04 [95% CI: 0.01 to 0.07]) or heart disease (beta = 0.08 [95% CI: 0.05 to 0.11]). An even stronger association could be shown for reporting to ever having had a sudden drooping of one side of the face (beta = 0.09 [95% CI: 0.06 to 0.12]), a sudden numbness weakness or dead feeling on one half of the body (beta = 0.14 [95% CI: 0.10 to 0.17]) and a sudden slurring of speech (beta = 0.14 [95% CI: 0.11 to 0.18]).

The 'self-reported metabolic syndrome' with one or more out of four self-reported health items present was associated positively with the PHQ-9 score (beta = 0.07 [95% CI: 0.04 to 0.10]). The results did not change substantially when running the regression models for the self-reported health items within the parts of the population who were screened positively for the respective condition, e.g. the model with self-reported elevated blood pressure within the population who were measured as hypertensive (Supplementary Tables 23-25).

DISCUSSION

Depressive symptoms were not associated with the metabolic syndrome, hypertension, diabetes mellitus and past diagnosis of elevated blood pressure or blood sugar. Prior stroke diagnosis or heart disease were positively associated with the standardized PHQ-9 score, as were self-reported stroke symptoms. Additionally, the prevalence of depressive symptoms rose

For peer review only

substantially with increasing age for both men and women, confirming similar findings from recent studies investigating depression in SSA and standing in contrast to findings from high income countries. The findings indicate that in a setting where cardiovascular conditions are not the dominant health issue, non-symptomatic conditions like low-grade hypertension do not have detrimental effects on people's mental health.

Chronic conditions and depressive symptoms

The findings suggest that the metabolic syndrome, hypertension and diabetes have no significant association with depressive symptoms in our sample. This situation is congruent with recent studies from SSA investigating the relationship between CVD and depression. Gelaye et al. [42] did not find an association between depression and the metabolic syndrome or diabetes in a sample of Ethiopian adults, Geldsetzer et al. [22] showed that there was no association between depression and hypertension, diabetes and obesity in a South African sample. In contrast to these results from SSA, relationships between CVD and depression have been widely reported in HIC [3-6,43]. In SSA, NCD including CVD are only recently emerging as a public health concern, while infectious diseases are still the most important cause of death [44]. People and health systems are more attuned to seeing and treating acute infections and maternal health problems than CVD, which might lead to a lower degree of understanding of what it means to have CVD – hence protecting the mental health of people in SSA. This hypothesis will need to be tested in future studies. On the other hand, it is known that individuals with a higher socioeconomic status (SES) can afford higher health care costs than those with a lower SES, for whom it is a greater burden to pay for their health. Fiske et al. [17] found that unaffordable health care costs for individuals affected by CVD may have a negative effect on their mental health.

CVD risk factors are asymptomatic, especially in their early stages, which might reduce the negative effects of CVD on mental health. Research in HIC has shown that there are biological factors like chronic inflammation, vascular endothelial dysfunction, neuroendocrine disequilibrium (particularly of the pituitary-adrenal axis) and increased platelet activation that can cause

For peer review only

depression in people with CVD and viceversa [45,46]. It remains unclear in the sub-Saharan African context if these biomedical processes are similarly important in driving an association between depressive symptoms and CVD; further research is needed to investigate this question. Multimorbidity might be another factor linking CVD and depression in HIC [47]. According to a recent study conducted in the same HDSS as this study, multimorbidity is highly prevalent in this sample [48]. Whether experiencing other NCDs in addition to CVD – for instance cancer, COPD, musculoskeletal disorders and other mental illnesses – has an effect on the relationship between depressive symptoms and CVD has not been examined and could be subject to further investigations. Use of antidepressants is linked to a higher risk of obesity, diabetes, and CVD [49] and could thus act as a confounding factor in studies in HIC investigating the association between depressive symptoms and cardiometabolic risk. In Burkina Faso the availability of specialized care is poor [50] and so is the availability of antidepressants, which makes it less likely for Burkinabé to experience the adverse outcomes of antidepressants.

Consequences of CVD

Interestingly, our findings suggest that having experienced the consequences of a stroke as well as having ever been diagnosed with a stroke or heart disease is associated with higher PHQ-9 results. These associations are in congruence with the situation in high-income countries, where especially ischemic events like heart attacks and strokes due to CVD are associated with depression [45]. Ischemic events may lead to physical dysfunction, impairing the ability to work in physical jobs that are important for the social and economic livelihood in a rural SSA environment. Social insecurity and disintegration resulting from these limitations are substantial risk factors to develop depressive symptoms. According to Schatz and Seeley [51], older adults in SSA strongly link their physical functionality to the concept of seeing themselves as independent individuals. With declines in physical functioning, their perceived independence deteriorates, leaving individuals as increasingly dependent and at risk of depression. Limited economic resources hinder the ability of older adults in rural areas to cope with stressors like disability after stroke, and also act to limit access to an already weak health system. This paucity of diagnosis

For peer review only

and treatment options also acts as part of a vicious cycle, as it may lead to substantial delays in accessing the health care system [52]. These barriers to mental health care lead to a lack of knowledge and awareness of an individual's own physical and mental health, making it much less likely to engage in protective behaviors such as regular physical activity, avoiding stimulants and drugs, engaging in stabile relationships or adjusting sleep rhythms and diet[53].

Sociodemographics

Women had a higher prevalence of depressive symptoms than men in our sample, showing that it will be especially important to target women in future interventions. With increasing age, the prevalence of depressive symptoms rose substantially for both men and women, confirming similar findings from recent studies investigating depression in SSA [22,23]. Mechanisms that protect older populations in HIC include an increasing and positive influence of religiosity, gathered wisdom and life experience, higher economic resources, intensified social engagement and socio-emotional selectivity [17,18]. Examining whether these resilience mechanisms apply to the SSA context should be a focus for future research.

Implications

The findings suggest that improving care for measurable CVD conditions – metabolic syndrome, hypertension and diabetes – may have additional benefits for improving depressive symptoms. Even though they might not be directly associated with depressive symptoms, these conditions increase the likelihood of thromboembolic events which are associated with worse mental health. When depression is detected in its early stages, it might also be possible to prevent a part of CVD burden by engaging in preventive diagnostics and treatment in a targeted part of the population. While it is highly prevalent and likely an important correlate and basis for other chronic conditions and care success, depression has been woefully neglected in the global health response. As

For peer review only

diagnostic and therapeutic options exist, this gap should be addressed by policymakers as soon as possible.

Strengths and limitations

Our study had several strengths. It was large and population-based with a randomly selected sample, in contrast to the selective nature of almost all prior studies about depression in Burkina Faso, with the notable exception of Ouédraogo et al. [29]. The use of both questionnaire and biomarker testing allowed objective measurement of chronic conditions, using internationally standardized values allowing comparison to studies elsewhere. Furthermore, the PHQ-9 instrument is locally applicable, being widely used in population-based studies and primary care settings in SSA [23,54-56]. Nevertheless, its translation into the local language, particularly the collaborative manner in which it was done, makes it vulnerable to differences in expression during interviews. While we have adjusted for differential understanding by standardizing scores at the interviewer level, cultural differences in expressing psychological states like sadness may still be important. The cross-sectional design of the questionnaire and the one-time sample do not make it possible to draw causal interpretations from the findings or to confirm diagnoses of hypertension, diabetes and depression. Finally, our sample of adults aged 40 and above is not directly comparable to the typically older cut-offs for aging studies; however given the life expectancy of Burkinabé people (60.8 years [57]) we believe that the respondents represent old individuals within this setting. Clearly our findings only apply to over-40s, and associations may differ in younger individuals.

Our analyses do not account for the potential that some individuals in our sample may be on medication to control a cardiovascular condition. Respondents who self-reported having hypertension, elevated blood sugar, hypercholesterolemia, or heart disease were asked a single combined question on whether they had received any treatment in the last two weeks. However, the definition of “treatment” was not specified any further, and therefore may represent treatments as varied as medication, physical exercise, dietary change, or traditional healing. Self-reported

For peer review only

rates of treatment were generally low, with only 11% of those with hypertension and 33% of those with self-reported hypertension reported having received treatment in the last two weeks. The numbers for elevated blood sugar were even lower: 8.6% of those with diabetes and 22.9% of those with self-reported elevated blood sugar said they had received treatment in the last two weeks. Our data showing low treatment rates are consistent with studies in SSA that document similar patterns for hypertension [58-61] and diabetes [62]. The non-specific nature of the available treatment variable means that any analyses using this variable would be nearly impossible to meaningfully interpret, and we therefore chose to omit self-reported treatment from our analyses.

Conclusion

In conclusion, we find that having the metabolic syndrome, hypertension or diabetes was not associated with the results of the PHQ-9 score among a group of older adults in a rural, sub-Saharan African setting. Interestingly, an association could be found between depressive symptoms and having experienced stroke symptoms as well as self-reported diagnose of stroke and heart disease. This might be due to the influence of disability after thromboembolic events on the psychological well-being of African adults seen in prior research. It may also be seen as a development foreshadowing an association of CVD and depression in this population, which cannot be seen yet because of lacking awareness, diagnosis and treatment options. Age was strongly associated with depressive symptoms, confirming the age patterns found in previous studies in SSA.

Acknowledgements

Funding for the CRSN Heidelberg Aging Study (CHAS) was provided by the Alexander von Humboldt Foundation through an Alexander von Humboldt Professor award to TB, which is funded by the German Federal Ministry of Education and Research. The funder has no role in the

For peer review only

design of the study and collection, analysis, and interpretation of data and in writing the manuscript of the study. MDW acknowledges support from NIHR Newcastle Biomedical Research Centre. GH is supported by a fellowship from the Wellcome Trust and Royal Society (210479/Z/18/Z). The funders had no role in study design, data collection and analysis, decision to publish, or preparation of the manuscript.

Conflict of Interest

None reported.

Data availability

Data are not publicly available as consent was not given by participants for data to be shared openly. This is in part because entire age cohorts of some villages are included in the dataset, potentially allowing for deductive disclosure with sufficient local information. For this reason, anonymized data is available from CHAS study data controllers only following signature of a data use agreement restricting onward transmission. Anyone wishing to replicate the analyses presented, or conduct further collaborative analyses using CHAS (which are welcomed and considered based on a letter of intent), should contact Dr Guy Harling (g.harling@ucl.ac.uk) in the first instance.

Contributors

TB and GH conceived and designed the overall CRSN CHAS study. CP, IK, JD, MW and MS contributed to the design of the CRSN CHAS household survey. BB, GH, AS, MB, LO and BC coordinated baseline data collection and preparation. BB conducted the analysis, and wrote, and revised the manuscript. CP and IK supervised the analysis, write up, and development of the

For peer review only

manuscript. All authors substantively reviewed manuscripts, inputted into revisions, and approved the final manuscript.

REFERENCES

1 WHO. Depression and Other Common Mental Disorders: Global Health Estimates. 2017.

2 James SL, Abate D, Abate KH, Abay SM, Abbafati C, Abbasi N, et al. Global, regional, and national incidence, prevalence, and years lived with disability for 354 diseases and injuries for 195 countries and territories, 1990–2017: a systematic analysis for the Global Burden of Disease Study 2017. The Lancet. 2018;392:1789-858.

3 Repousi N, Masana MF, Sanchez-Niubo A, Haro JM, Tyrovolas S. Depression and metabolic syndrome in the older population: A review of evidence. J Affect Disord. 2018;237:56-64.

4 Ghanei Gheshlagh R, Parizad N, Sayehmiri K. The Relationship Between Depression and Metabolic Syndrome: Systematic Review and Meta-Analysis Study. Iran Red Crescent Med J. 2016;18:e26523-e.

5 Marazziti D, Rutigliano G, Baroni S, Landi P, Dell'Osso L. Metabolic syndrome and major depression. CNS Spectrums. 2014;19:293-304.

6 Vaccarino V, McClure C, Johnson BD, Sheps DS, Bittner V, Rutledge T, et al. Depression, the metabolic syndrome and cardiovascular risk. Psychosom Med. 2008;70:40-8.

7 Stein DJ, Benjet C, Gureje O, Lund C, Scott KM, Poznyak V, et al. Integrating mental health with other non-communicable diseases. BMJ. 2019;364:l295.

8 Bao AM, Meynen G, Swaab DF. The stress system in depression and neurodegeneration: Focus on the human hypothalamus. Brain Research Reviews. 2008;57:531-53.

9 Björntorp P. Do stress reactions cause abdominal obesity and comorbidities? Obesity Reviews. 2001;2:73-86.

10 Carney RM, Freedland KE, Veith RC. Depression, the Autonomic Nervous System, and Coronary Heart Disease. Psychosomatic Medicine. 2005;67:S29-S33.

11 Golden SH. A review of the evidence for a neuroendocrine link between stress, depression and diabetes mellitus. Curr Diabetes Rev. 2007;3:252-9.

For peer review only

12 Carnethon MR, Biggs ML, Barzilay JI, Smith NL, Vaccarino V, Bertoni AG, et al. Longitudinal Association Between Depressive Symptoms and Incident Type 2 Diabetes Mellitus in Older Adults: The Cardiovascular Health Study. Archives of Internal Medicine. 2007;167:802-7.

13 Ford DE, Erlinger TP. Depression and C-Reactive Protein in US Adults: Data From the Third National Health and Nutrition Examination Survey. Archives of Internal Medicine. 2004;164:1010-4.

14 Pan A, Sun Q, Okereke OI, Rexrode KM, Hu FB. Depression and Risk of Stroke Morbidity and Mortality: A Meta-analysis and Systematic Review. JAMA. 2011;306:1241-9.

15 Skala JA, Freedland KE, Carney RM. Coronary Heart Disease and Depression: A Review of Recent Mechanistic Research. The Canadian Journal of Psychiatry. 2006;51:738-45.

16 Huang C-Q, Dong B-R, Lu Z-C, Yue J-R, Liu Q-X. Chronic diseases and risk for depression in old age: A meta-analysis of published literature. Ageing Research Reviews. 2010;9:131-41.

17 Fiske A, Wetherell JL, Gatz M. Depression in older adults. Annu Rev Clin Psychol. 2009;5:363-89.

18 Blazer DG, Hybels CF. Origins of depression in later life. Psychol Med. 2005;35:1241-52.

19 Robinson RG, Jorge RE. Post-Stroke Depression: A Review. American Journal of Psychiatry. 2015;173:221-31.

20 Ayerbe L, Ayis S, Wolfe CDA, Rudd AG. Natural history, predictors and outcomes of depression after stroke: systematic review and meta-analysis. British Journal of Psychiatry. 2013;202:14-21.

21 Lichtman Judith H, Froelicher Erika S, Blumenthal James A, Carney Robert M, Doering Lynn V, Frasure-Smith N, et al. Depression as a Risk Factor for Poor Prognosis Among Patients With Acute Coronary Syndrome: Systematic Review and Recommendations. Circulation. 2014;129:1350-69.

22 Geldsetzer P, Vaikath M, Wagner R, Rohr JK, Montana L, Gomez-Olive FX, et al. Depressive Symptoms and Their Relation to Age and Chronic Diseases Among Middle-Aged and Older Adults in Rural South Africa. J Gerontol A Biol Sci Med Sci. 2019;74:957-63.

23 Kohler IV, Payne CF, Bandawe C, Kohler HP. The Demography of Mental Health Among Mature Adults in a Low-Income, High-HIV-Prevalence Context. Demography. 2017;54:1529-58.

24 Blazer DG. Depression in late life: review and commentary. J Gerontol A Biol Sci Med Sci. 2003;58:249-65.

For peer review only

25 Thapa SB, Martinez P, Clausen T. Depression and its correlates in South Africa and Ghana among people aged 50 and above: Findings from the WHO Study on global AGEing and adult health. Journal of psychiatry. 2014;17:1000167.

26 Yameogo NV, Samadoulougou A, Kagambega LJ, Yameogo AA, Ilboudo E, Millogo G, et al. [Sleep disorders, anxiety and depressive symptoms and cardiovascular risk among black African hypertensive: cross-sectional study of 414 hypertensive followed as outpatients at the University Hospital of Ouagadougou (Burkina Faso)]. Pan Afr Med J. 2015;21:115.

27 Napon C, Kaboré A, Kaboré J. Post stroke depression in Burkina Faso. The Pan African medical journal. 2012;13:3-.

28 Duthe G, Rossier C, Bonnet D, Soura AB, Corker J. Mental health and urban living in sub-Saharan Africa: major depressive episodes among the urban poor in Ouagadougou, Burkina Faso. Popul Health Metr. 2016;14:18.

29 Ouédraogo A, Ouango JG, Karfo K, Goumbri P, Nanéma D, Sawadogo B. Prévalence des troubles mentaux en population générale au Burkina Faso. L'Encéphale. 2019;45:367-70.

30 Sie A, Louis VR, Gbangou A, Muller O, Niamba L, Stieglbauer G, et al. The Health and Demographic Surveillance System (HDSS) in Nouna, Burkina Faso, 1993-2007. Glob Health Action. 2010;3.

31 Kroenke K, Spitzer RL, Williams JB, Lowe B. The Patient Health Questionnaire Somatic, Anxiety, and Depressive Symptom Scales: a systematic review. Gen Hosp Psychiatry. 2010;32:345-59.

32 Sweetland AC, Belkin GS, Verdeli H. Measuring depression and anxiety in sub-saharan Africa. Depression and anxiety. 2014;31:223-32.

33 Cuijpers P, Vogelzangs N, Twisk J, Kleiboer A, Li J, Penninx BW. Differential mortality rates in major and subthreshold depression: meta-analysis of studies that measured both. British Journal of Psychiatry. 2013;202:22-7.

34 Johnson J, Weissman MM, Klerman GL. Service Utilization and Social Morbidity Associated With Depressive Symptoms in the Community. JAMA. 1992;267:1478-83.

35 Alberti KG, Eckel RH, Grundy SM, Zimmet PZ, Cleeman JI, Donato KA, et al. Harmonizing the metabolic syndrome: a joint interim statement of the International Diabetes Federation Task Force on Epidemiology and Prevention; National Heart, Lung, and Blood Institute; American

For peer review only

Heart Association; World Heart Federation; International Atherosclerosis Society; and International Association for the Study of Obesity. Circulation. 2009;120:1640-5.

36 Whelton PK, Carey RM. The 2017 American College of Cardiology/American Heart Association Clinical Practice Guideline for High Blood Pressure in Adults. JAMA Cardiol. 2018;3:352-3.

37 American Diabetes A. 2. Classification and Diagnosis of Diabetes: Standards of Medical Care in Diabetes-2018. Diabetes Care. 2018;41:S13-S27.

38 Kothari RU, Pancioli A, Liu T, Brott T, Broderick J. Cincinnati Prehospital Stroke Scale: Reproducibility and Validity. Annals of Emergency Medicine. 1999;33:373-8.

39 Kolenikov S, Angeles G. Socioeconomic Status Measurement With Discrete Proxy Variables: Is Principal Component Analysis A Reliable Answer? Review of Income and Wealth. 2009;55:128-65.

40 Filmer D, Pritchett LH. Estimating Wealth Effects Without Expenditure Data—Or Tears: An Application To Educational Enrollments In States Of India*. Demography. 2001;38:115-32.

41 Rutstein SO, Johnson K. The DHS wealth index. Calverton, Maryland, USA: ORC Macro, 2004.

42 Gelaye B, Williams MA, Lemma S, Berhane Y, Fann JR, Vander Stoep A, et al. Major depressive disorder and cardiometabolic disease risk among sub-Saharan African adults. Diabetes & metabolic syndrome. 2015;9:183-91.

43 Pan A, Keum N, Okereke OI, Sun Q, Kivimaki M, Rubin RR, et al. Bidirectional association between depression and metabolic syndrome: a systematic review and meta-analysis of epidemiological studies. Diabetes care. 2012;35:1171-80.

44 Institute for Health Metrics and Evaluation (IHME). GBD Compare Data Visualization. Seattle, WA: IHME, University of Washington. Available from http://vizhub.healthdata.org/gbd-compare. (Accessed 9th of November 2019). 2018.

45 Goldston K, Baillie AJ. Depression and coronary heart disease: A review of the epidemiological evidence, explanatory mechanisms and management approaches. Clinical Psychology Review. 2008;28:288-306.

46 Krishnan KR, Delong M, Kraemer H, Carney R, Spiegel D, Gordon C, et al. Comorbidity of depression with other medical diseases in the elderly. Biol Psychiatry. 2002;52:559-88.

For peer review only

47 Søgaard M, Skjøth F, Kjældgaard JN, Larsen TB, Hjortshøj SP, Riahi S. Atrial fibrillation in patients with severe mental disorders and the risk of stroke, fatal thromboembolic events and bleeding: a nationwide cohort study. BMJ open. 2017;7:e018209-e.

48 Odland ML, Payne C, Witham M, Siedner M, Bärnighausen T, Bountogo M, et al. The epidemiology of multimorbidity in conditions of extreme poverty: a population-based study of older adults in rural Burkina Faso. Manuscript submitted for publication. 2020.

49 Correll CU, Detraux J, De Lepeleire J, De Hert M. Effects of antipsychotics, antidepressants and mood stabilizers on risk for physical diseases in people with schizophrenia, depression and bipolar disorder. World Psychiatry. 2015;14:119-36.

50 Maiga DD, Eaton J. A survey of the mental healthcare systems in five Francophone countries in West Africa: Bénin, Burkina Faso, Côte d'Ivoire, Niger and Togo. International Psychiatry. 2014;11:69-72.

51 Schatz E, Seeley J. Gender, ageing and carework in East and Southern Africa: A review. Glob Public Health. 2015;10:1185-200.

52 Collins KA, Westra HA, Dozois DJ, Burns DD. Gaps in accessing treatment for anxiety and depression: challenges for the delivery of care. Clin Psychol Rev. 2004;24:583-616.

53 Sarris J, O'Neil A, Coulson CE, Schweitzer I, Berk M. Lifestyle medicine for depression. BMC psychiatry. 2014;14:107-.

54 Motlhatlhedi K, Setlhare V, Ganiyu AB, Firth JA. Association between depression in carers and malnutrition in children aged 6 months to 5 years. African Journal of Primary Health Care & Family Medicine. 2017;9:1-6.

55 L'Akoa R M, Noubiap JJ, Fang Y, Ntone FE, Kuaban C. Prevalence and correlates of depressive symptoms in HIV-positive patients: a cross-sectional study among newly diagnosed patients in Yaounde, Cameroon. BMC Psychiatry. 2013;13:228.

56 Bindt C, Appiah-Poku J, Te Bonle M, Schoppen S, Feldt T, Barkmann C, et al. Antepartum depression and anxiety associated with disability in African women: cross-sectional results from the CDS study in Ghana and Cote d'Ivoire. PLoS One. 2012;7:e48396.

57 United Nations, Department of Economic and Social Affairs, Population Division. World Population Prospects 2019, custom data acquired via website. Accessed 23rd March 2020. 2019.

For peer review only

58 Cappuccio Francesco P, Micah Frank B, Emmett L, Kerry Sally M, Antwi S, Martin-Peprah R, et al. Prevalence, Detection, Management, and Control of Hypertension in Ashanti, West Africa. Hypertension. 2004;43:1017-22.

59 Ataklte F, Erqou S, Kaptoge S, Taye B, Echouffo-Tcheugui Justin B, Kengne Andre P. Burden of Undiagnosed Hypertension in Sub-Saharan Africa. Hypertension. 2015;65:291-8.

60 Hendriks ME, Wit FWNM, Roos MTL, Brewster LM, Akande TM, de Beer IH, et al. Hypertension in Sub-Saharan Africa: Cross-Sectional Surveys in Four Rural and Urban Communities. PLOS ONE. 2012;7:e32638.

61 Musinguzi G, Nuwaha F. Prevalence, Awareness and Control of Hypertension in Uganda. PLOS ONE. 2013;8:e62236.

62 Nuche-Berenguer B, Kupfer LE. Readiness of Sub-Saharan Africa Healthcare Systems for the New Pandemic, Diabetes: A Systematic Review. Journal of Diabetes Research. 2018;2018:9262395.

For peer review only

FIGURES

Figure 1: Mean PHQ-9 scores (z-scored within interviewer) and 95% confidence intervals.

Figure 2: Prevalence of the metabolic syndrome, pre-diabetes, diabetes and hypertension; 95% confidence intervals.

Figure 3: Forest plot of associations between non-communicable conditions and PHQ-9 z-scores.

For peer review only

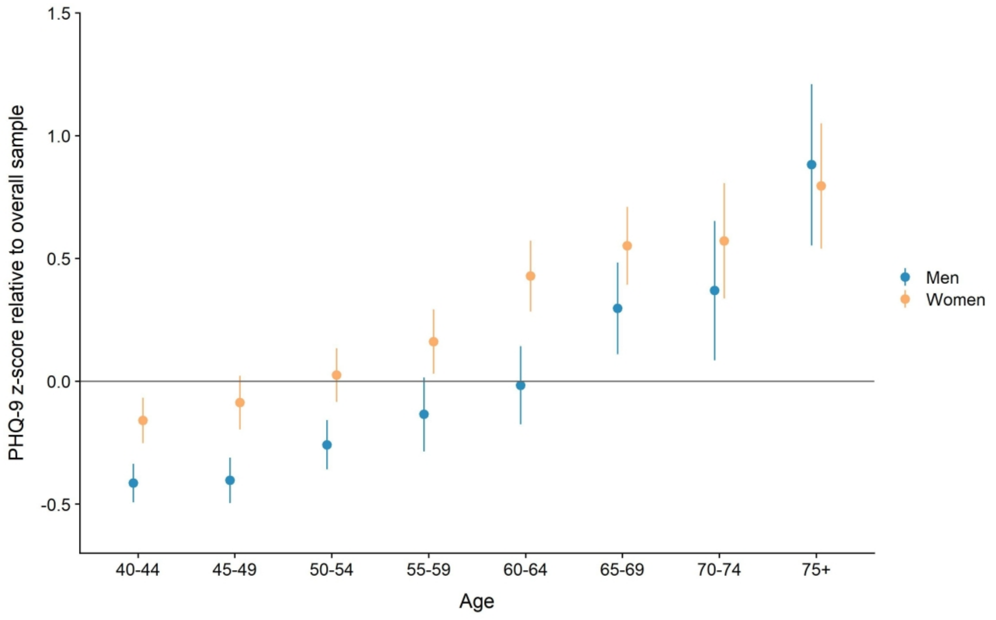

Figure 1: Mean PHQ-9 scores (z-scored within interviewer) and 95% confidence intervals. Underlying data can be found in Supplementary Materials (Supplementary Table 21).

246x152mm (300 x 300 DPI)

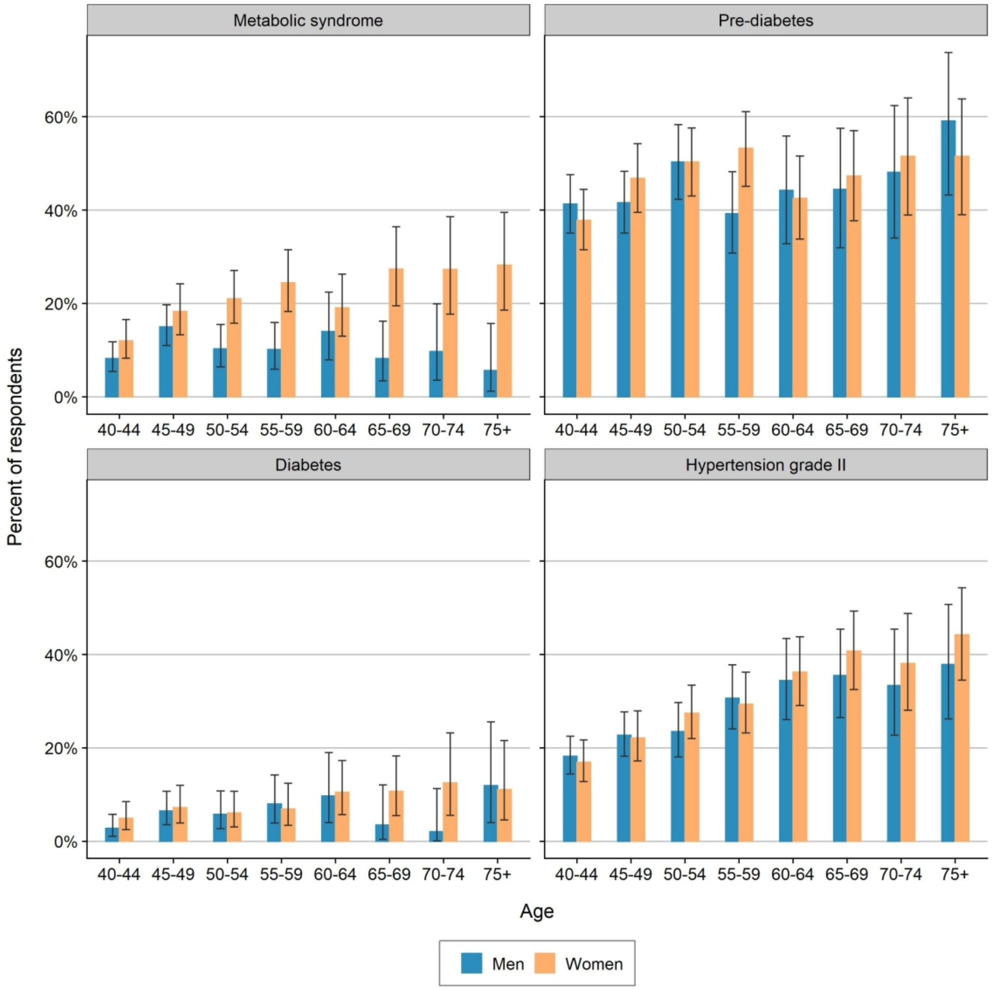

Figure 2: Prevalence of the metabolic syndrome, pre-diabetes, diabetes and hypertension grade II; 95% confidence intervals. Underlying data can be found in Supplementary Materials (Supplementary Tables 22 – 25).

254x254mm (300 x 300 DPI)

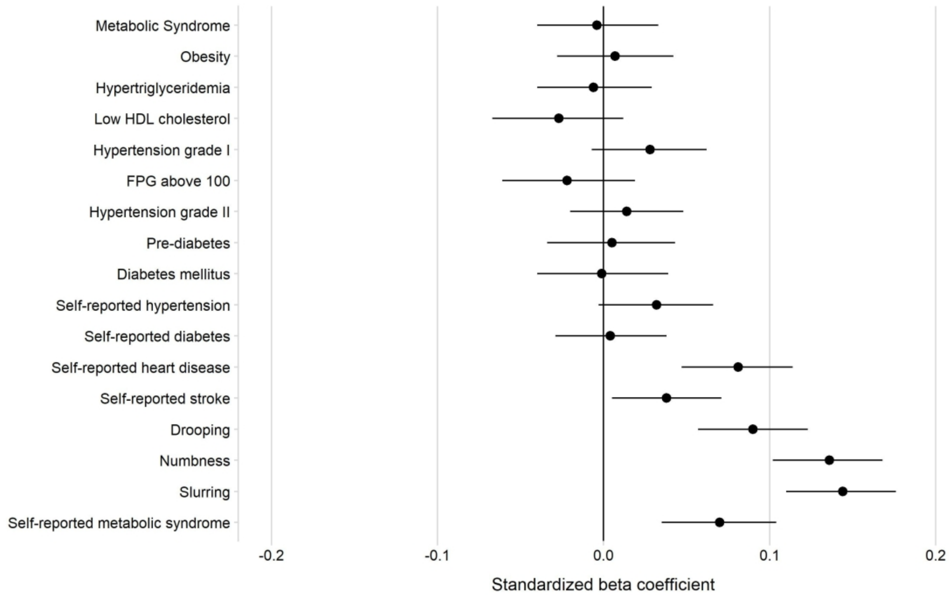

Figure 3: Forest plot of associations between non-communicable conditions and PHQ-9 z-scores.

246x152mm (300 x 300 DPI)

SUPPLEMENTARY MATERIAL

Supplementary Figure 1: This figure shows the distribution of the mean raw PHQ-9 values by single interviewers. The differences between interviewers' PHQ-9 values were interpreted as an interviewer effect during analyses. We decided to standardize the results on the interviewer level and add them up afterwards.

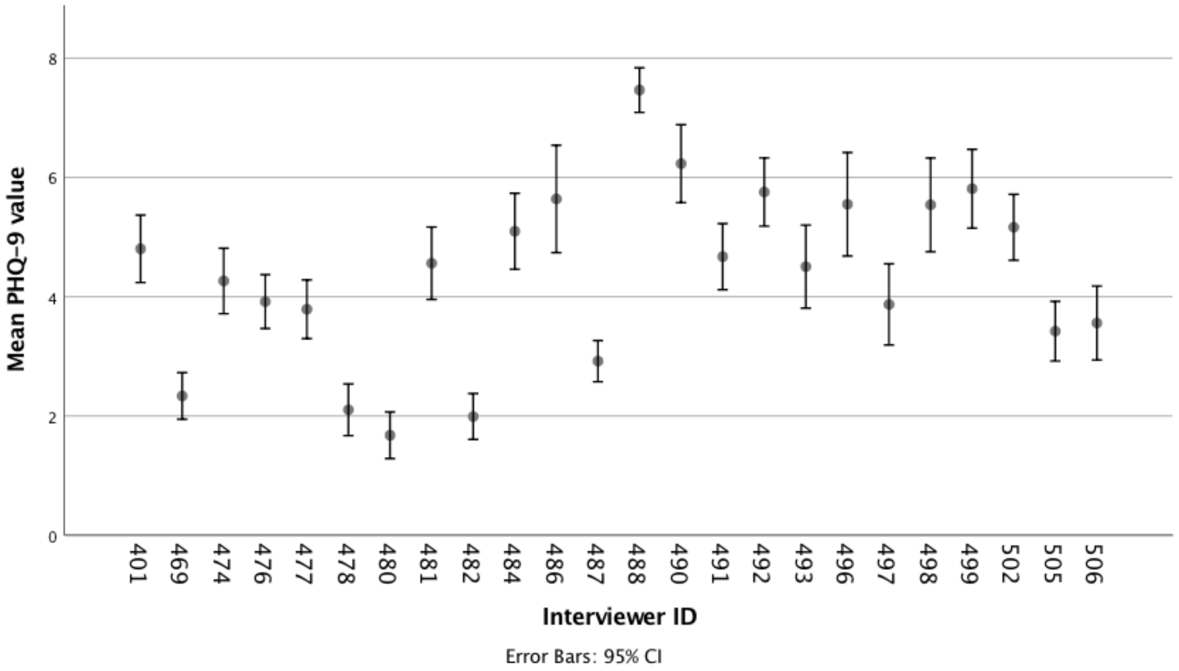

Supplementary Figure 2: The figure shows the standardized PHQ-9 values of one of the interviewers (401) and the normal distribution curve. We z-scored the PHQ-9 results for this interviewer with the standardization function in SPSS. Each value was calculated by dividing the difference between the value and the mean by the standard deviation. This was done for all interviewers separately. Afterwards, all values were included in the outcome variable of the z-scored PHQ-9 score.

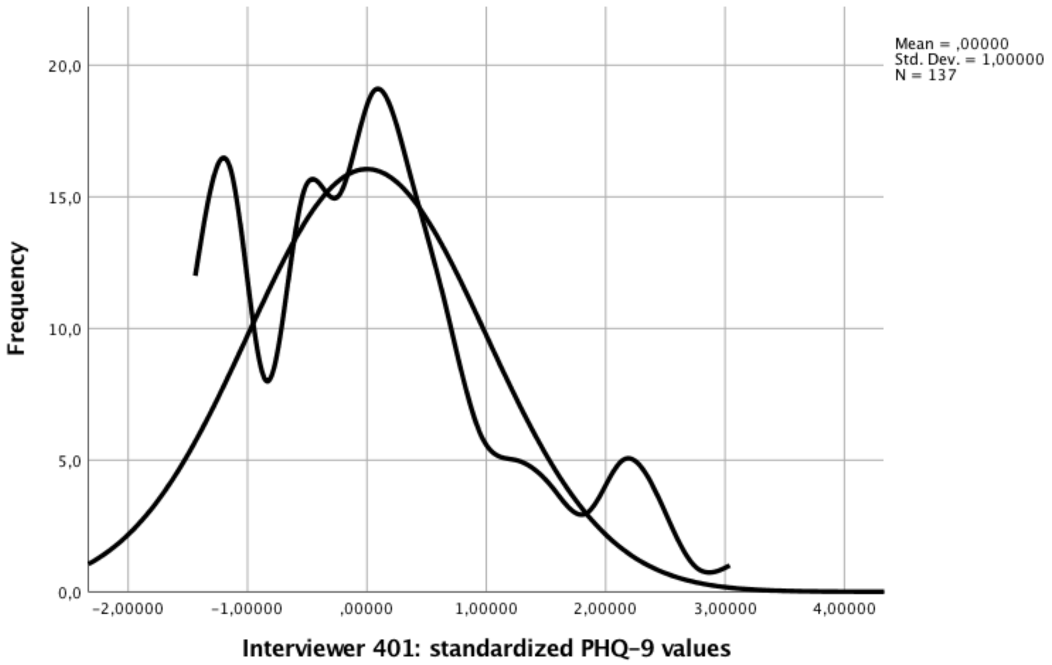

Supplementary Table 1: Distribution of the z-scored PHQ-9 values

		PHQ-9 value	CI lower	CI upper	2 Std. Dev.
m	40-44	-0,414	-0,493	-0,336	0,078
w	40-44	-0,160	-0,252	-0,067	0,092
m	45-49	-0,403	-0,496	-0,311	0,092
w	45-49	-0,087	-0,196	0,022	0,109
m	50-54	-0,259	-0,359	-0,158	0,100
w	50-54	0,025	-0,084	0,135	0,110
m	55-59	-0,135	-0,285	0,015	0,150
w	55-59	0,162	0,030	0,293	0,131
m	60-64	-0,016	-0,175	0,142	0,159
w	60-64	0,428	0,284	0,572	0,144
m	65-69	0,297	0,111	0,484	0,186
w	65-69	0,552	0,393	0,710	0,159
m	70-74	0,369	0,085	0,653	0,284
w	70-74	0,571	0,337	0,806	0,235
m	75+	0,881	0,553	1,210	0,328
w	75+	0,795	0,541	1,050	0,254

For peer review only

**Supplementary Table 2:** Prevalence of the metabolic syndrome				
		Proportion	CI lower	CI upper
m	40-44	0,082	0,054	0,118
w	40-44	0,120	0,083	0,166
m	45-49	0,150	0,110	0,197
w	45-49	0,183	0,133	0,242
m	50-54	0,103	0,064	0,155
w	50-54	0,210	0,158	0,271
m	55-59	0,101	0,059	0,159
w	55-59	0,244	0,183	0,315
m	60-64	0,140	0,079	0,224
w	60-64	0,190	0,130	0,263
m	65-69	0,082	0,034	0,162
w	65-69	0,274	0,195	0,364
m	70-74	0,097	0,036	0,199
w	70-74	0,273	0,177	0,386
m	75+	0,057	0,012	0,157
w	75+	0,282	0,186	0,395

For peer review only

**Supplementary Table 3:** Prevalence of a positive screening result for pre-diabetes				
		Proportion	CI lower	CI upper
m	40-44	0,413	0,351	0,476
w	40-44	0,378	0,315	0,444
m	45-49	0,416	0,351	0,483
w	45-49	0,468	0,395	0,542
m	50-54	0,503	0,423	0,583
w	50-54	0,503	0,430	0,576
m	55-59	0,392	0,308	0,482
w	55-59	0,532	0,451	0,611
m	60-64	0,442	0,328	0,559
w	60-64	0,425	0,338	0,516
m	65-69	0,444	0,319	0,575
w	65-69	0,473	0,377	0,570
m	70-74	0,481	0,340	0,624
w	70-74	0,515	0,389	0,640
m	75+	0,591	0,432	0,737
w	75+	0,515	0,390	0,638

For peer review only

**Supplementary Table 4:** Prevalence of a positive screening result for diabetes mellitus				
		Proportion	CI lower	CI upper
m	40-44	0,028	0,011	0,058
w	40-44	0,049	0,025	0,085
m	45-49	0,065	0,036	0,107
w	45-49	0,072	0,039	0,120
m	50-54	0,058	0,027	0,108
w	50-54	0,061	0,031	0,107
m	55-59	0,080	0,039	0,142
w	55-59	0,069	0,034	0,124
m	60-64	0,097	0,040	0,190
w	60-64	0,105	0,057	0,173
m	65-69	0,035	0,004	0,121
w	65-69	0,107	0,055	0,183
m	70-74	0,021	0,001	0,113
w	70-74	0,125	0,056	0,232
m	75+	0,119	0,040	0,256
w	75+	0,111	0,046	0,216

For peer review only

**Supplementary Table 5:** Prevalence of a positive screening result for hypertension gr. II				
		Proportion	CI lower	CI upper
m	40-44	0,182	0,144	0,225
w	40-44	0,169	0,128	0,217
m	45-49	0,227	0,182	0,277
w	45-49	0,222	0,172	0,279
m	50-54	0,235	0,181	0,297
w	50-54	0,274	0,220	0,334
m	55-59	0,306	0,241	0,378
w	55-59	0,294	0,232	0,362
m	60-64	0,344	0,261	0,434
w	60-64	0,362	0,291	0,438
m	65-69	0,355	0,265	0,454
w	65-69	0,407	0,325	0,493
m	70-74	0,333	0,227	0,454
w	70-74	0,380	0,281	0,488
m	75+	0,379	0,262	0,507
w	75+	0,442	0,345	0,543

For peer review only

Supplementary Table 6: Linear regression model 1 (Metabolic syndrome)

Dependent variable: Z-scored PHQ-9

		Model 1 (n=2524)			
		beta	CI low	CI high	p
Age	40-44	-	-	-	-
	45-49	.007	-.037	.050	.773
	50-54	.058	.014	.098	.010
	55-59	.098	.053	.136	<.001
	60-64	.144	.101	.184	<.001
	65-69	.182	.139	.221	<.001
	70-74	.147	.104	.183	<.001
	75+	.251	.215	.298	<.001
Sex (ref: male)		.115	.074	.151	<.001
Living in the countryside		-.052	-.088	-.011	.013
Currently married		-.020	-.065	.022	.340
Education	None				
	Some	.018	-.020	.054	.360
	Primary & more	-.049	-.088	-.009	.016
Ethnicity	Dafin				
	Bwama	.017	-.022	.053	.430
	Mossi	.007	-.028	.040	.734
	Other	.022	-.024	.082	.280
Wealth quintile	1				
	2	-.030	-.074	.016	.208
	3	-.051	-.096	-.003	.038
	4	-.058	-.103	-.009	.019
	5	-.083	-.135	-.033	.001
**Metabolic syndrome**		-.004	-.040	.033	.847

For peer review only

Supplementary Table 7: Linear regression model 2
Abdominal Obesity

Dependent variable: Z-scored PHQ-9

		Model 2 (n=2970)			
		beta	CI low	CI high	p
Age	40-44	-	-	-	-
	45-49	.017	-.024	.058	.406
	50-54	.059	.019	.099	.004
	55-59	.101	.062	.140	<.001
	60-64	.143	.104	.181	<.001
	65-69	.189	.150	.226	<.001
	70-74	.164	.126	.200	<.001
	75+	.239	.200	.275	<.001
Sex (ref: male)		.118	.081	.153	<.001
Living in the countryside		-.047	-.078	-.008	.016
Currently married		-.028	-.072	.010	.143
Education	None				
	Some	.019	-.016	.053	.288
	Primary & more	-.033	-.071	.003	.072
Ethnicity	Dafin				
	Bwama	.021	-.017	.055	.302
	Mossi	-.001	-.033	.031	.944
	Other	.026	-.015	.080	.180
Wealth quintile	1				
	2	-.020	-.062	.023	.372
	3	-.056	-.100	-.012	.013
	4	-.066	-.110	-.021	.004
	5	-.087	-.135	-.040	<.001
**Abdominal Obesity**		.007	-.028	.042	.680

For peer review only

Supplementary Table 8: Linear regression model 3 (Hypertriglyceridemia)

Dependent variable: Z-scored PHQ-9

		Model 3 (n=2890)			
		beta	CI low	CI high	p
Age	40-44	-	-	-	-
	45-49	.013	-.028	.055	.531
	50-54	.064	.022	.104	.002
	55-59	.098	.057	.137	<.001
	60-64	.144	.103	.182	<.001
	65-69	.186	.144	.221	<.001
	70-74	.159	.120	.194	<.001
	75+	.243	.203	.280	<.001
Sex (ref: male)		.115	.078	.150	<.001
Living in the countryside		-.052	-.083	-.012	.009
Currently married		-.029	-.073	.010	.141
Education	None				
	Some	.017	-.018	.052	.330
	Primary & more	-.041	-.079	-.004	.029
Ethnicity	Dafin				
	Bwama	.015	-.023	.050	.456
	Mossi	.001	-.031	.034	.938
	Other	.025	-.017	.080	.200
Wealth quintile	1				
	2	-.024	-.067	.020	.285
	3	-.054	-.099	-.010	.017
	4	-.069	-.113	-.023	.003
	5	-.083	-.132	-.036	.001
**Hypertriglyceridemia**		-.006	-.040	.029	.742

For peer review only

Supplementary Table 9: Linear regression model 4
(Reduced HDL-cholesterol)

Dependent variable: Z-scored PHQ-9

		Model 4 (n=2330)			
		beta	CI low	CI high	p
Age	40-44	-	-	-	-
	45-49	.019	-.028	.065	.425
	50-54	.061	.015	.106	.009
	55-59	.110	.063	.151	<.001
	60-64	.154	.107	.193	<.001
	65-69	.185	.141	.227	<.001
	70-74	.170	.124	.206	<.001
	75+	.234	.196	.283	<.001
Sex (ref: male)		.134	.091	.174	<.001
Living in the countryside		-.028	-.078	.016	.193
Currently married		-.031	-.079	.013	.158
Education	None				
	Some	.004	-.034	.042	.843
	Primary & more	-.035	-.084	.006	.092
Ethnicity	Dafin				
	Bwama	.007	-.032	.045	.745
	Mossi	-.016	-.051	.022	.437
	Other	.010	-.048	.080	.620
Wealth quintile	1				
	2	-.035	-.080	.012	.147
	3	-.059	-.106	-.010	.017
	4	-.075	-.121	-.025	.003
	5	-.072	-.133	-.026	.004
**Reduced HDL-cholesterol**		-.027	-.067	.012	.177

For peer review only

Supplementary Table 10: Linear regression model 5 (Hypertension grade I)

Dependent variable: Z-scored PHQ-9

		Model 5 (n=2970)			
		beta	CI low	CI high	p
Age	40-44	-	-	-	-
	45-49	.016	-.025	.057	.441
	50-54	.057	.017	.097	.006
	55-59	.098	.058	.137	<.001
	60-64	.139	.099	.177	<.001
	65-69	.185	.145	.222	<.001
	70-74	.161	.123	.197	<.001
	75+	.235	.196	.271	<.001
Sex (ref: male)		.119	.082	.154	<.001
Living in the countryside		-.046	-.077	-.007	.020
Currently married		-.028	-.072	.010	.141
Education	None				
	Some	.020	-.015	.054	.257
	Primary & more	-.034	-.071	.002	.067
Ethnicity	Dafin				
	Bwama	.020	-.018	.054	.326
	Mossi	.000	-.032	.032	.982
	Other	.027	-.014	.081	.164
Wealth quintile	1				
	2	-.019	-.062	.024	.379
	3	-.055	-.099	-.011	.014
	4	-.066	-.110	-.022	.004
	5	-.087	-.134	-.040	<.001
**Hypertension grade I**		.028	-.007	.062	.113

For peer review only

Supplementary Table 11: Linear regression model 6
(Fasting Plasma Glucose > 100 mg/dl)

Dependent variable: Z-scored PHQ-9

		Model 6 (n=2038)			
		beta	CI low	CI high	p
Age	40-44	-	-	-	-
	45-49	.002	-.046	.049	.939
	50-54	.049	.000	.093	.048
	55-59	.102	.053	.144	<.001
	60-64	.143	.096	.188	<.001
	65-69	.192	.144	.236	<.001
	70-74	.125	.078	.164	<.001
	75+	.246	.206	.297	<.001
Sex (ref: male)		.114	.069	.153	<.001
Living in the countryside		-.077	-.114	-.028	.001
Currently married		-.016	-.065	.031	.488
Education	None				
	Some	.024	-.018	.068	.257
	Primary & more	-.066	-.107	-.021	.004
Ethnicity	Dafin				
	Bwama	-.003	-.045	.040	.904
	Mossi	.010	-.028	.045	.663
	Other	.030	-.019	.094	.190
Wealth quintile	1				
	2	-.028	-.078	.024	.295
	3	-.058	-.110	-.004	.035
	4	-.077	-.126	-.021	.006
	5	-.101	-.154	-.042	.001
**Fasting Plasma Glucose > 100 mg/dl**		-.022	-.061	.019	.298

For peer review only

Supplementary Table 12: Linear regression model 7 (Hypertension grade II)

Dependent variable: Z-scored PHQ-9

		Model 7 (n=2970)			
		beta	CI low	CI high	p
Age	40-44	-	-	-	-
	45-49	.017	-.024	.058	.422
	50-54	.059	.018	.099	.004
	55-59	.100	.060	.139	<.001
	60-64	.141	.102	.179	<.001
	65-69	.188	.148	.224	<.001
	70-74	.163	.125	.199	<.001
	75+	.237	.197	.273	<.001
Sex (ref: male)		.118	.082	.154	<.001
Living in the countryside		-.047	-.078	-.008	.016
Currently married		-.028	-.072	.010	.143
Education	None				
	Some	.019	-.015	.054	.274
	Primary & more	-.033	-.070	.003	.072
Ethnicity	Dafin				
	Bwama	.020	-.017	.054	.309
	Mossi	-.001	-.033	.031	.946
	Other	.026	-.015	.081	.176
Wealth quintile	1				
	2	-.019	-.062	.024	.386
	3	-.055	-.099	-.011	.014
	4	-.066	-.109	-.021	.004
	5	-.086	-.133	-.039	<.001
**Hypertension grade II**		.014	-.020	.048	.427

For peer review only

Supplementary Table 13: Linear regression model 8
(Pre-diabetes)

Dependent variable: Z-scored PHQ-9

		Model 8 (n=2143)			
		beta	CI low	CI high	p
Age	40-44	-	-	-	-
	45-49	.005	-.042	.052	.838
	50-54	.050	.002	.093	.040
	55-59	.099	.050	.139	<.001
	60-64	.142	.096	.187	<.001
	65-69	.204	.155	.244	<.001
	70-74	.128	.082	.166	<.001
	75+	.240	.198	.287	<.001
Sex (ref: male)		.110	.066	.148	<.001
Living in the countryside		-.070	-.106	-.022	.003
Currently married		-.018	-.065	.028	.432
Education	None				
	Some	.026	-.015	.067	.216
	Primary & more	-.071	-.110	-.026	.001
Ethnicity	Dafin				
	Bwama	.000	-.041	.041	.999
	Mossi	.007	-.030	.042	.756
	Other	.038	-.008	.102	.097
Wealth quintile	1				
	2	-.024	-.073	.027	.368
	3	-.056	-.106	-.003	.040
	4	-.067	-.115	-.013	.014
	5	-.097	-.148	-.039	.001
**Pre-diabetes**		.005	-.034	.043	.819

For peer review only

Supplementary Table 14: Linear regression model 9 (Diabetes)

Dependent variable: Z-scored PHQ-9

		Model 9 (n=2043)			
		beta	CI low	CI high	p
Age	40-44	-	-	-	-
	45-49	.001	-.047	.048	.983
	50-54	.048	.000	.092	.051
	55-59	.104	.055	.146	<.001
	60-64	.143	.096	.188	<.001
	65-69	.191	.143	.235	<.001
	70-74	.123	.077	.163	<.001
	75+	.246	.204	.295	<.001
Sex (ref: male)		.112	.066	.150	<.001
Living in the countryside		-.081	-.118	-.031	.001
Currently married		-.017	-.065	.030	.474
Education	None				
	Some	.031	-.011	.075	.147
	Primary & more	-.066	-.105	-.020	.004
Ethnicity	Dafin				
	Bwama	-.010	-.051	.033	.672
	Mossi	.008	-.030	.043	.735
	Other	.031	-.018	.095	.179
Wealth quintile	1				
	2	-.033	-.082	.019	.227
	3	-.069	-.120	-.014	.013
	4	-.083	-.132	-.027	.003
	5	-.105	-.158	-.045	<.001
**Diabetes**		-.001	-.040	.039	.972

For peer review only

Supplementary Table 15: Linear regression model 10
(Self-reported hypertension)

Dependent variable: Z-scored PHQ-9

		Model 10 (n=2972)			
		beta	CI low	CI high	p
Age	40-44	-	-	-	-
	45-49	.016	-.025	.057	.434
	50-54	.056	.015	.095	.007
	55-59	.093	.053	.131	<.001
	60-64	.143	.103	.180	<.001
	65-69	.179	.140	.217	<.001
	70-74	.152	.115	.190	<.001
	75+	.235	.196	.271	<.001
Sex (ref: male)		.119	.083	.155	<.001
Living in the countryside		-.044	-.074	-.005	.027
Currently married		-.036	-.080	.003	.066
Education	None				
	Some	.017	-.017	.051	.329
	Primary & more	-.027	-.062	.010	.152
Ethnicity	Dafin				
	Bwama	.017	-.021	.051	.404
	Mossi	.000	-.032	.032	.999
	Other	.026	-.014	.081	.172
Wealth quintile	1				
	2	-.015	-.058	.029	.512
	3	-.050	-.093	-.005	.028
	4	-.060	-.104	-.015	.009
	5	-.087	-.133	-.039	<.001
**Self-reported hypertension**		.032	-.003	.066	.071

For peer review only

Supplementary Table 16: Linear regression model 11
(Self-reported diabetes)

Dependent variable: Z-scored PHQ-9

		Model 11 (n=2991)			
		beta	CI low	CI high	p
Age	40-44	-	-	-	-
	45-49	.013	-.028	.053	.541
	50-54	.058	.018	.097	.005
	55-59	.098	.057	.135	<.001
	60-64	.144	.104	.181	<.001
	65-69	.182	.143	.219	<.001
	70-74	.154	.117	.190	<.001
	75+	.233	.194	.269	<.001
Sex (ref: male)		.123	.086	.158	<.001
Living in the countryside		-.044	-.074	-.005	.024
Currently married		-.036	-.080	.002	.062
Education	None				
	Some	.020	-.014	.054	.250
	Primary & more	-.024	-.060	.012	.195
Ethnicity	Dafin				
	Bwama	.016	-.022	.050	.438
	Mossi	-.001	-.032	.031	.965
	Other	.023	-.018	.076	.230
Wealth quintile	1				
	2	-.015	-.058	.028	.500
	3	-.054	-.097	-.010	.016
	4	-.065	-.108	-.020	.004
	5	-.085	-.130	-.037	<.001
**Self-reported diabetes**		.004	-.029	.038	.809

For peer review only

Supplementary Table 17: Linear regression model 12
(Self-reported heart disease)

Dependent variable: Z-scored PHQ-9

		Model 12 (n=3001)			
		beta	CI low	CI high	p
Age	40-44	-	-	-	-
	45-49	.012	-.029	.052	.567
	50-54	.056	.016	.095	.006
	55-59	.094	.054	.132	<.001
	60-64	.143	.105	.181	<.001
	65-69	.181	.143	.219	<.001
	70-74	.161	.124	.197	<.001
	75+	.236	.197	.272	<.001
Sex (ref: male)		.112	.076	.148	<.001
Living in the countryside		-.047	-.077	-.008	.016
Currently married		-.029	-.072	.009	.128
Education	None				
	Some	.018	-.017	.052	.314
	Primary & more	-.026	-.062	.010	.159
Ethnicity	Dafin				
	Bwama	.016	-.021	.051	.410
	Mossi	-.002	-.033	.030	.931
	Other	.021	-.021	.074	.272
Wealth quintile	1				
	2	-.021	-.063	.022	.338
	3	-.058	-.102	-.015	.009
	4	-.071	-.115	-.027	.002
	5	-.092	-.138	-.045	<.001
**Self-reported heart disease**		.081	.047	.114	<.001

For peer review only

Supplementary Table 18: Linear regression model 13
(Self-reported stroke)

Dependent variable: Z-scored PHQ-9

		Model 13 (n=3026)			
		beta	CI low	CI high	p
Age	40-44	-	-	-	-
	45-49	.015	-.025	.056	.455
	50-54	.058	.018	.097	.004
	55-59	.094	.055	.133	<.001
	60-64	.143	.104	.181	<.001
	65-69	.185	.147	.222	<.001
	70-74	.162	.125	.198	<.001
	75+	.236	.197	.272	<.001
Sex (ref: male)		.122	.086	.157	<.001
Living in the countryside		-.049	-.079	-.010	.012
Currently married		-.031	-.074	.008	.110
Education	None				
	Some	.019	-.015	.053	.282
	Primary & more	-.027	-.062	.009	.147
Ethnicity	Dafin				
	Bwama	.019	-.018	.053	.344
	Mossi	-.001	-.032	.031	.960
	Other	.023	-.018	.076	.224
Wealth quintile	1				
	2	-.018	-.061	.024	.401
	3	-.055	-.098	-.011	.014
	4	-.065	-.109	-.021	.004
	5	-.087	-.134	-.040	<.001
**Self-reported stroke**		.038	.005	.071	.024

For peer review only

Supplementary Table 19: Linear regression model 14
(Sudden drooping)

Dependent variable: Z-scored PHQ-9

		Model 14 (n=3026)			
		beta	CI low	CI high	p
Age	40-44	-	-	-	-
	45-49	.013	-.027	.054	.519
	50-54	.054	.014	.093	.008
	55-59	.094	.055	.132	<.001
	60-64	.137	.099	.175	<.001
	65-69	.183	.144	.220	<.001
	70-74	.157	.120	.193	<.001
	75+	.235	.196	.271	<.001
Sex (ref: male)		.120	.084	.155	<.001
Living in the countryside		-.049	-.078	-.010	.012
Currently married		-.031	-.075	.006	.100
Education	None				
	Some	.019	-.015	.053	.284
	Primary & more	-.027	-.062	.009	.147
Ethnicity	Dafin				
	Bwama	.015	-.021	.049	.438
	Mossi	-.001	-.033	.030	.947
	Other	.022	-.019	.075	.241
Wealth quintile	1				
	2	-.017	-.059	.026	.446
	3	-.056	-.099	-.013	.012
	4	-.066	-.109	-.022	.003
	5	-.087	-.133	-.041	<.001
**Sudden drooping**		.090	.057	.123	<.001

For peer review only

Supplementary Table 20: Linear regression model 15 (Sudden numbness)

Dependent variable: Z-scored PHQ-9

		Model 15 (n=3026)			
		beta	CI low	CI high	p
Age	40-44	-	-	-	-
	45-49	.017	-.024	.057	.419
	50-54	.059	.019	.098	.004
	55-59	.095	.056	.133	<.001
	60-64	.135	.096	.172	<.001
	65-69	.178	.140	.215	<.001
	70-74	.155	.118	.190	<.001
	75+	.227	.189	.263	<.001
Sex (ref: male)		.117	.081	.151	<.001
Living in the countryside		-.051	-.080	-.012	.008
Currently married		-.034	-.078	.003	.071
Education	None				
	Some	.016	-.018	.049	.366
	Primary & more	-.026	-.062	.009	.147
Ethnicity	Dafin				
	Bwama	.017	-.019	.051	.380
	Mossi	.001	-.031	.032	.965
	Other	.020	-.022	.072	.291
Wealth quintile	1				
	2	-.019	-.061	.023	.378
	3	-.061	-.104	-.017	.006
	4	-.072	-.115	-.028	.001
	5	-.097	-.143	-.051	<.001
**Sudden numbness**		.136	.102	.168	<.001

For peer review only

Supplementary Table 21: Linear regression model 16
(Sudden slurring)

Dependent variable: Z-scored PHQ-9

		Model 16 (n=3026)			
		beta	CI low	CI high	p
Age	40-44	-	-	-	-
	45-49	.016	-.025	.055	.448
	50-54	.056	.017	.096	.005
	55-59	.093	.054	.131	<.001
	60-64	.134	.096	.172	<.001
	65-69	.175	.137	.212	<.001
	70-74	.155	.118	.190	<.001
	75+	.227	.189	.263	<.001
Sex (ref: male)		.117	.081	.151	<.001
Living in the countryside		-.052	-.081	-.013	.007
Currently married		-.037	-.081	.000	.050
Education	None				
	Some	.019	-.015	.053	.264
	Primary & more	-.029	-.064	.007	.113
Ethnicity	Dafin				
	Bwama	.017	-.019	.051	.379
	Mossi	.001	-.030	.032	.954
	Other	.017	-.026	.068	.374
Wealth quintile	1				
	2	-.022	-.064	.020	.312
	3	-.060	-.103	-.017	.006
	4	-.072	-.116	-.029	.001
	5	-.091	-.136	-.044	<.001
**Sudden slurring**		.144	.110	.176	<.001

For peer review only

Supplementary Table 22: Linear regression model 17
(Self-reported metabolic syndrome)

Dependent variable: Z-scored PHQ-9

		Model 17 (n=2948)			
		beta	CI low	CI high	p
Age	40-44	-	-	-	-
	45-49	.014	-.027	.055	.497
	50-54	.055	.014	.094	.008
	55-59	.092	.052	.130	<.001
	60-64	.141	.101	.178	<.001
	65-69	.174	.135	.212	<.001
	70-74	.151	.114	.188	<.001
	75+	.230	.190	.266	<.001
Sex (ref: male)		.117	.080	.152	<.001
Living in the countryside		-.039	-.070	.000	.049
Currently married		-.039	-.083	-.001	.045
Education	None				
	Some	.018	-.017	.052	.314
	Primary & more	-.029	-.064	.008	.124
Ethnicity	Dafin				
	Bwama	.015	-.022	.049	.460
	Mossi	-.001	-.033	.031	.956
	Other	.024	-.017	.079	.201
Wealth quintile	1				
	2	-.016	-.059	.027	.459
	3	-.051	-.095	-.007	.024
	4	-.066	-.110	-.021	.004
	5	-.091	-.137	-.042	<.001
**Self-reported metabolic syndrome**		.070	.035	.104	<.001

For peer review only

Supplementary Table 23: Descriptive statistics of the self-reported diagnosis of hypertension and diabetes within the whole sample

	Total	Female	Male
**Self-reported diagnosis of hypertension**	*n* = 3007<br>507 (17.1%)	*n* = 1513<br>294 (19.8%)	*n* = 1494<br>213 (14.4%)
**Self-reported diagnosis of diabetes**	*n* = 2993<br>70 (2.3%)	*n* = 1505<br>32 (2.1%)	*n* = 1488<br>38 (2.6%)

Supplementary Table 24: Descriptive statistics of the self-reported diagnosis of hypertension and diabetes within the affected* part of the sample

	Total	Female	Male
**Self-reported diagnosis of hypertension**	*n* = 1674<br>346 (21.0%)	*n* = 841<br>203 (24.6%)	*n* = 832<br>143 (17.3%)
**Self-reported diagnosis of diabetes**	*n* = 143<br>20 (14.0%)	*n* = 85<br>11 (13.0%)	*n* = 58<br>9 (15.5%)

*In case of Hypertension: Systolic BP >130 mmHg or Diastolic BP >80 mmHg
Diabetes: Fasting Glucose >126 mg/dl, random glucose >200 mg/dl or HbA1c >6.5%

Supplementary Table 25: Measuring the effect of a diagnosis of hypertension/diabetes given by a health care worker among hypertensive/diabetic part of the population.
Given in the table are the standardized coefficients of multiple linear regression of the z-scored PHQ-9 score on self-reported diagnosis of hypertension within the hypertensive population[a] and diagnosis of diabetes within the diabetic population[b].

	Standardized beta coefficient	p-value
**Ever been diagnosed with hypertension**	0.033	0.061
**Ever been diagnosed with diabetes**	-0.025	0.762

All variables used in separate models controlling for age, sex, gender, place of residence, education, ethnicity and wealth quintile.
[a] Systolic BP >130 mmHg or Diastolic BP >80 mmHg
[b] Fasting Glucose >126 mg/dl, random glucose >200 mg/dl or HbA1c >6.5%

STROBE Statement—Checklist of items that should be included in reports of ***cross-sectional studies***

	Item No	Recommendation	Page No
**Title and abstract**	1	(*a*) Indicate the study's design with a commonly used term in the title or the abstract	1
		(*b*) Provide in the abstract an informative and balanced summary of what was done and what was found	3,4
**Introduction**			
Background/rationale	2	Explain the scientific background and rationale for the investigation being reported	6,7
Objectives	3	State specific objectives, including any prespecified hypotheses	7
**Methods**			
Study design	4	Present key elements of study design early in the paper	8
Setting	5	Describe the setting, locations, and relevant dates, including periods of recruitment, exposure, follow-up, and data collection	8
Participants	6	(*a*) Give the eligibility criteria, and the sources and methods of selection of participants	8
Variables	7	Clearly define all outcomes, exposures, predictors, potential confounders, and effect modifiers. Give diagnostic criteria, if applicable	9,10,11,12
Data sources/ measurement	8*	For each variable of interest, give sources of data and details of methods of assessment (measurement). Describe comparability of assessment methods if there is more than one group	8,9
Bias	9	Describe any efforts to address potential sources of bias	10
Study size	10	Explain how the study size was arrived at	8
Quantitative variables	11	Explain how quantitative variables were handled in the analyses. If applicable, describe which groupings were chosen and why	11
Statistical methods	12	(*a*) Describe all statistical methods, including those used to control for confounding	11
		(*b*) Describe any methods used to examine subgroups and interactions	11
		(*c*) Explain how missing data were addressed	12
		(*d*) If applicable, describe analytical methods taking account of sampling strategy	8
		(*e*) Describe any sensitivity analyses	N/A
**Results**			
Participants	13*	(a) Report numbers of individuals at each stage of study—eg numbers potentially eligible, examined for eligibility, confirmed eligible, included in the study, completing follow-up, and analysed	12
		(b) Give reasons for non-participation at each stage	8
		(c) Consider use of a flow diagram	N/A
Descriptive data	14*	(a) Give characteristics of study participants (eg demographic, clinical, social) and information on exposures and potential confounders	13,14
		(b) Indicate number of participants with missing data for each variable of interest	14
Outcome data	15*	Report numbers of outcome events or summary measures	13

For peer review only

Main results	16	(*a*) Give unadjusted estimates and, if applicable, confounder-adjusted estimates and their precision (eg, 95% confidence interval). Make clear which confounders were adjusted for and why they were included	15
		(*b*) Report category boundaries when continuous variables were categorized	12
		(*c*) If relevant, consider translating estimates of relative risk into absolute risk for a meaningful time period	N/A
Other analyses	17	Report other analyses done—eg analyses of subgroups and interactions, and sensitivity analyses	16
**Discussion**			
Key results	18	Summarise key results with reference to study objectives	16,17
Limitations	19	Discuss limitations of the study, taking into account sources of potential bias or imprecision. Discuss both direction and magnitude of any potential bias	5,20,21
Interpretation	20	Give a cautious overall interpretation of results considering objectives, limitations, multiplicity of analyses, results from similar studies, and other relevant evidence	16,17,18,19
Generalisability	21	Discuss the generalisability (external validity) of the study results	16,17,18,19
**Other information**			
Funding	22	Give the source of funding and the role of the funders for the present study and, if applicable, for the original study on which the present article is based	21,22

*Give information separately for exposed and unexposed groups.

Note: An Explanation and Elaboration article discusses each checklist item and gives methodological background and published examples of transparent reporting. The STROBE checklist is best used in conjunction with this article (freely available on the Web sites of PLoS Medicine at http://www.plosmedicine.org/, Annals of Internal Medicine at http://www.annals.org/, and Epidemiology at http://www.epidem.com/). Information on the STROBE Initiative is available at www.strobe-statement.org.

For peer review only
